# Parkinson’s disease and cancer: a systematic review and meta-analysis of over 17 million participants

**DOI:** 10.1136/bmjopen-2020-046329

**Published:** 2021-07-02

**Authors:** Xinyuan Zhang, David Guarin, Niyaz Mohammadzadehhonarvar, Xiqun Chen, Xiang Gao

**Affiliations:** 1 Nutritional Science, The Pennsylvania State University, University Park, Pennsylvania, USA; 2 Neurology, Massachusetts General Hospital, Boston, Massachusetts, USA

**Keywords:** neoplasms, epidemiology, meta-analysis, parkinson’s disease, odds ratio

## Abstract

**Objective:**

To systematically review and qualitatively evaluate epidemiological evidence on associations between Parkinson’s disease (PD) and cancer via meta-analysis.

**Data sources:**

MEDLINE via PubMed, Web of Science and EMBASE, until March 2021.

**Study selection:**

Included were publications that (1) were original epidemiological studies on PD and cancer; (2) reported risk estimates; (3) were in English. Exclusion criteria included: (1) review/comments; (2) biological studies; (3) case report/autopsy studies; (4) irrelevant exposure/outcome; (5) treated cases; (6) no measure of risk estimates; (7) no confidence intervals/exact p values and (8) duplicates.

**Data extraction and synthesis:**

PRISMA and MOOSE guidelines were followed in data extraction. Two-step screening was performed by two authors blinded to each other. A random-effects model was used to calculate pooled relative risk (RR).

**Main outcomes and measures:**

We included publications that assessed the risk of PD in individuals with vs without cancer and the risk of cancer in individuals with vs without PD.

**Results:**

A total of 63 studies and 17 994 584 participants were included. Meta-analysis generated a pooled RR of 0.82 (n=33; 95% CI 0.76 to 0.88; p<0.001) for association between PD and total cancer, 0.76 (n=21; 95% CI 0.67 to 0.85; p<0.001) for PD and smoking-related cancer and 0.92 (n=19; 95% CI 0.84 to 0.99; p=0.03) for non-smoking-related cancer. PD was associated with an increased risk of melanoma (n=29; pooled RR=1.75; 95% CI 1.43 to 2.14; p<0.001) but not for other skin cancers (n=17; pooled RR=0.90; 95% CI 0.60 to 1.34; p=0.60).

**Conclusions:**

PD and total cancer were inversely associated. This inverse association persisted for both smoking-related and non-smoking-related cancers. PD was positively associated with melanoma. These results provide evidence for further investigations for possible mechanistic associations between PD and cancer.

**Prospero registration number:**

CRD42020162103.

**Strengths and limitations of this study**

- Unlike recent meta-analyses, this study stratifies analysis for smoking vs non-smoking cancers.
- Heterogeneity between included studies was analyzed via meta-regression.
- Despite best efforts, high heterogeneity in methodology and cohorts of included studies cannot be fully dealt with by statistical methods.

## Introduction

Parkinson’s disease (PD) is the second most common neurodegenerative disease affecting more than 10 milion people worldwide. It is characterised by premature cell death of dopaminergic neurons in the substantia nigra pars compacta. Clinically, PD is manifested by tremor, rigidity, bradykinesia and postural instability. Non-motor symptoms are also common. Symptomatic treatments for PD are available and effective, however, there is currently no therapy known to modify disease progression. Among enviromental factors that have been associated with the risk of developing PD, age is the main risk factor, whereas smoking has been inversely associated with PD.^1 2^ Familial PD accounts for 5%–15% of total PD. The most common genetic cause of PD is mutations in *LRRK2*. Other PD-related genes include *PARK2*, *PARK7*, *PINK1* and *SNCA*. PD is increasingly recognised as a systemic disorder. Oxidative stress, mitochondria dysfuntion, energy failure, immune dysregulation and chronic inflammation have been proposed to contribute to neurodegeneration in PD.^3^

Cancer is characterised by uncontrolled cell proliferation and growth. It is among the leading causes of death worldwide.^4^ Growing evidence suggests that PD and cancer may be associated.^5^ Similar to PD, cancer incidence increases with age.^6^ Smoking also modifies the risk of certain cancer, especially lung cancer, though in the opposite direction to the risk of PD.^7^ In addition, PD-related genes have been implicated in cancer. *PARK2* has been identified as a potent tumour suppressor gene, whereas mutations in *LRRK2* have been associated with an increased risk of cancer.^8^ While a positive, bidirectional link between PD and melanoma, a malignant tumour that develops from melanocytes is well documented,^9^ there appears to be an inverse association between PD and total cancer.^10^ However, it remains unclear whether PD and cancer are associated mechanistically, or the findings were confounded by other factors, such as study designs and smoking. Clearly documenting these associations is important for bridging the interdisciplinary knowledge gap and developing novel preventive and treatment strategies for both PD and cancer. An individual study may lack the power to detect an association. A meta-analysis can increase precision in estimating risk,^11^ especially in subsets of cancers with even fewer cases. We, thus, conducted a meta-analysis to systematically review the population-based evidence for the potential association between PD and cancer. To better elucidate PD-cancer relation, we first stratified studies according to the temporal association between the two diseases into three categories: PD preceding cancer, cancer preceding PD and co-occurrence. Second, we performed sensitivity analyses in which variations in study design and qualities, and levodopa treatment, were evaluated. Third, we separately analysed smoking-related cancers and non-smoking-related cancers to address smoking as a potential confounding factor. Finally, we specifically analysed the associations between PD and melanoma, non-melanoma skin cancers and other major cancers (eg, prostate cancer, colon cancer and breast cancer).

## Methods

### Literature search and data extraction

This meta-analysis followed the MOOSE guidelines for reporting meta-analysis on observational studies and was registered on PROSPERO (CRD42020162103). We searched all published literature that reported PD association with cancer in MEDLINE via PubMed, Web of Science and EMBASE up to 1 March 2021. Search items related to ‘PD’, ‘cancer’ and ‘epidemiological studies’ were identified and modified for each database. We constrained our search in human studies and in the English language. Detailed search terms can be found in online supplemental materials. Duplicates were matched based on author, year, and title in Endnote X9 and manually compared before removing.

10.1136/bmjopen-2020-046329.supp1 Supplementary data

The inclusion criteria were: (1) original studies that were conducted in an epidemiological setting; (2) studies reported either an OR, relative risk (RR), HR, standardised incidence/mortality ratio (SIR/SMR) or other reliable measures of estimated risk; (3) studies in which PD and cancer cases were ascertained by doctor’s diagnosis, hospitalisation record, disease identification codes or self-report on the diagnosis. Exclusion criteria included: (1) reviews or comments; (2) non-epidemiological studies; (3) case reports/autopsy studies; (4) irrelevant exposure/outcome; (5) treated cases; (6) no measure of risk estimates; (7) no confidence intervals/exact p values and (8) duplicates. Parkinsonism that does not meet the criteria for PD and benign neoplasm were not included. Previous meta-analyses were used as references for manual searching of related publications. Two first authors (XZ and DG) independently screened the publications in two steps: title/abstract screening and full-text screening. Any discrepancy was reviewed and reconciled by two senior authors (XC and XG). During full-text screening, we found five groups of publications using the same population or dataset. Details of inclusion and exclusion step are reported in online supplemental methods. After screening references of included publications, we found two other eligible publications that were not captured by search items.^12 13^

### Data extraction

From each of the included publications, we extracted information on the first author, year of the study, study type, country origin, population, mean age, dominant sex, dominant ethnicity, cases and controls population size, measure of risk, PD and cancer ascertainment methods, adjusted covariates, levodopa use and estimated risk with lower and upper CIs for each type of cancer. The temporal association was defined per each individual study definition, most of which was based on the diagnosis date of the two diseases. Dominant sex and ethnicity were defined as the major sex and race/ethnicity (>50%) of the studied population, respectively.

The type of study was categorised into prospective study, case–control study, case-only cohort study and cross-sectional study.

### Statistical analysis

All analyses were performed in STATA SE V.15. Cochran’s Q statistic and I^2^ were calculated to examine heterogeneity among studies. Cochran’s Q was computed as the sum of variance from the pooled estimates and compared with chi-squared distribution with *k*-1 (*k*=number of publications) degree of freedom. I^2^ was calculated as the percentage of variation across studies due to heterogeneity rather than chance.^14^ Due to the high heterogeneity of included publications (p value for Q statistics <0.05, I^2^ >50% for all), pooled effect sizes (including RR, OR, HR, SIR and SMR) were calculated using random-effects models to account for unobserved heterogeneity. Egger test and funnel plots were performed to assess publication bias.

For total cancer, we performed three sensitivity analyses. First, four publications from meeting proceedings/abstracts were further included; second, eight mortality publications were excluded; third, two publications using invalidated, self-report diagnosis of either cancer or PD were excluded. Further, we performed six subgroup analyses, looking at the variance of the included publications in population age, dominant sex, dominant race/ethnicity, study design, study quality and year of study. Age was separated into two groups by the mean age of the included studies (69.3 years). Dominant ethnicity was categorised into Caucasian-dominant and Asian-dominant. The study design was categorised into cohort studies and other types of studies. Study quality was assessed by the Newcastle-Ottawa Scale for cohort studies and for case–control studies,^15^ which is based on the definition of case/control, the definition of exposure/outcome, covariates and other relevant factors. The score ranged from 0 to 9, and we separated the included studies into low-quality group (<7) and high-quality group (>7), based on the mean quality score of the included studies. Proceedings/abstracts were not included in the quality check. The difference between groups was tested by the meta-regression method.

We categorised cancers into smoking-related and non-smoking-related cancers according to National Cancer Institute and Centers for Disease Control and Prevention’s definition.^16^ Smoking-related cancers include cancer of the lung, larynx, mouth, oesophagus, throat, bladder, kidney, liver, stomach, pancreas, colon and rectum, and cervix, as well as acute myeloid leukaemia. Cancers of other sites, including melanoma, were regarded as not associated with smoking. If a publication reported grouped smoking- and non-smoking-related cancers, the risk estimates were extracted directly. If a publication reported individual cancers only, and the number of sites is more than 10, we first categorised individual cancers into smoking-related and non-smoking-related groups accordingly,^16^ calculated pooled RR and 95% CI in each group using a random-effects model, and then included the resulting pooled RR in the final meta-analysis.

We specifically evaluated the association between PD and melanoma, and other skin cancers. Cancers of other specific sites were included in this meta-analysis if there were more than 10 publications. Included were lung cancer, colorectal cancer, breast cancer and prostate cancer.

## Results

**Figure 1:**
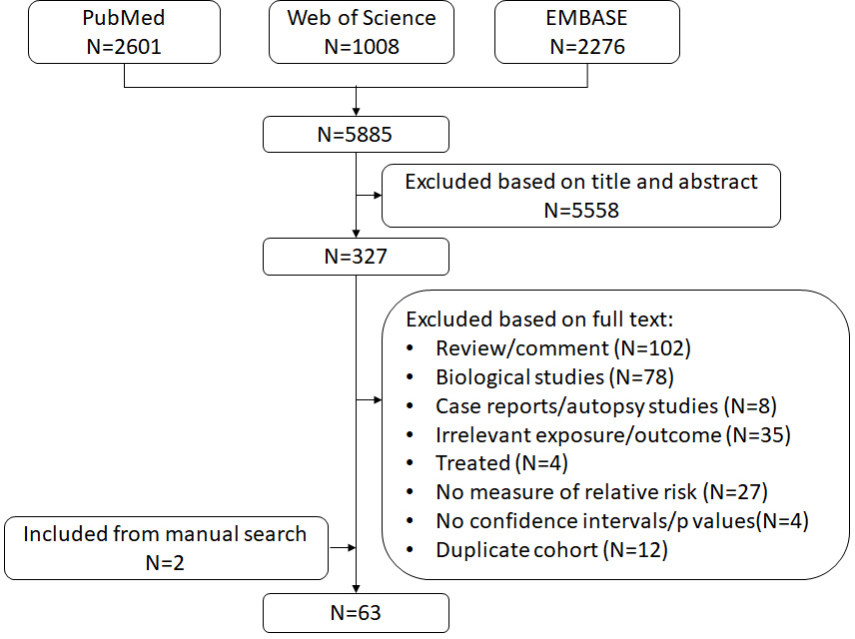
Flow chart.

In total, we included 63 publications in this meta-analysis (figure 1).^12 13 17–77^ Characteristics of all publications are listed in online supplemental table 1.

### PD and total cancer

**Figure 2:**
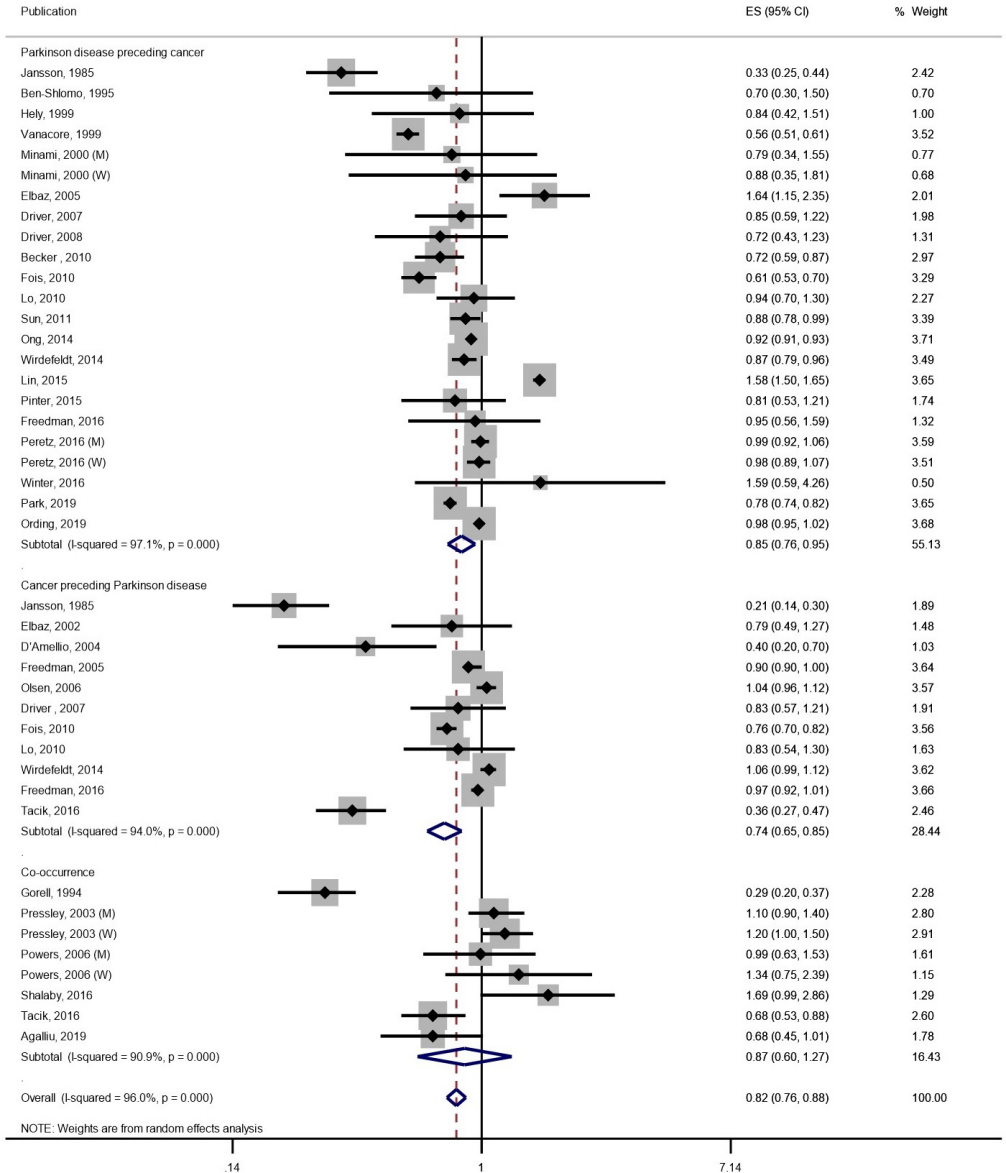
Association between Parkinson’s disease and total cancer in 33 publications. The figure shows the estimates (ESs) and 95% CIs for each study and the pooled result from random-effects model. Studies are stratified by temporal relationship of Parkinson’s disease and cancer. M, men; W, women.

Combining 33 publications,^12 13 18 27–32 35–39 41 50–52 54 57–62 64 65 69–71 73 75 76^ pooled RR for association between PD and cancer was 0.82 (95% CI 0.76 to 0.88; p<0.001; figure 2). We did not observe evidence for existence of publication bias (Egger test p=0.27; online supplemental figure 1). After stratified by temporal sequence, PD was significantly associated with a lower future risk of cancer (n=21, pooled RR=0.85; 95% CI 0.76 to 0.95; p=0.004), and similar association was observed for cancer with a lower future risk of PD (n=11, pooled RR=0.74; 95% CI 0.65 to 0.85; p =<0.001). The significant inverse association persisted after further including meeting abstracts, excluding mortality studies and excluding self-report outcomes that were not validated (table 1). Meta-regression did not find significant difference between subgroups stratified by age (<69.3 years vs ≥69.3 years; mean value of the included studies), sex (men-dominant vs women-dominant cohorts), ethnicity (Caucasian vs Asian), study design (cohort vs others), study quality (scored <7 vs ≥7) or year of study (before 2010, or 2010 and after (online supplemental table 2).

**Table 1:** Association between Parkinson’s disease and cancer

	No of publications	Pooled RR (95% CI)	P for significance	P for heterogeneity
Total cancer				
All full-text publications	33	0.82 (0.76 to 0.88)	<0.001	<0.001
Including abstracts	37	0.80 (0.74 to 0.86)	<0.001	<0.001
Excluding mortality studies	25	0.85 (0.79 to 0.92)	<0.001	<0.001
Excluding self-report diagnosis	31	0.81 (0.75 to 0.87)	<0.001	<0.001
Smoking-related cancer**	21	0.76 (0.67 to 0.85)	<0.001	<0.001
Non-smoking-related cancer††	19	0.92 (0.84 to 0.99)	0.03	<0.001
Site-specific cancer				
Melanoma	29	1.75 (1.43 to 2.14)	<0.001	<0.001
Non-melanoma skin cancer	17	0.90 (0.60 to 1.34)	0.60	<0.001
Lung cancer	20	0.62 (0.51 to 0.75)	<0.001	<0.001
Colorectal cancer	20	0.82 (0.75 to 0.90)	<0.001	<0.001
Breast cancer	15	1.02 (0.93 to 1.12)	0.66	0.001
Prostate cancer	17	0.93 (0.83 to 1.03)	0.18	<0.001

*Smoking-related cancer includes cancer of the lung, larynx, mouth, oesophagus, throat, bladder, kidney, liver, stomach, pancreas, colon and rectum, and cervix, as well as acute myeloid leukaemia.

†Non-smoking-related cancer includes all other cancer except for those listed as smoking related.

RR, relative risk.;

We found four publications that examined the risk of cancers associated with the treatment of levodopa in PD patients (online supplemental table 3).^18 24 31 56^ Although there was a significant lower risk of cancer after levodopa treatment or with higher cumulative levodopa treatment (pooled RR=0.75; 95% CI 0.61 to 0.92; p=0.007; online supplemental figure 2a), Egger test (p=0.005) and funnel plot (online supplemental figure 2b) showed a significant publication bias and thus a potentially overestimated result.

### Smoking-related and non-smoking-related cancers

**Figure 3:**
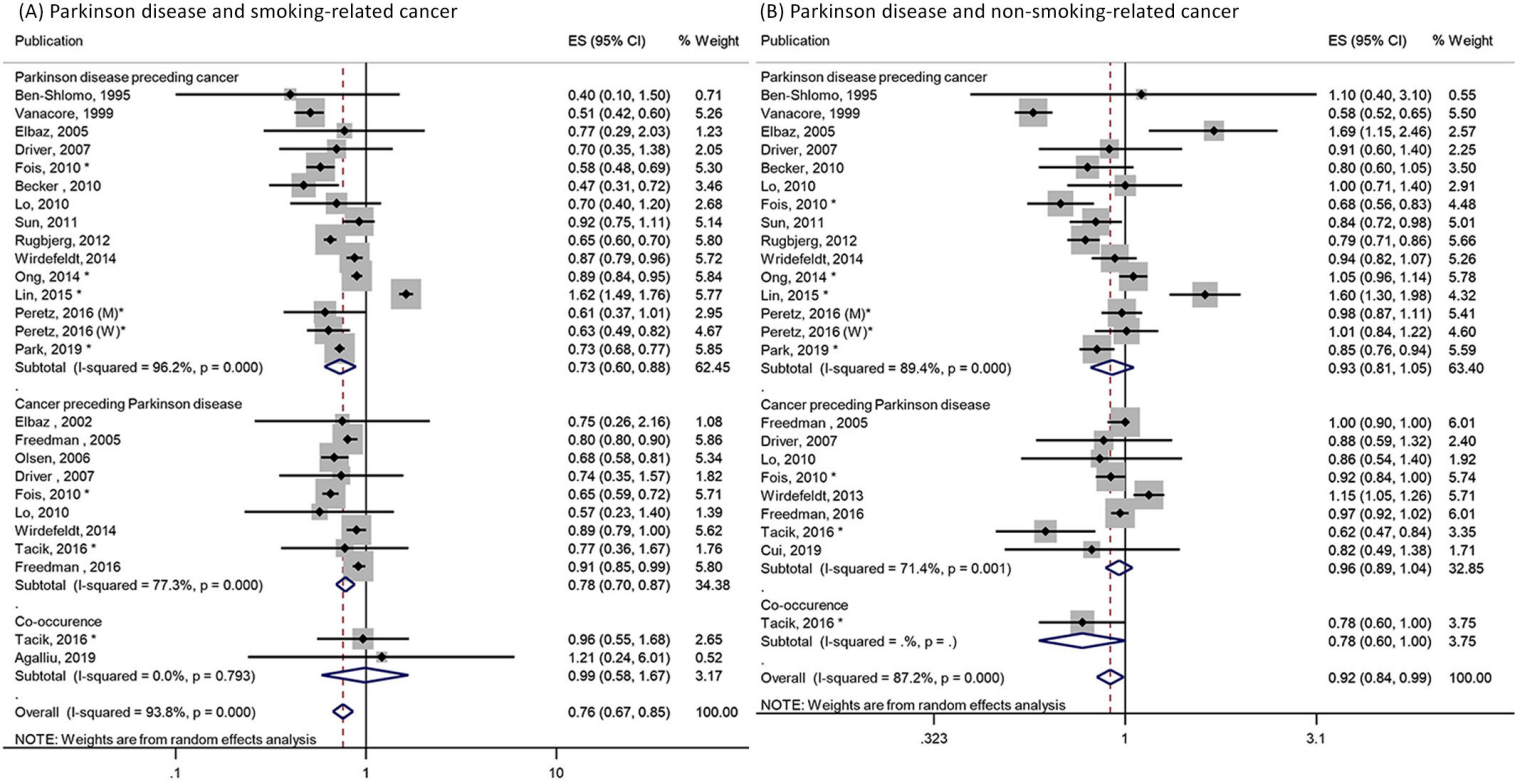
Association between Parkinson’s disease and (A) smoking-related cancers in 21 publications and (B) non-smoking-related cancers in 19 publications. Figure shows the estimates (ESs) and 95% CIs for each study and the pooled result from random effects model. Studies are stratified by temporal relationship of Parkinson’s disease and cancer. *Pooled risk estimates calculated from individual ES in original publication. M, men; W, women.

Combining 21 publications,^12 18 28 30–32 35 37 38 50 51 54 57–60 66 70 71 73 76^ the pooled RR for association between PD and smoking-related cancers was 0.76 (95% CI 0.67 to 0.85; p<0.001; figure 3A). PD was also inversely associated with non-smoking-related cancers (n=19; pooled RR=0.92; 95% CI 0.84 to 0.99; p=0.03; figure 3b).^12 18 25 28 30 31 35 37 38 50 51 57–59 66 70 71 73 76^ No publication bias was observed for both analyses (Egger test p=0.45 and 0.50, respectively; online supplemental figure 3).

### Melanoma and non-melanoma skin cancer

**Figure 4:**
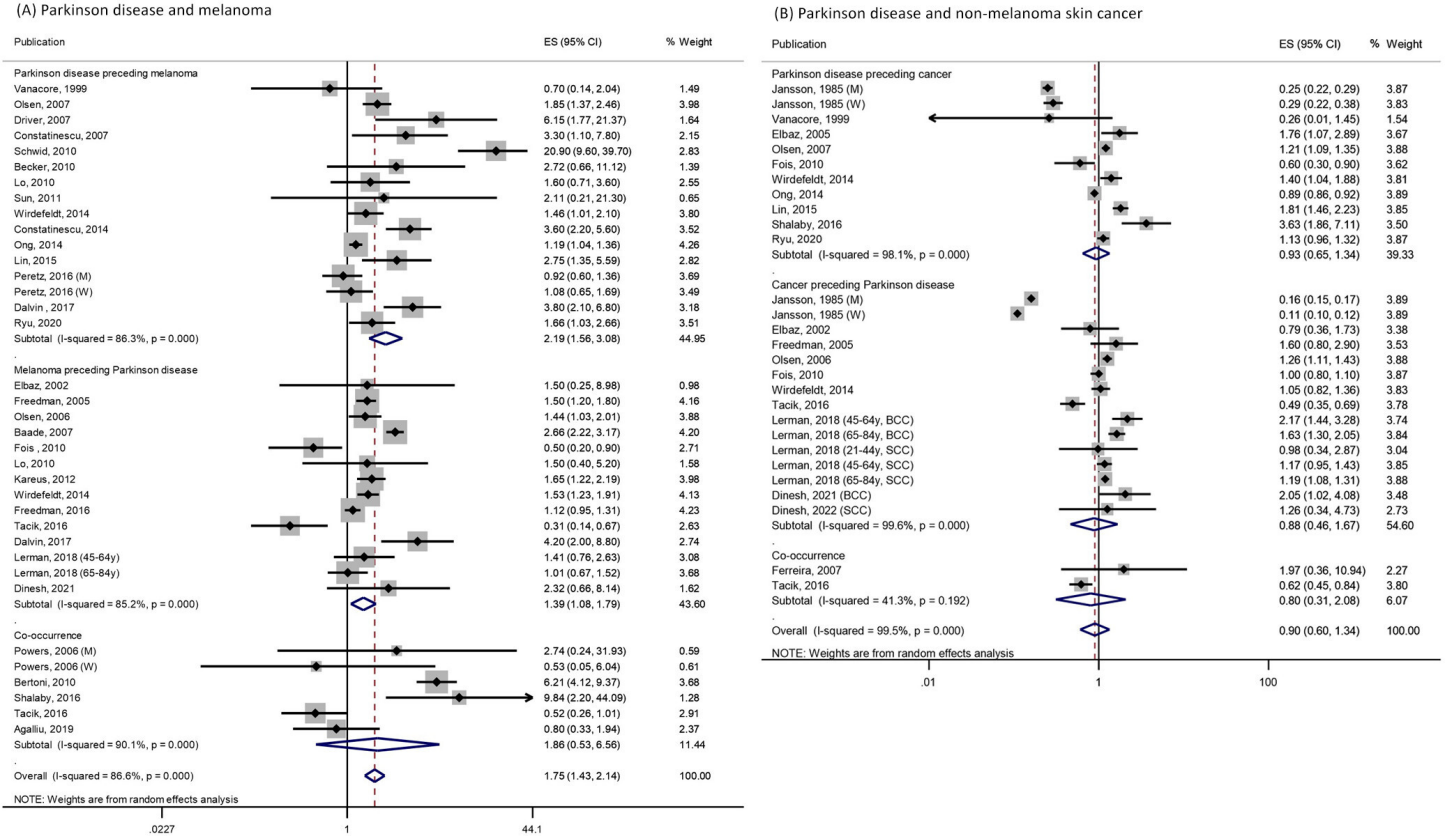
Association between Parkinson’s disease and (A) melanoma in 29 publications and (B) non-melanoma skin cancers in 17 publications. Figure shows the estimates (ESs) and 95% CIs for each study and the pooled result from random effects model. Studies are stratified by temporal relationship of Parkinson’s disease and cancer. BCC, basal cell carcinoma; M, men; SCC, squamous cell carcinoma; W, women; Y, years of age.

Combining 29 publications,^17 18 20 23 24 26 30 32 35 37 38 43 47 50 51 54 56 57 59 60 64 67–71 73 76 77^ the pooled RR for association between PD and melanoma was 1.75 (95% CI 1.43 to 2.14; p<0.001; figure 4A). No publication bias was observed (Egger test p=0.28; online supplemental figure 4a). We did not find a statistically significant association between PD and non-melanoma skin cancer (n=17; pooled RR=0.90; 95% CI 0.60 to 1.34; p=0.60; figure 4B).^31 32 34 35 37 41 47 50 54 56 57 67 69 71 73 76 77^ Egger test suggested no publication bias (p=0.53), but funnel plot suggested potential overestimation by small studies (online supplemental figure 4b).

### Other site-specific cancers

Lung cancer and colorectal cancer, two major cancers in the smoking-related category, both showed a significant inverse association with PD. There was no significant association between PD and breast cancer and prostate cancer (table 1).

## Discussion

In this meta-analysis of 63 publications and 17 994 584 participants, a significant inverse association between PD and total cancer was observed, with an 18% lower risk on both sides. Individuals with PD had a 15% lower risk of developing cancer, and vice versa, individuals with cancer had a 26% lower risk of developing PD. The inverse association was stronger for smoking-related cancers, compared with non-smoking-related cancers, though both achieved statistical significance. In contrast, PD was significantly associated with a 75% higher risk of melanoma. The overall inverse association is consistent with two published meta-analyses on this topic, which reported a 27% and 6% significantly lower risk for total cancer, respectively.^10 78^ Relative to these two published meta-analyses, our study included a large number of studies and participants. The latest meta-analysis, for example, included 15 studies and 1 480 239 participants for examining the association between PD and total cancer.^10 78^ In addition, this study did not stratify smoking-related and non-smoking-related cancers despite the analysis of associations between PD and specific cancers. Further, these two meta-analyses included both PD and parkinsonism.^10 78^

One of the possible explanations for the inverse association between PD and total cancer is smoking. Smoking has been consistently associated with a low risk of PD and a high risk of many types of cancer.^7^ Moreover, there is evidence that PD patients are less likely to be smokers.^79^ Of note, only 10 out of the 32 publications included were adjusted for smoking behaviour for total cancer risk in their original analysis,^18 25 27–31 51 64 75^ which may introduce residual confounding for the observed association between PD and total cancer. However, we also found that non-smoking-related cancer was inversely associated with PD, even when melanoma was included. Because only four publications separately reported risk estimates for total cancer or non-smoking-related cancers after excluding melanoma,^25 54 55 76^ we did not perform a meta-analysis in these secondary categories. Our findings suggest that smoking is unlikely the only factor contributing to the observed inverse relation between PD and total cancer. Future studies with carefully adjusted smoking habits or environmental smoking exposure are warranted to better address this issue.

Our results, in line with the previous meta-analysis,^10 78^ suggest an inverse comorbidity between PD and cancer. The biological bases underlying the association is far from clear. Dysregulated cellular processes including those involved in the regulation of cell cycle, mitochondrial function, DNA repair, cell metabolism and immune responses have been implicated in degeneration of neurons and tumourigenesis in dividing cells, often in the opposite directions. Cell proliferation and survival signals such as Wnt, P53, and PI3K/AKT may be upregulated in cancer and downregulated in neurodegeneration. The ubiquitin proteasome pathway of protein degradation on the other hand may be downregulated in neurodegeneration and upregulated in cancer.^80–82^ Understanding the biological pathways would further facilitate investigations on potential strategies for better prevention, surveillance and treatment of both PD and cancer.

Several common gene mutations have been implicated in PD and cancer.^83^ *PARK2* was found to be a potent tumour suppressor gene.^84 85^ Other PD-related genes *PINK1*, *PARK7* and *LRRK2* have also been linked to cancer.^8 60 86^ PD patients carrying *LRRK2* G2019S mutation have been associated with an overall increased risk of cancer, especially for hormone-related cancer and breast cancer,^84^ and most recently, lukaemia, colon cancer, and skin cancer when compared with noncarrier PD.^87^ Another PD-related *LRRK2* mutation R1441G was found to be associated with higher prevalence of haematological cancers.^88^ Both G2019S and R1441G show increased LRRK2 kinase activity.^89^ However, a recent study demonstrated that loss of *LRRK2* could promote lung cancer development, adding to the complexicity of LRRK2-cancer link.^90^ We found that only 14 of the included studies specifically identified idiopathic PD and excluded genetically determined PD. This limits our systematic review to distinguish genetic forms of PD from idiopathic PD and fully synthesise the potential genetic overlaps between PD and cancer.

Although similarly characterised pathologically by over proliferation, different cancers are highly heterogeneous. While it remains to be determined whether the general inverse association exists across cancers of different sites and evolutionary origins, we and others have consistently shown that it did not apply to melanoma.^9 91^ In this meta-analysis, we replicated the well-documented positive link between PD and melanoma. It has long been proposed that levodopa as the mainstay therapy for PD and a common precursor for both dopamine and melanin may contribute to the higher risk of melanoma in PD.^92 93^ In this meta-analysis, we found a 37% higher risk of newly developed PD after diagnosis of melanoma, suggesting that the observed PD-melanoma association may not be fully explained by the role of levodopa, if any.^94^ Previously, we reported that the risk of incident PD is higher in people with a family history of melanoma among their first-degree relatives.^91^ One plausible biological explanation of the association is the regulation of pigmentation by the *MC1R* gene, which presents and functions in both melanocytes and dopaminergic neurons.^95 96^ Other genetic mutations, such as *CYP2D6* polymorphism and *VDR* polymorphism, might also be involved in both conditions.^97–100^

Despite all our effort in synthesising all epidemiological evidence, the intrinsic limitations of meta-analysis cannot be avoided. First, studies included in this analysis came from diverse populations, with diverse designs and treatment strategies. They varied across assessments, statistical methods, and adjusted covariates. Although meta-regression did not find differences in age, sex, ethnicity, study design and study quality, the highly heterogeneous nature of this meta-analysis limits its interpretation into robust conclusions. Second, due to lack of access to original data, we could not adjust uniformly for confounders. We addressed this shortcoming by stratifying cancers into smoking-related or non-smoking-related cancers. However, there may be residual confounding since only a few studies adjusted for family history of PD/cancer, use of medications, sun exposure, duration of PD/cancer, use of medical care or diet (eg, caffeine consumption).^101–103^ Third, many large-scale studies included in this meta-analysis used local/national registry databases, with disease diagnosis mostly based on International Classification of Disease codes. Notification to registries might not be complete, therefore the cases might be under-reported. Moreover, diagnosis criteria may slightly vary in different countries, hospitals, etc. Thus it is challenging to confirm and validate the information from these datasets. Lastly, all publications included in this meta-analysis were based on populations from North America, Europe, Australia and Central and East Asia; No study has examined the association of PD and cancer in less-developed regions such as Africa, Southeast Asia or South America. This could be due to difficulties in disease diagnosis and registry in these regions. Recent findings suggested positive associations between PD and most cancers in an East Asian population, highlighting possible discrepancies among different populations with different ethnic backgrounds.^8 50^ Future studies should address the potentially important role of race/ethnicity and socialeconomic status.

We reviewed the current epidemiological evidence for the association between cancer and PD, with a meta-analysis of over 17 million individuals. We found that PD was associated with low risk of total cancer, except for melanoma, with which a positive association was identified. Despite the limitations, our study provided an overall picture of the association between the two major disease entities. Future studies should aim to better understand the links between these two major chronic disease entities using epidemiological, clinical and biological approaches.

## Supplementary Material

Reviewer comments

Author's manuscript

## Data Availability

All data relevant to the study are included in the article or uploaded as online supplemental information. The data used to support the findings of this article are included within the article and online supplemental material.

## Data availability statement

The data used to support the findings of this article are included within the article and the supplementary material.

## References

[1] Zafar S, Yaddanapudi SS. Parkinson disease. StatPearls. treasure island (fl): StatPearls publishing Copyright © 2021. StatPearls Publishing LLC, 2021.

[2] Mappin-Kasirer B, Pan H, Lewington S, et al. Tobacco smoking and the risk of Parkinson disease: a 65-year follow-up of 30,000 male British doctors. Neurology 2020;94:e2132–8. 10.1212/WNL.0000000000009437 32371450 PMC7526668

[3] Lesage S, Brice A. Parkinson's disease: from monogenic forms to genetic susceptibility factors. Hum Mol Genet 2009;18:R48–59. 10.1093/hmg/ddp012 19297401

[4] Ferlay J, Colombet M, Soerjomataram I, et al. Estimating the global cancer incidence and mortality in 2018: GLOBOCAN sources and methods. Int J Cancer 2019;144:1941–53. 10.1002/ijc.31937 30350310

[5] Ejma M, Madetko N, Brzecka A, et al. The Links between Parkinson’s Disease and Cancer. Biomedicines 2020;8:416. 10.3390/biomedicines8100416 PMC7602272 33066407

[6] Kendal WS. Dying with cancer: the influence of age, comorbidity, and cancer site. Cancer 2008;112:1354–62. 10.1002/cncr.23315 18286532

[7] Mappin-Kasirer B, Pan H, Lewington S, et al. Tobacco smoking and the risk of Parkinson disease. Neurology 2020;94:e2132–8. 10.1212/WNL.0000000000009437 32371450 PMC7526668

[8] Feng DD, Cai W, Chen X. The associations between Parkinson's disease and cancer: the plot thickens. Transl Neurodegener 2015;4:20. 10.1186/s40035-015-0043-z 26504519 PMC4620601

[9] Huang P, Yang X-D, Chen S-D, et al. The association between Parkinson's disease and melanoma: a systematic review and meta-analysis. Transl Neurodegener 2015;4:21. 10.1186/s40035-015-0044-y 26535116 PMC4631109

[10] Bajaj A, Driver JA, Schernhammer ES. Parkinson's disease and cancer risk: a systematic review and meta-analysis. Cancer Causes Control 2010;21:697–707. 10.1007/s10552-009-9497-6 20054708

[11] Walker E, Hernandez AV, Kattan MW. Meta-Analysis: its strengths and limitations. Cleve Clin J Med 2008;75:431–9. 10.3949/ccjm.75.6.431 18595551

[12] Ben-Shlomo Y, Marmot MG. Survival and cause of death in a cohort of patients with parkinsonism: possible clues to aetiology? J Neurol Neurosurg Psychiatry 1995;58:293–9. 10.1136/jnnp.58.3.293 7897409 PMC1073364

[13] Hely MA, Morris JG, Traficante R, et al. The Sydney multicentre study of Parkinson's disease: progression and mortality at 10 years. J Neurol Neurosurg Psychiatry 1999;67:300–7. 10.1136/jnnp.67.3.300 10449550 PMC1736543

[14] Higgins JPT, Thompson SG, Deeks JJ. Measuring inconsistency in meta-analyses. BMJ 2003;327:557–60. 10.1136/bmj.327.7414.557 12958120 PMC192859

[15] Wells GA, Shea B, Da O’Connell. The Newcastle-Ottawa scale (NOS) for assessing the quality of nonrandomised studies in meta-analyses. Oxford, 2000.

[16] Lushniak BD, Samet JM, Pechacek TF. The health consequences of smoking—50 years of progress: a report of the surgeon General; 2014.

[17] Baade PD, Fritschi L, Freedman DM. Mortality due to amyotrophic lateral sclerosis and Parkinson's disease among melanoma patients. Neuroepidemiology 2007;28:16–20. 10.1159/000097851 17164565

[18] Becker C, Brobert GP, Johansson S, et al. Cancer risk in association with Parkinson disease: a population-based study. Parkinsonism Relat Disord 2010;16:186–90. 10.1016/j.parkreldis.2009.11.005 19945903

[19] Bermejo-Pareja F, Benito-Leon J, Trincado R, et al. Neurodegenerative diseases (Alzheimer and Parkinson), but not vascular dementia, are negatively associated to cancer as cause of death in the NEDICES cohort (P01.086). Neurology 2012;78:86. 10.1212/WNL.78.1_MeetingAbstracts.P01.086

[20] Bertoni JM, Arlette JP, Fernandez HH, et al. Increased melanoma risk in Parkinson disease: a prospective clinicopathological study. Arch Neurol 2010;67:347–52. 10.1001/archneurol.2010.1 20212233

[21] Binaghi E, Mascia MM, Solla P. Correlation between Parkinson’s disease and malignant tumors. Movement Disorders 2016;31:S631–2.

[22] Boursi B, Mamtani R, Haynes K, et al. Parkinson's disease and colorectal cancer risk-A nested case control study. Cancer Epidemiol 2016;43:9–14. 10.1016/j.canep.2016.05.007 27232063 PMC4963291

[23] Constantinescu R, Elm J, Auinger P, et al. Malignant melanoma in early-treated Parkinson's disease: the NET-PD trial. Mov Disord 2014;29:263–5. 10.1002/mds.25734 24323565 PMC4375545

[24] Constantinescu R, Romer M, Kieburtz K, et al. Malignant melanoma in early Parkinson's disease: the DATATOP trial. Mov Disord 2007;22:720–2. 10.1002/mds.21273 17373726

[25] Cui X, Liew Z, Hansen J, et al. Cancers preceding Parkinson's disease after adjustment for bias in a Danish population-based case-control study. Neuroepidemiology 2019;52:136–43. 10.1159/000494292 30661072 PMC6482096

[26] Dalvin LA, Damento GM, Yawn BP, et al. Parkinson disease and melanoma: confirming and reexamining an association. Mayo Clin Proc 2017;92:1070–9. 10.1016/j.mayocp.2017.03.014 28688464 PMC5682925

[27] D'Amelio M, Ragonese P, Morgante L, et al. Tumor diagnosis preceding Parkinson's disease: a case-control study. Mov Disord 2004;19:807–11. 10.1002/mds.20123 15254939

[28] Driver JA, Kurth T, Buring JE, et al. Prospective case-control study of nonfatal cancer preceding the diagnosis of Parkinson's disease. Cancer Causes Control 2007;18:705–11. 10.1007/s10552-007-9005-9 17562193

[29] Driver JA, Kurth T, Buring JE, et al. Parkinson disease and risk of mortality: a prospective comorbidity-matched cohort study. Neurology 2008;70:1423–30. 10.1212/01.wnl.0000310414.85144.ee 18413567

[30] Driver JA, Logroscino G, Buring JE, et al. A prospective cohort study of cancer incidence following the diagnosis of Parkinson's disease. Cancer Epidemiol Biomarkers Prev 2007;16:1260–5. 10.1158/1055-9965.EPI-07-0038 17548694

[31] Elbaz A, Peterson BJ, Bower JH, et al. Risk of cancer after the diagnosis of Parkinson's disease: a historical cohort study. Mov Disord 2005;20:719–25. 10.1002/mds.20401 15704188

[32] Elbaz A, Peterson BJ, Yang P, et al. Nonfatal cancer preceding Parkinson's disease: a case-control study. Epidemiology 2002;13:157–64. 10.1097/00001648-200203000-00010 11880756

[33] Fall P-A, Saleh A, Fredrickson M, et al. Survival time, mortality, and cause of death in elderly patients with Parkinson's disease: a 9-year follow-up. Mov Disord 2003;18:1312–6. 10.1002/mds.10537 14639673

[34] Ferreira J, Silva JM, Freire R, et al. Skin cancers and precancerous lesions in Parkinson's disease patients. Mov Disord 2007;22:1471–5. 10.1002/mds.21575 17516496

[35] Fois AF, Wotton CJ, Yeates D, et al. Cancer in patients with motor neuron disease, multiple sclerosis and Parkinson's disease: record linkage studies. J Neurol Neurosurg Psychiatry 2010;81:215–21. 10.1136/jnnp.2009.175463 19726405

[36] Freedman DM, Pfeiffer RM. Associations between Parkinson disease and cancer in US Asian Americans. JAMA Oncol 2016;2:1093–4. 10.1001/jamaoncol.2016.0729 27196082

[37] Freedman DM, Travis LB, Gridley G, et al. Amyotrophic lateral sclerosis mortality in 1.9 million us cancer survivors. Neuroepidemiology 2005;25:176–80. 10.1159/000087447 16103728

[38] Freedman DM, Wu J, Chen H, et al. Associations between cancer and Parkinson's disease in U.S. elderly adults. Int J Epidemiol 2016;45:741–51. 10.1093/ije/dyw016 26989123 PMC5841885

[39] Gorell JM, Johnson CC, Rybicki BA. Parkinson's disease and its comorbid disorders: an analysis of Michigan mortality data, 1970 to 1990. Neurology 1994;44:1865–8. 10.1212/WNL.44.10.1865 7936238

[40] Jamrozik Z, Janik P, Friedman A. Incidence of prostate cancer in patients with Parkinson’s disease. Eur J Neurol 2005;12:98.

[41] Jansson B, Jankovic J. Low cancer rates among patients with Parkinson's disease. Ann Neurol 1985;17:505–9. 10.1002/ana.410170514 4004173

[42] Jespersen CG, Nørgaard M, Borre M. Parkinson's disease and risk of prostate cancer: a Danish population-based case-control study, 1995-2010. Cancer Epidemiol 2016;45:157–61. 10.1016/j.canep.2016.11.002 27838564

[43] Kareus SA, Figueroa KP, Cannon-Albright LA, et al. Shared predispositions of parkinsonism and cancer: a population-based pedigree-linked study. Arch Neurol 2012;69:1572–7. 10.1001/archneurol.2012.2261 22945795

[44] Kelm RC, KP, LS J. Melanoma and Parkinson disease: a large, urban, single-center, midwestern U.S. population study. J Am Acad Dermatol 2018;79. 10.1016/j.jaad.2018.05.765

[45] Lai S-W, Liao K-F, Lin C-H, et al. Parkinson's disease and lung cancer: a population-based case-control study in Taiwan. Geriatr Gerontol Int 2013;13:238–40. 10.1111/j.1447-0594.2012.00919.x 23286570

[46] Lai SW, Lin CL, Liao KF. Parkinson’s disease and hepatocellular carcinoma in older people: A population-based case-control study in Taiwan. International Medical Journal 2015;22:313–4.

[47] Lerman S, Amichai B, Weinstein G, et al. Parkinson's disease, melanoma, and keratinocyte carcinoma: a population-based study. Neuroepidemiology 2018;50:168–73. 10.1159/000487855 29566384

[48] Liao KF, Lin CL, Lai SW. Parkinson’s disease and risk of colorectal cancer: A population-based case-control study in Taiwan. Neurology Asia 2017;22:133–8.

[49] Liao KF, Lin CL, Lai SW. Parkinson’s disease and risk of pancreatic cancer: a population-based case-control study in Taiwan. Neurology Asia 2015;20:251–5.

[50] Lin P-Y, Chang S-N, Hsiao T-H, et al. Association between Parkinson disease and risk of cancer in Taiwan. JAMA Oncol 2015;1:633–40. 10.1001/jamaoncol.2015.1752 26181771

[51] Lo RY, Tanner CM, Van Den Eeden SK, et al. Comorbid cancer in Parkinson's disease. Mov Disord 2010;25:1809–17. 10.1002/mds.23246 20669266

[52] Minami Y, Yamamoto R, Nishikouri M, et al. Mortality and cancer incidence in patients with Parkinson's disease. J Neurol 2000;247:429–34. 10.1007/s004150070171 10929271

[53] Naghavi-Behzad M, Piri R. Risk of cancer among patients with Parkinson disease. Parkinsonism Relat Disord 2016;22:e34. 10.1016/j.parkreldis.2015.10.040

[54] Olsen JH, Friis S, Frederiksen K. Malignant melanoma and other types of cancer preceding Parkinson disease. Epidemiology 2006;17:582–7. 10.1097/01.ede.0000229445.90471.5e 16837822

[55] Olsen JH, Friis S, Frederiksen K, et al. Atypical cancer pattern in patients with Parkinson's disease. Br J Cancer 2005;92:201–5. 10.1038/sj.bjc.6602279 15583688 PMC2361753

[56] Olsen JH, Tangerud K, Wermuth L, et al. Treatment with levodopa and risk for malignant melanoma. Mov Disord 2007;22:1252–7. 10.1002/mds.21397 17534943

[57] Ong ELH, Goldacre R, Goldacre M. Differential risks of cancer types in people with Parkinson's disease: a national record-linkage study. Eur J Cancer 2014;50:2456–62. 10.1016/j.ejca.2014.06.018 25065294

[58] Park J-H, Kim D-H, Park Y-G, et al. Cancer risk in patients with Parkinson's disease in South Korea: a nationwide, population-based cohort study. Eur J Cancer 2019;117:5–13. 10.1016/j.ejca.2019.04.033 31229950

[59] Peretz C, Gurel R, Rozani V, et al. Cancer incidence among Parkinson's disease patients in a 10-yrs time-window around disease onset: a large-scale cohort study. Parkinsonism Relat Disord 2016;28:68–72. 10.1016/j.parkreldis.2016.04.028 27161827

[60] Agalliu I, Ortega RA, Luciano MS, et al. Cancer outcomes among Parkinson's disease patients with leucine rich repeat kinase 2 mutations, idiopathic Parkinson's disease patients, and nonaffected controls. Mov Disord 2019;34:1392–8. 10.1002/mds.27807 31348549 PMC6754269

[61] Ording AG, Veres K, Horváth-Puhó E, et al. Alzheimer's and Parkinson's diseases and the risk of cancer: a cohort study. J Alzheimers Dis 2019;72:1269–77. 10.3233/JAD-190867 31707368

[62] Pinter B, Diem-Zangerl A, Wenning GK, et al. Mortality in Parkinson's disease: a 38-year follow-up study. Mov Disord 2015;30:266–9. 10.1002/mds.26060 25447933

[63] Piri R, Naghavi-Behzad M. Association between cancers and risk of Parkinson disease: a cohort study. Parkinsonism Relat Disord 2016;22:e33. 10.1016/j.parkreldis.2015.10.039

[64] Powers KM, Smith-Weller T, Franklin GM, et al. Diabetes, smoking, and other medical conditions in relation to Parkinson's disease risk. Parkinsonism Relat Disord 2006;12:185–9. 10.1016/j.parkreldis.2005.09.004 16364673

[65] Pressley JC, Louis ED, Tang M-X, et al. The impact of comorbid disease and injuries on resource use and expenditures in parkinsonism. Neurology 2003;60:87–93. 10.1212/WNL.60.1.87 12525724

[66] Rugbjerg K, Friis S, Lassen CF, et al. Malignant melanoma, breast cancer and other cancers in patients with Parkinson's disease. Int J Cancer 2012;131:1904–11. 10.1002/ijc.27443 22278152 PMC3636769

[67] Ryu HJ, Park J-H, Choi M, et al. Parkinson's disease and skin cancer risk: a nationwide population-based cohort study in Korea. J Eur Acad Dermatol Venereol 2020;34:2775–80. 10.1111/jdv.16462 32289877

[68] Schwid SR, Bausch J, Oakes D, et al. Cancer incidence in a trial of an antiapoptotic agent for Parkinson's disease. Mov Disord 2010;25:1801–8. 10.1002/mds.23006 20669311

[69] Shalaby SY, Louis ED. Increased odds of melanoma: Parkinson's disease, essential tremor, dystonia versus controls. Neuroepidemiology 2016;46:128–36. 10.1159/000443794 26820576 PMC5473151

[70] Sun L-M, Liang J-A, Chang S-N, et al. Analysis of Parkinson's disease and subsequent cancer risk in Taiwan: a nationwide population-based cohort study. Neuroepidemiology 2011;37:114–9. 10.1159/000331489 21986194

[71] Tacik P, Curry S, Fujioka S, et al. Cancer in Parkinson's disease. Parkinsonism Relat Disord 2016;31:28–33. 10.1016/j.parkreldis.2016.06.014 27372241 PMC5048511

[72] Tang C-F, Lu M-K, Muo C-H, et al. Increased risk of brain tumor in patients with Parkinson's disease: a nationwide cohort study in Taiwan. Acta Neurol Scand 2016;134:148–53. 10.1111/ane.12524 26508469

[73] Vanacore N, Spila-Alegiani S, Raschetti R, et al. Mortality cancer risk in parkinsonian patients: a population-based study. Neurology 1999;52:395–8. 10.1212/WNL.52.2.395 9932965

[74] Wing K, Douglas I, Bhaskaran K. A Cohort Analysis of the Association between Parkinson’s Disease and Cancer Using the UK General Practice Research Database. Pharmacoepidemiology and Drug Safety 2012;21:442–3.22021031

[75] Winter AC, Rist PM, Buring JE, et al. Prospective comorbidity-matched study of Parkinson’s disease and risk of mortality among women. BMJ Open 2016;6:5. 10.1136/bmjopen-2016-011888 PMC5051400 27670518

[76] Wirdefeldt K, Weibull CE, Chen H, et al. Parkinson's disease and cancer: a register-based family study. Am J Epidemiol 2014;179:85–94. 10.1093/aje/kwt232 24142916 PMC3864714

[77] Dinesh D, Lee JS, Gao X, et al. Skin conditions in early Parkinson's disease. Parkinsonism Relat Disord 2021;84:40–6. 10.1016/j.parkreldis.2021.01.018 33549915 PMC8900714

[78] Zhang P, Liu B. Association between Parkinson's disease and risk of cancer: a PRISMA-compliant meta-analysis. ACS Chem Neurosci 2019;10:4430–9. 10.1021/acschemneuro.9b00498 31584793

[79] Larsson SC, Carter P, Kar S, et al. Smoking, alcohol consumption, and cancer: a Mendelian randomisation study in UK Biobank and international genetic consortia participants. PLoS Med 2020;17:e1003178. 10.1371/journal.pmed.1003178 32701947 PMC7377370

[80] Driver JA. Inverse association between cancer and neurodegenerative disease: review of the epidemiologic and biological evidence. Biogerontology 2014;15:547–57. 10.1007/s10522-014-9523-2 25113739

[81] Ibáñez K, Boullosa C, Tabarés-Seisdedos R, et al. Molecular evidence for the inverse comorbidity between central nervous system disorders and cancers detected by transcriptomic meta-analyses. PLoS Genet 2014;10:e1004173. 10.1371/journal.pgen.1004173 24586201 PMC3930576

[82] Klus P, Cirillo D, Botta Orfila T, et al. Neurodegeneration and cancer: where the disorder prevails. Sci Rep 2015;5:15390. 10.1038/srep15390 26493371 PMC4615981

[83] Dube U, Ibanez L, Budde JP, et al. Overlapping genetic architecture between Parkinson disease and melanoma. Acta Neuropathol 2020;139:347–64. 10.1007/s00401-019-02110-z 31845298 PMC7379325

[84] Gong Y, Zack TI, Morris LGT, et al. Pan-Cancer genetic analysis identifies PARK2 as a master regulator of G1/S cyclins. Nat Genet 2014;46:588–94. 10.1038/ng.2981 24793136 PMC4251771

[85] Gupta A, Anjomani-Virmouni S, Koundouros N, et al. Park2 depletion connects energy and oxidative stress to PI3K/Akt activation via PTEN S-nitrosylation. Mol Cell 2017;65:999–1013. 10.1016/j.molcel.2017.02.019 28306514 PMC5426642

[86] Agalliu I, San Luciano M, Mirelman A, et al. Higher frequency of certain cancers in LRRK2 G2019S mutation carriers with Parkinson disease: a pooled analysis. JAMA Neurol 2015;72:58–65. 10.1001/jamaneurol.2014.1973 25401981 PMC4366130

[87] Shu L, Zhang Y, Pan H, et al. Clinical Heterogeneity Among LRRK2 Variants in Parkinson’s Disease: A Meta-Analysis. Front Aging Neurosci 2018;10. 10.3389/fnagi.2018.00283 PMC6156433 30283330

[88] Ruiz-Martínez J, de la Riva P, Rodríguez-Oroz MC, et al. Prevalence of cancer in Parkinson's disease related to R1441G and G2019S mutations in LRRK2. Mov Disord 2014;29:750–5. 10.1002/mds.25778 24357540

[89] Ysselstein D, Nguyen M, Young TJ, et al. LRRK2 kinase activity regulates lysosomal glucocerebrosidase in neurons derived from Parkinson's disease patients. Nat Commun 2019;10:5570. 10.1038/s41467-019-13413-w 31804465 PMC6895201

[90] Lebovitz C, Wretham N, Osooly M, et al. Loss of Parkinson's susceptibility gene LRRK2 promotes carcinogen-induced lung tumorigenesis. Sci Rep 2021;11:2097. 10.1038/s41598-021-81639-0 33483550 PMC7822882

[91] Gao X, Simon KC, Han J, et al. Family history of melanoma and Parkinson disease risk. Neurology 2009;73:1286–91. 10.1212/WNL.0b013e3181bd13a1 19841380 PMC2764417

[92] Weissenrieder JS, Neighbors JD, Mailman RB, et al. Cancer and the Dopamine D_2_ Receptor: A Pharmacological Perspective. J Pharmacol Exp Ther 2019;370:111–26. 10.1124/jpet.119.256818 31000578 PMC6558950

[93] Siple JF, Schneider DC, Wanlass WA, et al. Levodopa therapy and the risk of malignant melanoma. Ann Pharmacother 2000;34:382–5. 10.1345/aph.19150 10917387

[94] Bose A, Petsko GA, Eliezer D. And melanoma: co-occurrence and mechanisms. J Parkinsons Dis 2018;8:385–98. 29991141 10.3233/JPD-171263 PMC6130416

[95] Chen X, Feng D, Schwarzschild MA, et al. Red hair, MC1R variants, and risk for Parkinson's disease - a meta-analysis. Ann Clin Transl Neurol 2017;4:212–6. 10.1002/acn3.381 28275654 PMC5338132

[96] Gao X, Simon KC, Han J, et al. Genetic determinants of hair color and Parkinson's disease risk. Ann Neurol 2009;65:76–82. 10.1002/ana.21535 19194882 PMC2639631

[97] Fullard ME, Duda JE. A review of the relationship between vitamin D and Parkinson disease symptoms. Front Neurol 2020;11:454–54. 10.3389/fneur.2020.00454 32536905 PMC7267215

[98] Campbell MJ, Trump DL. Vitamin D receptor signaling and cancer. Endocrinol Metab Clin North Am 2017;46:1009–38. 29080633 10.1016/j.ecl.2017.07.007 PMC7092369

[99] Chan CWH, Law BMH, So WKW, et al. Pharmacogenomics of breast cancer: highlighting CYP2D6 and tamoxifen. J Cancer Res Clin Oncol 2020;146:1395–404. 10.1007/s00432-020-03206-w 32270286 PMC11804387

[100] Ur Rasheed MS, Mishra AK, Singh MP. Cytochrome P450 2D6 and Parkinson's disease: polymorphism, metabolic role, risk and protection. Neurochem Res 2017;42:3353–61. 10.1007/s11064-017-2384-8 28871472

[101] Schrempf M, Haluza D, Simic S, et al. Is multidirectional UV exposure responsible for increasing melanoma prevalence with altitude? A hypothesis based on calculations with a 3D-Human exposure model. Int J Environ Res Public Health 2016;13:961. 10.3390/ijerph13100961 PMC5086700 27690069

[102] Gross A, Racette BA, Camacho-Soto A, et al. Use of medical care biases associations between Parkinson disease and other medical conditions. Neurology 2018;90:e2155–65. 10.1212/WNL.0000000000005678 29743207 PMC5996836

[103] Mahajan A, Chirra M, Dwivedi AK, et al. Skin cancer may delay onset but not progression of Parkinson's disease: a nested case-control study. Front Neurol 2020;11:406. 10.3389/fneur.2020.00406 32547471 PMC7270344

