# Supplementary material for: Parkinson’s disease and cancer: a systematic review and meta-analysis of over 17 million participants
Source: BMJ Open. 2021 Jul 2;11(7):e046329. doi: 10.1136/bmjopen-2020-046329 (PMC8256737; doi:10.1136/bmjopen-2020-046329)
Supplement: Supplementary data [file bmjopen-2020-046329supp001.pdf]

Supplementary material for "Parkinson Disease and cancer: a systematic review and meta-analysis of 17,697,252 participants"

Content

Supplementary methods

Search strategy

Duplicate database inclusion/exclusion

Supplementary tables

Table 1. Characteristics of publications included in meta-analysis of Parkinson Disease and cancer.

Table 2. Meta regression and sub-group analysis on association between Parkinson disease and total cancer.

Table 3. Publications on risk of total cancer associated with levodopa treatment.

Supplementary figures

Figure 1. Funnel plot of studies of the association between Parkinson Disease and total cancer.

Figure 2. Association between use of levodopa and risk of total cancer in 4 publications.

Figure 3. Funnel plot of studies of the association between Parkinson Disease and a) smoking-related cancers, b) non-smoking-related cancers.

Figure 4. Funnel plot of studies of the association between Parkinson Disease and a) melanoma, b) non-melanoma skin cancers.

Zhang X, *et al. BMJ Open* 2021; 11:e046329. doi: 10.1136/bmjopen-2020-046329

Supplementary methods

PubMed search strategy for Parkinson Disease and cancer

(("Parkinson Disease"[Mesh] OR "Parkinson Disease"[TW] OR "Parkinson's Disease"[TW] OR "Parkinsonism"[TW]) AND "cancer"[sb] AND ("Epidemiologic Studies"[Mesh] OR "Epidemiologic"[TW] OR "epidemiological"[TW] OR "Case-Control Studies"[Mesh] OR "case-control"[TW] OR "case control"[TW] OR "Case-Comparison"[TW] OR "Case Comparison"[TW] OR "Case-Compeer"[TW] OR "Case-Referent"[TW] OR "Case Referent"[TW] OR "Case-Base"[TW] OR "Case Base"[TW] OR "Cohort Studies"[Mesh] OR "cohort"[TW] OR "Concurrent"[TW] OR "Incidence"[TW] OR "Cross-Sectional Studies"[Mesh] OR "cross-sectional"[TW] OR "cross sectional"[TW] OR "Disease Frequency"[TW] OR "Prevalence"[TW] OR "Follow-Up Studies"[Mesh] OR "Follow-Up"[TW] OR "Follow Up"[TW] OR "Followup"[TW] OR "Longitudinal Studies"[Mesh] OR "longitudinal"[TW] OR "Retrospective Studies"[Mesh] OR "retrospective"[TW] OR "Prospective Studies"[Mesh] OR "prospective"[TW] OR "observational"[TW] OR "Observational Study" [Publication Type] OR "mortality studies"[TW] OR "ratio"[TW] OR "risk"[TW]) AND English[lang]) NOT ("animals"[MeSH Terms] NOT ("humans"[MeSH Terms] AND "animals"[MeSH Terms]))

Selection of publications that used same population

Leibson 2006, Elbaz 2002, Elbaz 2005, and Dalvin 2017 all used data from Mayo Clinic, Minnesota. Leibson 2006 was updated by Elbaz 2002 and 2005, therefore excluded from this meta-analysis. Elbaz 2002 studied PD risk after cancer, while Elbaz 2005 studied cancer risk after PD, therefore both publications were included. Dalvin 2017 was a cross-sectional extension of previous result, but it contained detailed analysis on melanoma, therefore it was not included in the analysis for total cancer, but was included for melanoma.

Olsen 2005, Olsen 2006, Olsen 2007, Rugbjerg 2012, Frandson 2014, Jespersen 2016, Cui 2019, and Ording 2019 all used National Hospital Register of Denmark. Frandson 2014 was a cross-sectional study that overlapped with Olsen 2006, Rugbjerg 2012, and Ording 2019, therefore was not included in this meta-analysis. Other publications varied in designs, time windows, temporal relationship, and cancer of interest.

Zhang X, *et al. BMJ Open* 2021; 11:e046329. doi: 10.1136/bmjopen-2020-046329

There were also multiple publications from Physicians' Health Study, Women's Health Study, and Taiwan Health Registry. However, all of these groups of paper varied in designs, time windows, temporal relationship, and cancer of interest, therefore were not considered as duplicates.

Other duplicates were meeting proceedings/abstracts of later-published articles, or duplicates that were not identified by matching in Endnote X9.

Supplementary tables

Table 1. Characteristics of publications included in meta-analysis of Parkinson Disease (PD) and cancer.

Author	Year	Publication type	Temporal direction	Study design	Location	Cohort	Case N	Control N	Follow time, y	Disease ascertainment	Smoking adjusted	Quality score
Agalliu	2019	Article	Co-occurrence	Cross-sectional	Europe, Israel, and the United States	Michael J. Fox Foundation	712	218	\	Diagnosed; idiopathic PD	No	5
Baade	2007	Article	Cancer preceding PD	Case-only cohort	Australia		127037		6.0	Coded	No	5
Becker	2010	Article	PD preceding cancer	1. Matched cohort 2. Case-control	UK	UK-based General Practice Research Database	466	1864	\	Validated; idiopathic PD	Yes	9
Ben-Shlomo	1995	Article	PD preceding cancer	Matched cohort	England and Wales	Second National Morbidity Study	220	421	\	Coded	No	7
Bermejo-Pareja	2012	Abstract	PD preceding cancer	Prospective cohort	Spain	Neurologic Disorders in Central Spain (NEDICES)	81	5197	\	\	No	\
Bertoni	2010	Article	Co-occurence	Case-only cohort	North America		2106		\	Diagnosed	No	7
Binagh	2016	Abstract	Co-occurence	Cross-sectional	Italy		529		\	Diagnosed	Yes	\
Boursi	2016	Article	PD preceding cancer	Case-control	UK	The Health Improvement Network	22093	85833	\	Diagnosed	Yes	9
Constatinescu	2007	Article	PD preceding cancer	Case-only cohort	North America	DATATOP	800		4.61	Diagnosed; idiopathic PD	No	4
Constatinescu	2014	Article	PD preceding cancer	Case-only cohort	US	NET-PD	1737		3.71	Diagnosed; idiopathic PD	No	4

Zhang X, *et al. BMJ Open* 2021; 11:e046329. doi: 10.1136/bmjopen-2020-046329

**Author**	**Year**	**Publication type**	**Temporal direction**	**Study design**	**Location**	**Cohort**	**Case N**	**Control N**	**Follow time, y**	**Disease ascertainment**	**Smoking adjusted**	**Quality score**
Cui	2019	Article	Cancer preceding PD	Case-control	Denmark	National Hospital Register	1813	1887	\	Diagnosed; idiopathic PD	Yes	9
Dalvin	2017	Article	Both	1. Case-control 2. Matched cohort	Minnesota	Mayo clinic	974	2922	5	Coded	No	7
D'Amellio	2004	Article	Cancer preceding PD	Case-control	Italy		222	222	\	Diagnosed; idiopathic PD	Yes	8
Dinesh	2021	Article	Cancer preceding PD	Case-control	US	PPMI database	423	196	\	Diagnosed	No	8
Driver	2007	Article	PD preceding cancer	Matched cohort	US	Physicians' Health Study	487	487	5.2	Validated; idiopathic PD	Yes	8
Driver	2007	Article	Cancer preceding PD	Case-control	US	Physicians' Health Study	487	487	\	Validated; idiopathic PD	Yes	8
Driver	2008	Article	PD preceding cancer	Matched cohort	US	Physicians' Health Study	560	560	5.8	Validated; idiopathic PD	Yes	8
Elbaz	2002	Article	Cancer preceding PD	Case-control	Minnesota	Mayo clinic	196	196	5.5	Diagnosed	No	7
Fall	2003	Article	PD preceding cancer	Matched cohort	Sweden		170	510	4.8	Diagnosed	No	8
Elbaz	2005	Article	PD preceding cancer	Matched cohort	Minnesota	Mayo clinic	196	185	8	Diagnosed	Yes	6
Ferreira	2007	Article	Co-occurence	Cross-sectional	Portugal	The Lisbon University Hospital	150	146	\	Diagnosed; idiopathic PD	No	5
Fois	2010	Article	Both	Case-only cohort	UK	Oxford Record Linkage Study	4355		3.2	Coded	No	7

Author	Year	Publication type	Temporal direction	Study design	Location	Cohort	Case N	Control N	Follow time, y	Disease ascertainment	Smoking adjusted	Quality score
Freedman	2005	Article	Cancer preceding PD	Case-only cohort	US	SEER-Medicare	1900000		8.5	Coded	No	6
Freedman	2016	Letter	PD preceding cancer	Case-control	US (Asian Americans)	SEER-Medicare	20627	5558	\	Coded	No	7
Freedman	2016	Article	Cancer preceding PD	1. Case-control 2. cohort	US	SEER-Medicare	743779	419432	2.8	Coded	No	7
Gorell	1994	Article	Co-occurence	Cross-sectional	Michigan		8629	208933	\	Coded	No	7
Hely	1999	Article	PD preceding cancer	Case-only cohort	Australia	Sydney Multicenter Study of PD	130		9.1	Diagnosed	No	5
Jansson	1985	Article	Both	Prospective cohort	US		406		8.6	Diagnosed	Yes	\
Jamrozik	2005	Abstract	Cancer preceding PD	Case-control	Poland		100	100	\	\	No	7
Jespersen	2016	Article	Co-occurence	Case-control	Denmark	National Registry	45429	227145	\	Coded	No	7
Kareus	2012	Article	Cancer preceding PD	Case-control	US	Utah Cancer Registry	2300000			Coded	No	6
Kelm	2018	Abstract	Co-occurence	Case-control	US	Northwestern Medicine Enterprise Data Warehouse medical record	4751	9494	5.75	Coded	No	\
Lai	2013	Letter	Co-occurence	Case-control	Taiwan	National Health	2822	11288	\	Coded	Yes	8
Lai	2015	Article	Co-occurence	Case-control	Taiwan	National Health	1815	7260	\	Coded	No	8

Zhang X, *et al. BMJ Open* 2021; 11:e046329. doi: 10.1136/bmjopen-2020-046329

Author	Year	Publication type	Temporal direction	Study design	Location	Cohort	Case N	Control N	Follow time, y	Disease ascertainment	Smoking adjusted	Quality score
Lerman	2018	Article	PD preceding cancer	Prospective cohort	Isreal	Maccabi Health Services	7727	1243968	\	Coded	Yes	9
Liao	2015	Article	PD preceding cancer	Case-control	Taiwan	National Health	13861	55444	\	Coded	No	8
Liao	2017	Article	PD preceding cancer	Case-control	Taiwan	National Health	64619	64619	\	Coded	No	8
Lin	2015	Article	PD preceding cancer	Matched cohort	Taiwan	National Health	62023	124046	\	Coded	No	6
Lo	2010	Article	Both	Matched cohort	US	PEAK	692	761	5.0; 4.3	Diagnosed	Yes	7
Minami	2000	Article	PD preceding cancer	Case-only cohort	Japan		228		6.97	Validated	No	6
Naghavi-Behzad	2016	Abstract	PD preceding cancer	Case-control	Iran		\		\	\	No	\
Olsen	2005	Denmark	PD preceding cancer	National Hospital Register			14088		5.0	Coded; idiopathic PD	No	8
Olsen	2006	Article	Cancer preceding PD	Case-control	Denmark	National Hospital Register	8090	32320	\	Coded; idiopathic PD	No	6
Olsen	2007	Article	PD preceding cancer	Case-only cohort	Denmark	National Hospital Register	14088		\	Coded; idiopathic PD	No	6
Ong	2014	Article	PD preceding cancer	Prospective cohort	UK	NHS hospital	219194	9015614	\	Coded	No	8
Ording	2019	Article	PD preceding cancer	Case-only cohort	Denmark	National Hospital Register	28835		4.0	Coded	No	7
Park	2019	Article	PD preceding cancer	Matched cohort	South Korea	NHI	52009	260045	\	Coded	No	8

Zhang X, *et al. BMJ Open* 2021; 11:e046329. doi: 10.1136/bmjopen-2020-046329

Author	Year	Publication type	Temporal direction	Study design	Location	Cohort	Case N	Control N	Follow time, y	Disease ascertainment	Smoking adjusted	Quality score
Peretz	2016	Article	PD preceding cancer	Case-only cohort	Israel	Maccabi Health Services	7125		10.5	Validated	No	6
Pinter	2015	Article	PD preceding cancer	Case-only cohort	Austria		237		14.8	Coded	No	6
Piri	2016	Abstract	PD preceding cancer	Prospective cohort		Cancer Registry Database	2584		\	Diagnosed	No	\
Powers	2006	Article	Co-occurence	Case-control	Seattle		352	484	\	Diagnosed; idiopathic PD	Yes	8
Pressley	2003	Article	Co-occurence	Cross-sectional	US	National Long-Term Care Survey	791	24040	\	Coded	No	6
Rugbjerg	2012	Article	PD preceding cancer	Case-only cohort	Denmark	National Hospital Register	20343		5.7	Coded	No	6
Ryu	2020	Article	PD preceding cancer	Matched cohort	Korea	South Korea National Health Insurance System	70780	353900	8	Diagnosed	No	7
Schwid	2010	Article	PD preceding cancer	Case-only cohort	US	PRECEPT	806		1.8	Diagnosed/verified	No	4
Shalaby	2016	Article	Co-occurence	Case-control	US	Columbia University Medical Cente	108	124	\	Self-report	No	6
Sun	2011	Article	PD preceding cancer	Matched cohort	Taiwan	NHI	4957	19828	\	Coded	No	8
Tacik	2016	Article	1. Co-occurrence 2. cancer preceding PD	Prospective cohort	Florida	Mayo clinic	971	478	4.6	Diagnosed	No	6

Author	Year	Publication type	Temporal direction	Study design	Location	Cohort	Case N	Control N	Follow time, y	Disease ascertainment	Smoking adjusted	Quality score
Tang	2016	Article	PD preceding cancer	Matched cohort	Taiwan	NHI	2998	11992	\	Coded	No	7
Vanacore	1999	Communication	PD preceding cancer	Case-only cohort	Italy		10322		5.7	Drug	No	4
Wing	2012	Abstract	Both	Prospective cohort	UK		8549	42160	\	\	Yes	\
Winter	2016	Article	PD preceding cancer	Matched cohort	US	Women's Health Study	396	396	6.2	Self-report	Yes	7
Wirdefeldt	2014	Article	Both	Matched cohort	Sweden		11786	58930	\	Coded	No	6

Study design and temporal direction was defined per each individual study definition, most of which was based on the diagnosis date of two diseases. Disease ascertainment was defined per the description of whether any physicians, neurologists or movement specialists made the diagnosis. Quality score was assessed by the Newcastle-Ottawa Scale for cohort studies and for case-control studies (range 0–9).

Zhang X, *et al. BMJ Open* 2021; 11:e046329. doi: 10.1136/bmjopen-2020-046329

Table 2. Subgroup-analysis of the association between Parkinson disease and cancer.

	No. of publications	Pooled RR (95% CI)	P for significance	P for heterogeneity	P difference
**Age**					**0.10**
< 69.3 years	13	0.70 (0.42, 1.19)	0.21	<0.001	
≥ 69.3 years	14	0.90 (0.81, 1.00)	0.05	<0.001	
**Sex**					**0.31**
Men-dominant	23	0.76 (0.57, 1.02)	0.07	<0.001	
Women-dominant	12	0.91 (0.70, 1.17)	0.45	<0.001	
**Ethnicity**					**0.19**
Caucasian-dominant	27	0.75 (0.59, 0.96)	0.02	<0.001	
Asian-dominant	6	0.98 (0.75, 1.28)	0.88	<0.001	
**Study design**					**0.92**
Prospective cohort	24	0.79 (0.65, 0.96)	0.05	<0.001	
Other	9	0.79 (0.65, 0.96)	0.02	<0.001	
**Newcastle-Ottawa quality score**					**0.31**
≤ 6	12	0.87 (0.71, 1.08)	0.21	<0.001	
≥ 7	21	0.75 (0.57, 0.98)	0.04	<0.001	
**Period of study**					**0.19**
< 2010	16	0.73 (0.61, 0.88)	0.001	<0.001	
≥ 2010	17	0.88 (0.80, 0.96)	0.003	<0.001	

6 publications did not report mean/median age or age range.

2 publications did not report sex ratio. 4 publications separately report risk estimates for men and women, therefore counted in both sex groups.

Zhang X, *et al. BMJ Open* 2021; 11:e046329. doi: 10.1136/bmjopen-2020-046329

Table 3. Publications on risk of total cancer associated with levodopa treatment.

**Publication**	**Estimation (95% confidence interval)**	**Note**
Elbaz, 2005	1.26 (0.39, 4.12)	4th (>1,313 g) compared to 1st quartile of cumulative levodopa
Constanescu, 2007	1.4 (0.3, 4.3)	After levodopa use
Olsen, 2007	1.0 (0.5, 2.0)	≥1370 g compared to 600-1369 g of cumulative levodopa
Becker, 2010	0.7 (0.56, 0.88)	≥5 prescription of levodopa

Zhang X, *et al. BMJ Open* 2021; 11:e046329. doi: 10.1136/bmjopen-2020-046329

Supplementary figures

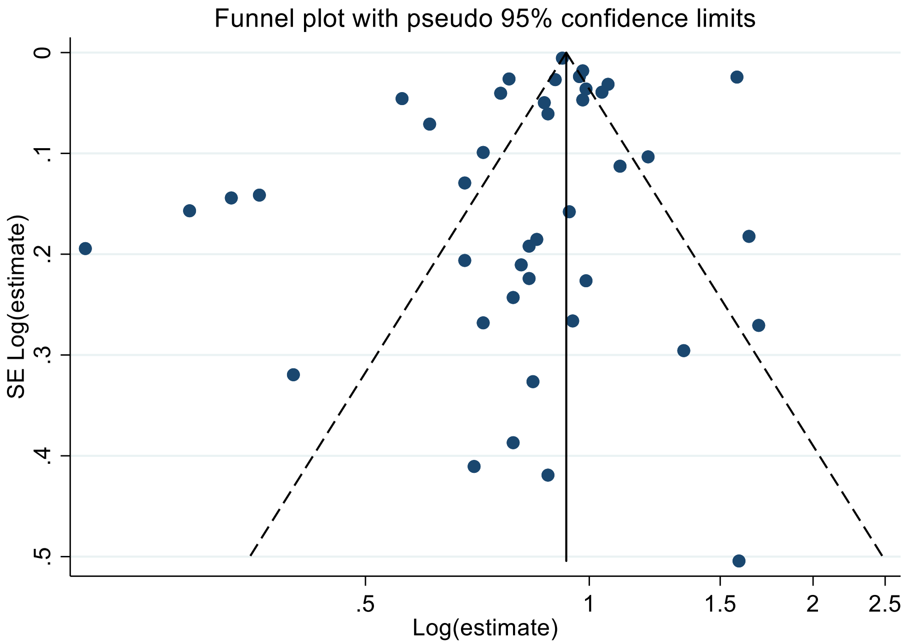

Figure 1. Funnel plot of studies of the association between Parkinson Disease and total cancer. The log-transformed risk estimates from each study is plotted on the horizontal axis, and its standard error is plotted on the vertical axis.

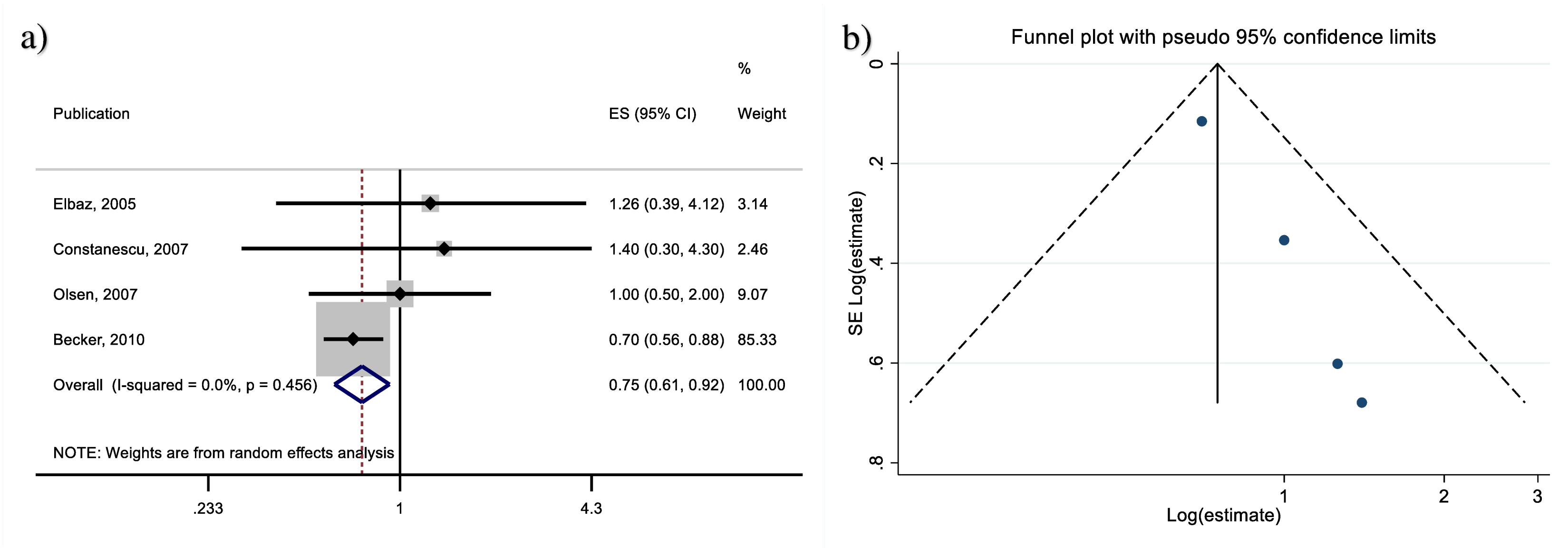

Figure 2. a) Individual and pooled estimates of the association between use of levodopa and risk of total cancer in 4 publications. Figure shows the estimates (ESs) and 95% confidence intervals (CIs) for each individual studies and the pooled result from random effects model. b) Funnel plots of these 4 publications.

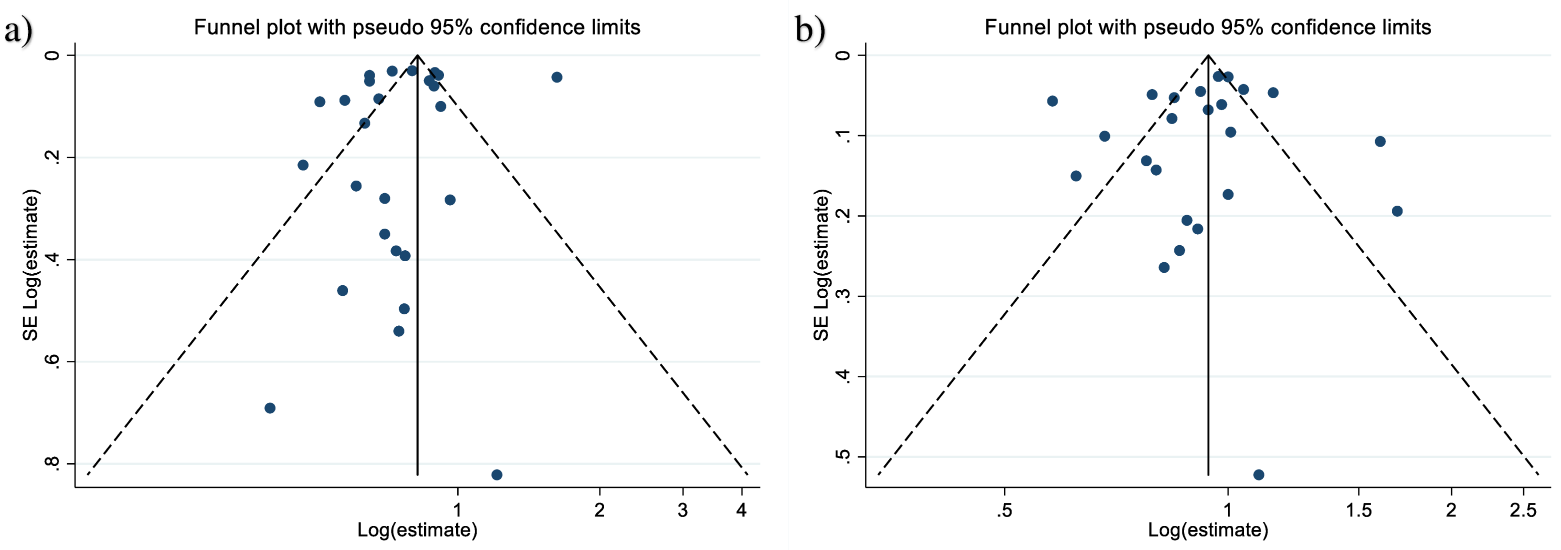

Figure 3. Funnel plot of studies of the association between Parkinson Disease and a) smoking-related cancers, b) non-smoking-related cancers. The log-transformed risk estimates from each study is plotted on the horizontal axis, and its standard error is plotted on the vertical axis.

Zhang X, *et al. BMJ Open* 2021; 11:e046329. doi: 10.1136/bmjopen-2020-046329

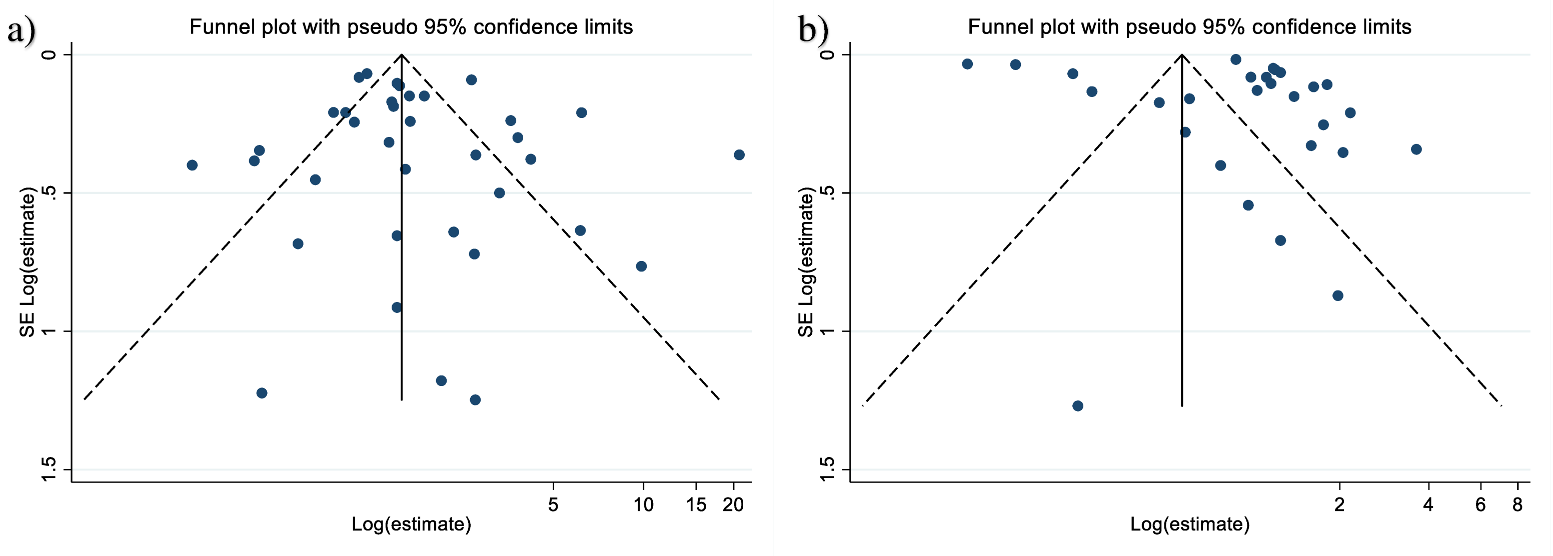

Figure 4. Funnel plot of studies of the association between Parkinson Disease and a) melanoma, b) non-melanoma skin cancers. The log-transformed risk estimates from each study is plotted on the horizontal axis, and its standard error is plotted on the vertical axis.
