# Supplementary material for: Parkinson’s disease and cancer: a systematic review and meta-analysis of over 17 million participants
Source: BMJ Open. 2021 Jul 2;11(7):e046329. doi: 10.1136/bmjopen-2020-046329 (PMC8256737; doi:10.1136/bmjopen-2020-046329)
Supplement: Author's manuscript [file bmjopen-2020-046329.draft_revisions.pdf]

BMJ Open is committed to open peer review. As part of this commitment we make the peer review history of every article we publish publicly available.

When an article is published we post the peer reviewers' comments and the authors' responses online. We also post the versions of the paper that were used during peer review. These are the versions that the peer review comments apply to.

The versions of the paper that follow are the versions that were submitted during the peer review process. They are not the versions of record or the final published versions. They should not be cited or distributed as the published version of this manuscript.

BMJ Open is an open access journal and the full, final, typeset and author-corrected version of record of the manuscript is available on our site with no access controls, subscription charges or pay-per-view fees (http://bmjopen.bmj.com).

If you have any questions on BMJ Open's open peer review process please email info.bmjopen@bmj.com

BMJ Open

Parkinson's disease and cancer: a systematic review and meta-analysis of 17,697,252 participants

Journal:	*BMJ Open*
Manuscript ID	bmjopen-2020-046329
Article Type:	Original research
Date Submitted by the Author:	28-Oct-2020
Complete List of Authors:	Zhang, Xinyaun; The Pennsylvania State University, Nutritional Science<br>Guarin, David; Massachusetts General Hospital, Neurology<br>Chen, Xiqun; Massachusetts General Hospital, Neurology<br>Gao, Xiang; The Pennsylvania State University, Nutritional Science
Keywords:	ONCOLOGY, EPIDEMIOLOGY, Parkinson-s disease < NEUROLOGY, Epidemiology < ONCOLOGY

SCHOLARONE™
Manuscripts

I, the Submitting Author has the right to grant and does grant on behalf of all authors of the Work (as defined in the below author licence), an exclusive licence and/or a non-exclusive licence for contributions from authors who are: i) UK Crown employees; ii) where BMJ has agreed a CC-BY licence shall apply, and/or iii) in accordance with the terms applicable for US Federal Government officers or employees acting as part of their official duties; on a worldwide, perpetual, irrevocable, royalty-free basis to BMJ Publishing Group Ltd ("BMJ") its licensees and where the relevant Journal is co-owned by BMJ to the co-owners of the Journal, to publish the Work in this journal and any other BMJ products and to exploit all rights, as set out in our licence.

The Submitting Author accepts and understands that any supply made under these terms is made by BMJ to the Submitting Author unless you are acting as an employee on behalf of your employer or a postgraduate student of an affiliated institution which is paying any applicable article publishing charge ("APC") for Open Access articles. Where the Submitting Author wishes to make the Work available on an Open Access basis (and intends to pay the relevant APC), the terms of reuse of such Open Access shall be governed by a Creative Commons licence – details of these licences and which Creative Commons licence will apply to this Work are set out in our licence referred to above.

Other than as permitted in any relevant BMJ Author's Self Archiving Policies, I confirm this Work has not been accepted for publication elsewhere, is not being considered for publication elsewhere and does not duplicate material already published. I confirm all authors consent to publication of this Work and authorise the granting of this licence.

For peer review only

Parkinson's disease and cancer: a systematic review and meta-analysis of 17,697,252 participants

Xinyuan Zhang, BSc[1#], David Guarin[2#], Xiqun Chen, MD, PhD[1*], Xiang Gao, MD, PhD[2*]

[1]Department of Nutritional Sciences, The Pennsylvania State University, University Park, PA 16802, USA

[2]Department of Neurology, Massachusetts General Hospital, Harvard Medical School, Boston, MA 02114, USA

#Co-first author; *Co-senior author

Correspondence should be addressed to Xiqun Chen**;** xchen17@mgh.harvard.edu

Word Count: 2690

Keywords: Neoplasms; Epidemiology; Meta-analysis; Parkinson's disease; Odds ratio.

For peer review only

For peer review only - http://bmjopen.bmj.com/site/about/guidelines.xhtml

Abstract

Objective

To systematically review and qualitatively evaluate all epidemiological evidence on associations between PD and cancer via meta-analysis.

Data Sources

MEDLINE via PubMed, Web of Science, and EMBASE, until March 2020.

Study Selection

Included were publications that: 1) were original epidemiological studies on PD and cancer; 2) reported risk estimates; 3) were in English. Exclusion criteria included: 1) review/comments; 2) biological studies; 3) case report/autopsy studies; 4) irrelevant exposure/outcome; 5) treated cases; 6) no measure of risk estimates; 7) no confidence intervals/exact p values; and 8) duplicates.

Data extraction and Synthesis

PRISMA and MOOSE guidelines were followed in data extraction. Two-step screening was performed by two authors blinded to each other. A random effects model was used to calculate pooled relative risk (RR).

Main Outcomes and Measures

We included publications that assessed risk of PD in individuals with vs without cancer, and risk of cancer in individuals with vs without PD.

Results

For peer review only

For peer review only - http://bmjopen.bmj.com/site/about/guidelines.xhtml

A total of 61 studies and 17,697,252 participants were included. Meta-analysis generated a pooled relative risk of 0.82 (n = 33; 95% CI: 0.76, 0.88; p <0.001) for association between PD and total cancer, 0.76 (n = 21; 95% CI: 0.67, 0.85; p <0.001) for PD and smoking-related cancer, and 0.92 (n = 19; 95% CI: 0.84, 0.99; p = 0.03) for non-smoking-related cancer. PD was associated with an increased risk of melanoma (n = 27; pooled relative risk = 1.75; 95% CI: 1.42, 2.15; p <0.001) but not for other skin cancers (n=15; pooled relative risk = 0.85; 95%CI: 0.56, 1.30; p = 0.46).

Conclusions

PD and total cancer were inversely associated. This inverse association persisted for both smoking-related and non-smoking-related cancers. In contrast, PD was positively associated with melanoma.

For peer review only

Article Summary

Strengths and limitations of this study

- Unlike recent metanalysis, this study stratifies analysis for smoking vs non-smoking cancers.
- Heterogeneity between included studies was analyzed via meta regression.
- Despite best efforts, high heterogeneity in methodology and cohorts of included studies cannot be fully dealt with by statistical methods.

For peer review only

For peer review only - http://bmjopen.bmj.com/site/about/guidelines.xhtml

Introduction

Parkinson's disease (PD) is a neurodegenerative disease characterized by premature cell death of dopaminergic neurons in the substantia nigra pars compacta (SNpc) [1]. Cancer is characterized by uncontrolled cell proliferation and growth [2]. Growing epidemiological evidence suggests that PD and cancer may be inversely associated [3]. However, it remains unclear whether PD and cancer is associated mechanistically, or the findings were confounded by other factors, such as study designs, and smoking, which is associated with both PD and certain cancers. In contrast to the overall inverse association between PD and cancer, there is a positive, bidirectional association between PD and melanoma, a malignant tumor that develops from melanocytes [4]. A higher risk of PD in melanoma patients, and vice versa, has been proposed to be due to some shared metabolic pathways, such as the melanocortin 1 receptor (MC1R) pigmentation pathway [5]. Clearly documenting these associations is important for bridging the interdisciplinary knowledge gap and developing novel preventive and treatment strategies for both PD and cancer. PD and cancers are both relatively rare diseases [6,7]. An individual study may lack power to detect an association. A meta-analysis can increase precision in estimating risk [8], especially in subsets of cancers with even fewer cases. We thus conducted a meta-analysis to systematically review the population-based evidence for the potential association between PD and cancer. To better elucidate PD-cancer relation, we first stratified studies according to the temporal association between the two diseases into three categories: PD preceding cancer, cancer preceding PD, and co-occurrence. Secondly, we performed sensitivity analysis in which variations in study design and qualities, and levodopa treatment, were evaluated. Thirdly, we separately analyzed smoking-related cancers and non-smoking-related cancers to address smoking as a potential confounding factor. Finally, we

For peer review only - http://bmjopen.bmj.com/site/about/guidelines.xhtml

specifically analyzed the associations between PD and melanoma, non-melanoma skin cancers, and other major cancers (eg, prostate cancer, colon cancer and breast cancer).

For peer review only

For peer review only - http://bmjopen.bmj.com/site/about/guidelines.xhtml

Methods

Literature search and data extraction

This meta-analysis followed the MOOSE guidelines for reporting meta-analysis on observational studies, and was registered on PROSPERO (CRD42020162103). We searched all published literature that reported PD association with cancer in MEDLINE via PubMed, Web of Science, and EMBASE up to March 1, 2020. Search items related to "Parkinson's disease", "cancer", and "epidemiologic studies" were identified and modified for each database. We constrained our search in human studies and in English language. Detailed search terms can be found in Supplementary materials. Duplicates were matched based on author, year, and title in Endnote X9 and manually compared before removing.

The inclusion criteria were: 1) original studies that was conducted in an epidemiological setting; 2) studies reported either an odds ratio (OR), risk ratio (RR), hazard ratio (HR), standardized incidence/mortality ratio (SIR/SMR), or other reliable measure of estimated risk; 3) studies in which PD and cancer cases were ascertained by doctor's diagnosis, hospitalization record, disease identification codes, or self-report on diagnosis. Parkinsonism was not included. Both benign and malignant neoplasms were included. Exclusion criteria included: 1) reviews or comments; 2) non-epidemiological studies; 3) case reports/autopsy studies; 4) irrelevant exposure/outcome; 5) treated cases; 6) no measure of risk estimates; 7) no confidence intervals/exact p values; and 8) duplicates. Two first authors (X. Z. and D. G.) independently screened the publications in two steps: title/abstract screening and full text screening. Any discrepancy was reviewed and reconciled by two senior authors (X. C. and X. G.). During full text screening, we found 5 groups of publications using a same population or dataset. Details of inclusion and exclusion step are reported in

For peer review only - http://bmjopen.bmj.com/site/about/guidelines.xhtml

Supplementary methods. After screening references of included publications, we found two other eligible publications that were not captured by search items [9,10].

Data extraction

From each of the included publications, we extracted information on first author, year of the study, study type, country origin, population, mean age, dominant sex, dominant ethnicity, cases and controls population size, measure of risk, PD and cancer ascertainment methods, adjusted covariates, levodopa use, and estimated risk with lower and upper confidence intervals (CIs) for each type of cancer.

Type of study was categorized into prospective study, case-control study, case-only cohort study, and cross-sectional study.

Statistical analysis

All analyses were performed in STATA SE 15. Cochran's Q statistic and I-squared were calculated to examine heterogeneity among studies. Cochran's Q was computed as sum of variance from the pooled estimates and compared to chi-squared distribution with k-1 (k = number of publications) degree of freedom. I-squared was calculated as the percentage of variation across studies due to heterogeneity rather than chance [11]. Due to high heterogeneity of included publications (p-value for Q statistics <0.05, I-squared >50% for all), pooled effect sizes (including RR, OR, HR, SIR and SMR) were calculated using random-effects models to account for unobserved heterogeneity. Egger test and funnel plots were performed to assess publication bias.

For total cancer, we performed three 1 analyses. First, 4 publications from meeting precedings/abstracts were further included; second, 8 mortality publications were excluded; third, 2 publications using invalidated, self-report diagnosis of either cancer or PD were excluded.

For peer review only - http://bmjopen.bmj.com/site/about/guidelines.xhtml

Further, we performed six subgroup analyses, looking at variance of the included publications in population age, dominant sex, dominant race/ethnicity, study design, study quality, and year of study. Age was separated into two groups by the mean age of the included studies (69.3 years). Dominant ethnicity was categorized into Caucasian-dominant and Asian-dominant. Study design was categorized into cohort studies and other type of studies. Study quality was assessed by Newcastle-Ottawa Scale for cohort studies and for case-control studies [12], and separated into low quality group (< 7) and high quality group (≥ 7), based on the mean quality score of the included studies . The difference between groups was tested by meta regression method.

We categorized cancers into smoking-related and non-smoking-related cancers according to National Cancer Institute and Centers for Disease Control and Prevention's definition [13]. Smoking-related cancers include cancer of the lung, larynx, mouth, esophagus, throat, bladder, kidney, liver, stomach, pancreas, colon and rectum, and cervix, as well as acute myeloid leukemia. Cancers of other sites, including melanoma, were regarded as not associated with smoking. If a publication reported grouped smoking- and non-smoking-related cancers, the risk estimates were extracted directly. If a publication reported individual cancers only, and the number of sites is more than 10, we first categorized individual cancers into smoking-related and non-smoking-related groups accordingly [13], calculated pooled RR and 95% CI in each group using random-effects model, and then included the resulting pooled RR in the final meta-analysis.

We specifically evaluated the association between PD and melanoma, and other skin cancers. Cancers of other specific sites were included in this meta-analysis if there were more than 10 publications. Included were lung cancer, colorectal cancer, breast cancer, and prostate cancer.

For peer review only - http://bmjopen.bmj.com/site/about/guidelines.xhtml

Results

In total, we included 61 publications in this meta-analysis (Figure 1) [9,10,14-72]. Characteristics of all publications are listed in Supplementary table 1.

PD and total cancer

Combining 33 publications [9,10,15,24-29,32-36,38,47-49,51,54-57,59,60,63-65,67,69,70,72], pooled RR for association between PD and cancer was 0.82 (95% CI: 0.76, 0.88; p <0.001; Figure 2). We did not observe evidence for existence of publication bias (Egger test p = 0.27; supplementary figure 1). After stratified by temporal sequence, PD was significantly associated with a lower future risk of cancer (n = 21, pooled RR = 0.85; 95% CI: 0.76, 0.95; p = 0.004), and similar association was observed for cancer with a lower future risk of PD (n = 11, pooled RR = 0.74; 95% CI: 0.65, 0.85; p = <0.001). The significant inverse association persisted after further including meeting abstracts, excluding mortality studies, and excluding self-report outcomes that were not validated (table 1). Meta regression did not find significant difference between subgroups stratified by age (<69.3 years vs ≥69.3 years; mean value of the included studies), sex (men- vs women-dominant cohorts), ethnicity (Caucasian vs Asian), study design (cohort vs others), study quality (scored <7 vs ≥ 7), or year of study (before 2010, or 2010 and after, supplementary table 2).

We found 4 publications that examined risk of cancers associated with treatment of levodopa in PD patients (supplementary table 3) [15,21,28,53]. Although there was a significant lower risk of cancer after levodopa treatment or with higher cumulative levodopa treatment (pooled RR = 0.75; 95% CI: 0.61, 0.92; p = 0.007; supplementary figure 2a), Egger test (p = 0.005) and funnel plot

For peer review only - http://bmjopen.bmj.com/site/about/guidelines.xhtml

(supplementary figure 2b) showed a significant publication bias and thus a potentially over-estimated result.

Smoking- and non-smoking-related cancers

Combining 21 publications [10,15,25,27-29,32,34,47,48,51,54-56,61,64,65,67,70,72], the pooled RR for association between PD and smoking-related cancers was 0.76 (95% CI: 0.67, 0.85; p <0.001; figure 3a). PD was also inversely associated with non-smoking-related cancers (n = 19; pooled RR = 0.92; 95% CI: 0.84, 0.99; p = 0.03; figure 3b) [10,15,22,25,27,28,32,34,35,47,48,54-56,61,64,65,67,70]. No publication bias was observed for both analyses (Egger test p = 0.45 and 0.50, respectively; supplementary figure 3).

Melanoma and non-melanoma skin cancer

Combining 27 publications [14,15,17,20,21,23,27,29,32,34,35,40,44,47,48,51,53,54,56,59,62-65,67,70,71], the pooled RR for association between PD and melanoma was 1.75 (95% CI: 1.42, 2.15; p <0.001; figure 4a). No publication bias was observed (Egger test p = 0.31; supplementary figure 4a). We did not find a statistically significant association between PD and non-melanoma skin cancer (n = 15; pooled RR = 0.85; 95% CI: 0.56, 1.30; p = 0.46; figure 4b) [28,29,31,32,34,38,44,47,51,53,54,63,65,67,70]. Egger test suggested no publication bias (p = 0.62), but funnel plot suggested potential over-estimation by small studies (supplementary figure 4b).

Other site-specific cancers

Lung cancer and colorectal cancer, two major cancers in the smoking-related category, both showed significant inverse association with PD. There was no significant association between PD and breast cancer and prostate cancer (table 1).

For peer review only - http://bmjopen.bmj.com/site/about/guidelines.xhtml

Discussion

In this meta-analysis of 61 publications and 17,697,252 participants, a significant inverse association between PD and total cancer was observed, with an 18% lower risk on both sides. Individuals with PD had 15% lower risk of developing cancer, and vice versa, individuals with cancer had 26% lower risk of developing PD. The inverse association was stronger for smoking-related cancers, compared to non-smoking-related cancers, though both achieved statistical significance. In contrast, PD was significantly associated with 74% higher risk of melanoma.

The overall inverse association is consistent with two published meta-analysis on this topic, which reported a 27% and 6% significantly lower risk for total cancer, respectively [3,73]. Relative to these two published meta-analyses, our study included a large number of studies and participants. The latest meta-analysis, for example, included 15 studies and 1,480,239 participants for examining the association between PD and total cancer [3,73]. In addition, this study did not stratify smoking-related and non-smoking-related cancers despite the analysis of associations between PD and specific cancers. Further, these two meta-analyses included both PD and Parkinsonism [3,73].

One of the possible explanations for the inverse association between PD and total cancer is smoking. Smoking has been consistently associated with a low risk of PD [74] and a high risk of many types of cancer [13]. Moreover, there is evidence that PD patients are less likely to be smokers [75]. Of note, only 10 out of the 32 publications included were adjusted for smoking behavior for total cancer risk in their original analysis [15,22,24-28,48,59,69], which may introduce residual confounding for the observed association between PD and total cancer. However, we also found that non-smoking-related cancer was inversely associated with PD, even

For peer review only - http://bmjopen.bmj.com/site/about/guidelines.xhtml

when melanoma was included. Because only 4 publications separately reported risk estimate for total cancer or non-smoking-related cancers after excluding melanoma [22,51,52,70], we did not perform a meta-analysis in these secondary categories. This suggests that smoking is unlikely the only factor contributing to the observed inverse relation between PD and total cancer. Future studies with carefully adjusted smoking habits or environmental smoking exposure are warranted to better address this question.

Our results, in line with the previous meta-analysis [3], suggest that PD and cancer patients may be protected against each other. It remains to be elucidated though whether the inverse comorbidity has biological bases. Several common gene mutations have been implicated in PD and cancer. PARK2 was found to be a potent tumor suppressor gene [76,77]. Other PD-related genes PINK-1, DJ-1, and more recently LRRK2 have also been linked to cancer [71,78,79]. Embryonic mutation of the oncogene BRAF caused neurodegeneration [80]. These common genetic defects contribute to degeneration of neurons and tumorigenesis in dividing cells via altered cellular processes including those involved in the regulation of cell cycle, mitochondrial function, DNA repair, cell metabolism, and immune response, often in the opposite directions [81]. Indeed, cell proliferation and survival signals such as Wnt, P53, and PI3K/AKT may be upregulated in cancer and downregulated in neurodegeneration. The ubiquitin proteasome pathway of protein degradation on the other hand may be downregulated in neurodegeneration and upregulated in cancer [82-84]. Understanding the biological pathways would further facilitate investigations on potential strategies for better prevention, surveillance, and treatment of both PD and cancer.

Although similarly characterized pathologically by over proliferation, different cancers are highly heterogeneous. While it remains to be determined whether the general inverse association exists

For peer review only - http://bmjopen.bmj.com/site/about/guidelines.xhtml

across cancers of different sites and evolutionary origins, we and others have consistently shown that it did not apply to melanoma [4,85]. In this meta-analysis, we replicated the well-documented positive link between PD and melanoma. It has long been proposed that levodopa as the mainstay therapy for PD and common precursor for both dopamine and melanin may contribute to the higher risk of melanoma in PD [86]. In this meta-analysis, we found a 37% higher risk of newly-developed PD after diagnosis of melanoma, suggesting that the observed PD-melanoma association may not be fully explained by the role of levodopa, if any [87]. Previously, we reported that risk of incident PD is higher in people with a family history of melanoma among their first-degree relatives [85]. One plausible biological explanation of the association is the regulation of pigmentation by *MC1R* gene, which presents and functions in both melanocytes and dopaminergic neurons [55,88]. Other genetic mutations, such as CYP2D6 polymorphism and VDR polymorphism, might also be involved in both conditions [89-91].

Despite all our effort in synthesizing all epidemiological evidence, the intrinsic limitations of meta-analysis cannot be avoided. First, studies included in this analysis came from diverse populations, with diverse designs and treatment strategies. They varied across assessments, statistical methods, and adjusted covariates. Although meta-regression did not find difference in age, sex, ethnicity, study design and study quality, the highly heterogeneous nature of this meta-analysis limits its interpretation into robust conclusions. Second, due to lack of access to original data, we could not adjust uniformly for confounders. We addressed this shortcoming by stratifying cancers into smoking-related or non-smoking-related cancers. However, there may be residual confounding since only few studies adjusted for family history of PD/cancer, use of medications, duration of PD/cancer, use of medical care [92], or diet (eg, caffeine consumption). Third, many large-scale studies included in this meta-analysis used local/national registry databases, with disease diagnosis

For peer review only - http://bmjopen.bmj.com/site/about/guidelines.xhtml

For peer review only

mostly based on International Classification of Disease codes. Notification to registries might not be complete, therefore the cases might be under-reported. Moreover, diagnosis criteria may slightly vary in different countries, hospitals, etc. Thus it is challenging to confirm and validate the information from these datasets. Lastly, all publications included in this meta-analysis were based on populations from North America, Europe, Australia, and Central and East Asia; No study has examined association of PD and cancer in less-developed regions such as Africa, Southeast Asia or South America. This could be due to difficulties in disease diagnosis and registry in these regions. Recent findings suggested positive associations between PD and most cancers in an East Asian population, highlighting possible discrepancy among different populations with different ethnic backgrounds [47,79]. Future studies should address the potentially important role of race/ethnicity and social-economic status.

We reviewed the current epidemiological evidence for the association between cancer and PD, with a meta-analysis of 17,697,252 participants. We found that PD was associated with low risk of total cancer, except for melanoma, with which a positive association was identified. Despite the limitations, our study provided an overall picture of the association between the two major disease entities. Future studies should aim to better understand the links between these two major chronic disease entities using epidemiological, clinical, and biological approaches.

For peer review only - http://bmjopen.bmj.com/site/about/guidelines.xhtml

Funding: This work was supported by The National Institutes of Health [R01NS102735], The Parkinson's Disease Foundation [PF-APDA-SFW-1914], The Jane & Alan Batkin Foundation [N/A], and The Farmer Family Foundation [N/A]. None of the listed funders had a role in the design and conduct of the study; collection, management, analysis, and interpretation of the data; preparation, review, or approval of the manuscript; and decision to submit the manuscript for publication.

Data Availability: The data used to support the findings of this article are included within the article and the supplementary material.

Patient and Public Involvement Statement: It was not appropriate to involve patients or the public in the design, or conduct, or reporting, or dissemination plans of our research.

Competing Interests: The authors declare no conflicts of interest.

Acknowledgements: We thank Christina Wissinger, PhD, from Health Sciences Liaison Librarian of Penn State, who helped with search strategy. No financial compensation was made.

Author Contributions

Name	Location	Contribution
Xinyuan Zhang, BSc	The Pennsylvania State University	Concept and design; Acquisition, analysis, or interpretation of data; Statistical analysis; Drafting of the manuscript; revised the manuscript for intellectual content

For peer review only

David Guarin	Massachusetts General Hospital	Concept and design; Acquisition, analysis, or interpretation of data; revised the manuscript for intellectual content
Xiqun Chen, MD, PhD	Massachusetts General Hospital	Concept and design; Acquisition, analysis, or interpretation of data; Drafting of the manuscript; revised the manuscript for intellectual content
Xiang Gao, MD, PhD	The Pennsylvania State University	Concept and design; Acquisition, analysis, or interpretation of data; Drafting of the manuscript; revised the manuscript for intellectual content

For peer review only

References

[1] Forno LS. Neuropathology of Parkinson's disease. J Neuropathol Exp Neurol 1996;55:259-272.

[2] Harris TJ, McCormick F. The molecular pathology of cancer. Nat Rev Clin Oncol 2010;7:251-265.

[3] Bajaj A, Driver JA, Schernhammer ES. Parkinson's disease and cancer risk: a systematic review and meta-analysis. Cancer Causes Control 2010;21:697-707.

[4] Huang P, Yang XD, Chen SD, Xiao Q. The association between Parkinson's disease and melanoma: a systematic review and meta-analysis. Transl Neurodegener 2015;4:21.

[5] Chen X, Feng D, Schwarzschild MA, Gao X. Red hair, MC1R variants, and risk for Parkinson's disease - a meta-analysis. Ann Clin Transl Neurol 2017;4:212-216.

[6] Dorsey ER, Elbaz A, Nichols E, et al. Global, regional, and national burden of Parkinson's disease, 1990–2016: a systematic analysis for the Global Burden of Disease Study 2016. The Lancet Neurology 2018;17:939-953.

[7] Global Burden of Disease Cancer C, Fitzmaurice C, Akinyemiju TF, et al. Global, Regional, and National Cancer Incidence, Mortality, Years of Life Lost, Years Lived With Disability, and Disability-Adjusted Life-Years for 29 Cancer Groups, 1990 to 2016: A Systematic Analysis for the Global Burden of Disease Study. JAMA Oncol 2018;4:1553-1568.

[8] Walker E, Hernandez AV, Kattan MW. Meta-analysis: Its strengths and limitations. Cleve Clin J Med 2008;75:431-439.

[9] Hely MA, Morris JG, Traficante R, Reid WG, O'Sullivan DJ, Williamson PM. The sydney multicentre study of Parkinson's disease: progression and mortality at 10 years. Journal of neurology, neurosurgery, and psychiatry 1999;67:300-307.

[10] Ben-Shlomo Y, Marmot MG. Survival and cause of death in a cohort of patients with parkinsonism: possible clues to aetiology? Journal of neurology, neurosurgery, and psychiatry 1995;58:293-299.

[11] Higgins JP, Thompson SG, Deeks JJ, Altman DG. Measuring inconsistency in meta-analyses. BMJ 2003;327:557-560.

[12] Peterson J, Welch V, Losos M, Tugwell P. The Newcastle-Ottawa scale (NOS) for assessing the quality of nonrandomised studies in meta-analyses.

[13] National Center for Chronic Disease P, Health Promotion Office on S, Health. Reports of the Surgeon General. The Health Consequences of Smoking-50 Years of Progress: A Report of the Surgeon General. Atlanta (GA): Centers for Disease Control and Prevention (US), 2014.

[14] Baade PD, Fritschi L, Freedman DM. Mortality due to amyotrophic lateral sclerosis and Parkinson's disease among melanoma patients. Neuroepidemiology 2007;28:16-20.

[15] Becker C, Brobert GP, Johansson S, Jick SS, Meier CR. Cancer risk in association with Parkinson disease: a population-based study. Parkinsonism Relat Disord 2010;16:186-190.

For peer review only - http://bmjopen.bmj.com/site/about/guidelines.xhtml

[16] Bermejo-Pareja F, Benito-Leon J, Trincado R, et al. Neurodegenerative Diseases (Alzheimer and Parkinson), but Not Vascular Dementia, Are Negatively Associated to Cancer as Cause of Death in the NEDICES Cohort (P01.086). Neurology 2012;78:P01.086-P001.086.

[17] Bertoni JM, Arlette JP, Fernandez HH, et al. Increased melanoma risk in Parkinson disease: a prospective clinicopathological study. Arch Neurol 2010;67:347-352.

[18] Binaghi E, Mascia MM, Solla P, et al. Correlation between Parkinson's disease and malignant tumors. Mov Disord 2016;31:S631-S632.

[19] Boursi B, Mamtani R, Haynes K, Yang YX. Parkinson's disease and colorectal cancer risk-A nested case control study. Cancer Epidemiol 2016;43:9-14.

[20] Constantinescu R, Elm J, Auinger P, et al. Malignant melanoma in early-treated Parkinson's disease: the NET-PD trial. Mov Disord 2014;29:263-265.

[21] Constantinescu R, Romer M, Kieburtz K, Group DIotPS. Malignant melanoma in early Parkinson's disease: the DATATOP trial. Mov Disord 2007;22:720-722.

[22] Cui X, Liew Z, Hansen J, Lee PC, Arah OA, Ritz B. Cancers Preceding Parkinson's Disease after Adjustment for Bias in a Danish Population-Based Case-Control Study. Neuroepidemiology 2019;52:136-143.

[23] Dalvin LA, Damento GM, Yawn BP, Abbott BA, Hodge DO, Pulido JS. Parkinson Disease and Melanoma: Confirming and Reexamining an Association. Mayo Clin Proc 2017;92:1070-1079.

[24] D'Amelio M, Ragonese P, Morgante L, et al. Tumor diagnosis preceding Parkinson's disease: a case-control study. Mov Disord 2004;19:807-811.

[25] Driver JA, Kurth T, Buring JE, Gaziano JM, Logroscino G. Prospective case-control study of nonfatal cancer preceding the diagnosis of Parkinson's disease. Cancer Causes Control 2007;18:705-711.

[26] Driver JA, Kurth T, Buring JE, Gaziano JM, Logroscino G. Parkinson disease and risk of mortality: a prospective comorbidity-matched cohort study. Neurology 2008;70:1423-1430.

[27] Driver JA, Logroscino G, Buring JE, Gaziano JM, Kurth T. A prospective cohort study of cancer incidence following the diagnosis of Parkinson's disease. Cancer epidemiology, biomarkers & prevention : a publication of the American Association for Cancer Research, cosponsored by the American Society of Preventive Oncology 2007;16:1260-1265.

[28] Elbaz A, Peterson BJ, Bower JH, et al. Risk of cancer after the diagnosis of Parkinson's disease: a historical cohort study. Mov Disord 2005;20:719-725.

[29] Elbaz A, Peterson BJ, Yang P, et al. Nonfatal cancer preceding Parkinson's disease: a case-control study. Epidemiology 2002;13:157-164.

[30] Fall PA, Saleh A, Fredrickson M, Olsson JE, Granerus AK. Survival time, mortality, and cause of death in elderly patients with Parkinson's disease: a 9-year follow-up. Mov Disord 2003;18:1312-1316.

[31] Ferreira J, Silva JM, Freire R, et al. Skin cancers and precancerous lesions in Parkinson's disease patients. Mov Disord 2007;22:1471-1475.

For peer review only - http://bmjopen.bmj.com/site/about/guidelines.xhtml

[32] Fois AF, Wotton CJ, Yeates D, Turner MR, Goldacre MJ. Cancer in patients with motor neuron disease, multiple sclerosis and Parkinson's disease: record linkage studies. Journal of neurology, neurosurgery, and psychiatry 2010;81:215-221.

[33] Freedman DM, Pfeiffer RM. Associations Between Parkinson Disease and Cancer in US Asian Americans. JAMA Oncol 2016;2:1093-1094.

[34] Freedman DM, Travis LB, Gridley G, Kuncl RW. Amyotrophic lateral sclerosis mortality in 1.9 million US cancer survivors. Neuroepidemiology 2005;25:176-180.

[35] Freedman DM, Wu J, Chen H, et al. Associations between cancer and Parkinson's disease in U.S. elderly adults. Int J Epidemiol 2016;45:741-751.

[36] Gorell JM, Johnson CC, Rybicki BA. Parkinson's disease and its comorbid disorders: an analysis of Michigan mortality data, 1970 to 1990. Neurology 1994;44:1865-1868.

[37] Jamrozik Z, Janik P, Friedman A, Sawicki J, Kwiecinski H. Incidence of prostate cancer in patients with Parkinson's disease. Eur J Neurol 2005;12:98-98.

[38] Jansson B, Jankovic J. Low cancer rates among patients with Parkinson's disease. Ann Neurol 1985;17:505-509.

[39] Jespersen CG, Norgaard M, Borre M. Parkinson's disease and risk of prostate cancer: A Danish population-based case-control study, 1995-2010. Cancer Epidemiol 2016;45:157-161.

[40] Kareus SA, Figueroa KP, Cannon-Albright LA, Pulst SM. Shared predispositions of parkinsonism and cancer: a population-based pedigree-linked study. Arch Neurol 2012;69:1572-1577.

[41] Kelm RC, K. P, J. LS, E. LA, P. WD, B. N. Melanoma and Parkinson disease: A large, urban, single-center, midwestern U.S. population study. J Am Acad Dermatol 2018;79.

[42] Lai SW, Liao KF, Lin CH, Tsai PY, Sung FC. Parkinson's disease and lung cancer: a population-based case-control study in Taiwan. Geriatr Gerontol Int 2013;13:238-240.

[43] Lai SW, Lin CL, Liao KF, Lin CM. Parkinson's disease and hepatocellular carcinoma in older people: A population-based case-control study in Taiwan. International Medical Journal 2015;22:313-314

[44] Lerman S, Amichai B, Weinstein G, Shalev V, Chodick G. Parkinson's Disease, Melanoma, and Keratinocyte Carcinoma: A Population-Based Study. Neuroepidemiology 2018;50:168-173.

[45] Liao KF, Lin CL, Lai SW. Parkinson's disease and risk of colorectal cancer: A population-based case-control study in Taiwan. Neurol Asia 2017;22:133-138.

[46] Liao KF, Lin CL, Lai SW, Chen WC. Parkinson's disease and risk of pancreatic cancer: a population-based case-control study in Taiwan. Neurol Asia 2015;20:251-255.

[47] Lin PY, Chang SN, Hsiao TH, Huang BT, Lin CH, Yang PC. Association Between Parkinson Disease and Risk of Cancer in Taiwan. JAMA Oncol 2015;1:633-640.

[48] Lo RY, Tanner CM, Van Den Eeden SK, Albers KB, Leimpeter AD, Nelson LM. Comorbid cancer in Parkinson's disease. Mov Disord 2010;25:1809-1817.

For peer review only - http://bmjopen.bmj.com/site/about/guidelines.xhtml

[49] Minami Y, Yamamoto R, Nishikouri M, Fukao A, Hisamichi S. Mortality and cancer incidence in patients with Parkinson's disease. J Neurol 2000;247:429-434.

[50] Naghavi-Behzad M, Piri R. Risk of cancer among patients with Parkinson disease. Parkinsonism Relat Disord 2016;22:e34.

[51] Olsen JH, Friis S, Frederiksen K. Malignant melanoma and other types of cancer preceding Parkinson disease. Epidemiology 2006;17:582-587.

[52] Olsen JH, Friis S, Frederiksen K, McLaughlin JK, Mellemkjaer L, Moller H. Atypical cancer pattern in patients with Parkinson's disease. Br J Cancer 2005;92:201-205.

[53] Olsen JH, Tangerud K, Wermuth L, Frederiksen K, Friis S. Treatment with levodopa and risk for malignant melanoma. Mov Disord 2007;22:1252-1257.

[54] Ong EL, Goldacre R, Goldacre M. Differential risks of cancer types in people with Parkinson's disease: a national record-linkage study. Eur J Cancer 2014;50:2456-2462.

[55] Park JH, Kim DH, Park YG, et al. Cancer risk in patients with Parkinson's disease in South Korea: A nationwide, population-based cohort study. Eur J Cancer 2019;117:5-13.

[56] Peretz C, Gurel R, Rozani V, et al. Cancer incidence among Parkinson's disease patients in a 10-yrs time-window around disease onset: A large-scale cohort study. Parkinsonism Relat Disord 2016;28:68-72.

[57] Pinter B, Diem-Zangerl A, Wenning GK, et al. Mortality in Parkinson's disease: a 38-year follow-up study. Mov Disord 2015;30:266-269.

[58] Piri R, Naghavi-Behzad M. Association between cancers and risk of Parkinson disease: A cohort study. Parkinsonism Relat Disord 2016;22:e33.

[59] Powers KM, Smith-Weller T, Franklin GM, Longstreth WT, Jr., Swanson PD, Checkoway H. Diabetes, smoking, and other medical conditions in relation to Parkinson's disease risk. Parkinsonism Relat Disord 2006;12:185-189.

[60] Pressley JC, Louis ED, Tang MX, et al. The impact of comorbid disease and injuries on resource use and expenditures in parkinsonism. Neurology 2003;60:87-93.

[61] Rugbjerg K, Friis S, Lassen CF, Ritz B, Olsen JH. Malignant melanoma, breast cancer and other cancers in patients with Parkinson's disease. Int J Cancer 2012;131:1904-1911.

[62] Schwid SR, Bausch J, Oakes D, et al. Cancer incidence in a trial of an antiapoptotic agent for Parkinson's disease. Mov Disord 2010;25:1801-1808.

[63] Shalaby SY, Louis ED. Increased Odds of Melanoma: Parkinson's Disease, Essential Tremor, Dystonia versus Controls. Neuroepidemiology 2016;46:128-136.

[64] Sun LM, Liang JA, Chang SN, Sung FC, Muo CH, Kao CH. Analysis of Parkinson's disease and subsequent cancer risk in Taiwan: a nationwide population-based cohort study. Neuroepidemiology 2011;37:114-119.

[65] Tacik P, Curry S, Fujioka S, et al. Cancer in Parkinson's disease. Parkinsonism Relat Disord 2016;31:28-33.

For peer review only - http://bmjopen.bmj.com/site/about/guidelines.xhtml

[66] Tang CF, Lu MK, Muo CH, Tsai CH, Kao CH. Increased risk of brain tumor in patients with Parkinson's disease: a nationwide cohort study in Taiwan. Acta Neurol Scand 2016;134:148-153.

[67] Vanacore N, Spila-Alegiani S, Raschetti R, Meco G. Mortality cancer risk in parkinsonian patients: a population-based study. Neurology 1999;52:395-398.

[68] Wing K, Douglas I, Bhaskaran K, Smeeth L. A Cohort Analysis of the Association between Parkinson's Disease and Cancer Using the UK General Practice Research Database. Pharmacoepidemiol Drug Saf 2012;21:442-443.

[69] Winter AC, Rist PM, Buring JE, Kurth T. Prospective comorbidity-matched study of Parkinson's disease and risk of mortality among women. BMJ Open 2016;6:5.

[70] Wirdefeldt K, Weibull CE, Chen H, et al. Parkinson's disease and cancer: A register-based family study. Am J Epidemiol 2014;179:85-94.

[71] Agalliu I, Ortega RA, Luciano MS, et al. Cancer outcomes among Parkinson's disease patients with leucine rich repeat kinase 2 mutations, idiopathic Parkinson's disease patients, and nonaffected controls. Mov Disord 2019;34:1392-1398.

[72] Ording AG, Veres K, Horvath-Puho E, et al. Alzheimer's and Parkinson's Diseases and the Risk of Cancer: A Cohort Study. Journal of Alzheimer's disease : JAD 2019;72:1269-1277.

[73] Zhang P, Liu B. Association between Parkinson's Disease and Risk of Cancer: A PRISMA-compliant Meta-analysis. ACS Chem Neurosci 2019;10:4430-4439.

[74] Li X, Li W, Liu G, Shen X, Tang Y. Association between cigarette smoking and Parkinson's disease: A meta-analysis. Arch Gerontol Geriatr 2015;61:510-516.

[75] Evans AH, Lawrence AD, Potts J, et al. Relationship between impulsive sensation seeking traits, smoking, alcohol and caffeine intake, and Parkinson's disease. Journal of neurology, neurosurgery, and psychiatry 2006;77:317-321.

[76] Gong Y, Zack TI, Morris LG, et al. Pan-cancer genetic analysis identifies PARK2 as a master regulator of G1/S cyclins. Nature genetics 2014;46:588-594.

[77] Gupta A, Anjomani-Virmouni S, Koundouros N, et al. PARK2 Depletion Connects Energy and Oxidative Stress to PI3K/Akt Activation via PTEN S-Nitrosylation. Molecular cell 2017;65:999-1013.e1017.

[78] Agalliu I, San Luciano M, Mirelman A, et al. Higher frequency of certain cancers in LRRK2 G2019S mutation carriers with Parkinson disease: a pooled analysis. JAMA Neurol 2015;72:58-65.

[79] Feng DD, Cai W, Chen X. The associations between Parkinson's disease and cancer: the plot thickens. Transl Neurodegener 2015;4:20.

[80] Mass E, Jacome-Galarza CE, Blank T, et al. A somatic mutation in erythro-myeloid progenitors causes neurodegenerative disease. Nature 2017;549:389-393.

[81] Wahabi K, Perwez A, Rizvi MA. Parkin in Parkinson's Disease and Cancer: a Double-Edged Sword. Mol Neurobiol 2018;55:6788-6800.

[82] Driver JA. Inverse association between cancer and neurodegenerative disease: review of the epidemiologic and biological evidence. Biogerontology 2014;15:547-557.

For peer review only - http://bmjopen.bmj.com/site/about/guidelines.xhtml

[83] Ibanez K, Boullosa C, Tabares-Seisdedos R, Baudot A, Valencia A. Molecular evidence for the inverse comorbidity between central nervous system disorders and cancers detected by transcriptomic meta-analyses. PLoS Genet 2014;10:e1004173.

[84] Klus P, Cirillo D, Botta Orfila T, Gaetano Tartaglia G. Neurodegeneration and Cancer: Where the Disorder Prevails. Sci Rep 2015;5:15390.

[85] Gao X, Simon KC, Han J, Schwarzschild MA, Ascherio A. Family history of melanoma and Parkinson disease risk. Neurology 2009;73:1286-1291.

[86] Siple JF, Schneider DC, Wanlass WA, Rosenblatt BK. Levodopa therapy and the risk of malignant melanoma. The Annals of pharmacotherapy 2000;34:382-385.

[87] Bose A, Petsko GA, Eliezer D. Parkinson's Disease and Melanoma: Co-Occurrence and Mechanisms. J Parkinsons Dis 2018;8:385-398.

[88] Gao X, Simon KC, Han J, Schwarzschild MA, Ascherio A. Genetic determinants of hair color and Parkinson's disease risk. Ann Neurol 2009;65:76-82.

[89] Strange RC, Ellison T, Ichii-Jones F, et al. Cytochrome P450 CYP2D6 genotypes: association with hair colour, Breslow thickness and melanocyte stimulating hormone receptor alleles in patients with malignant melanoma. Pharmacogenetics 1999;9:269-276.

[90] Anwarullah, Aslam M, Badshah M, et al. Further evidence for the association of CYP2D6*4 gene polymorphism with Parkinson's disease: a case control study. Genes Environ 2017;39:18.

[91] Ascherio A, Schwarzschild MA. The epidemiology of Parkinson's disease: risk factors and prevention. The Lancet Neurology 2016;15:1257-1272.

[92] Gross A, Racette BA, Camacho-Soto A, Dube U, Searles Nielsen S. Use of medical care biases associations between Parkinson disease and other medical conditions. Neurology 2018;90:e2155-e2165.

For peer review only

For peer review only - http://bmjopen.bmj.com/site/about/guidelines.xhtml

Table 1. Association between Parkinson disease and cancer.

	No. of publications	Pooled RR (95% CI)	P for significance	P for heterogeneity
**Total cancer**				
All full-text publications	33	0.82 (0.76, 0.88)	<0.001	<0.001
Including abstracts	37	0.80 (0.74, 0.86)	<0.001	<0.001
Excluding mortality studies	25	0.85 (0.79, 0.92)	<0.001	<0.001
Excluding self-report diagnosis	31	0.81 (0.75, 0.87)	<0.001	<0.001
**Smoking-related cancer[1]**	21	0.76 (0.67, 0.85)	<0.001	<0.001
**Non-smoking-related cancer[2]**	19	0.92 (0.84, 0.99)	0.03	<0.001
**Site-specific cancer**				
Melanoma	27	1.75 (1.42, 2.15)	<0.001	<0.001
Non-melanoma skin cancer	15	0.85 (0.56, 1.30)	0.46	<0.001
Lung cancer	20	0.62 (0.51, 0.75)	<0.001	<0.001
Colorectal cancer	20	0.82 (0.75, 0.90)	<0.001	<0.001
Breast cancer	15	1.02 (0.93, 1.12)	0.66	0.001
Prostate cancer	17	0.93 (0.83, 1.03)	0.18	<0.001

[1]Smoking-related cancer includes cancer of the lung, larynx, mouth, esophagus, throat, bladder, kidney, liver, stomach, pancreas, colon and rectum, and cervix, as well as acute myeloid leukemia;

[2]Non-smoking-related cancer includes all other cancer except for those listed as smoking-related;

RR, relative risk; CI, confidence interval.

For peer review only - http://bmjopen.bmj.com/site/about/guidelines.xhtml

Figure 1. Flow chart.

Figure 2. Association between Parkinson's disease and total cancer in 33 publications. The figure shows the estimates (ESs) and 95% confidence intervals (CIs) for each study and the pooled result from random-effects model. Studies are stratified by temporal relationship of Parkinson's disease and cancer. M: men; W: women.

Figure 3. Association between Parkinson's disease and (A) smoking-related cancers in 21 publications, and (B) non-smoking-related cancers in 19 publications. Figure shows the estimates (ESs) and 95% confidence intervals (CIs) for each study and the pooled result from random effects model. Studies are stratified by temporal relationship of Parkinson's disease and cancer. M: men; W: women; *: pooled risk estimates calculated from individual ES in original publication.

Figure 4. Association between Parkinson's disease and (A) melanoma in 27 publications, and (B) non-melanoma skin cancers in 15 publications. Figure shows the estimates (ESs) and 95% confidence intervals (CIs) for each study and the pooled result from random effects model. Studies are stratified by temporal relationship of Parkinson's disease and cancer. M: men; W: women; BCC: basal cell carcinoma; SCC: squamous cell carcinoma; y: years of age.

For peer review only - http://bmjopen.bmj.com/site/about/guidelines.xhtml

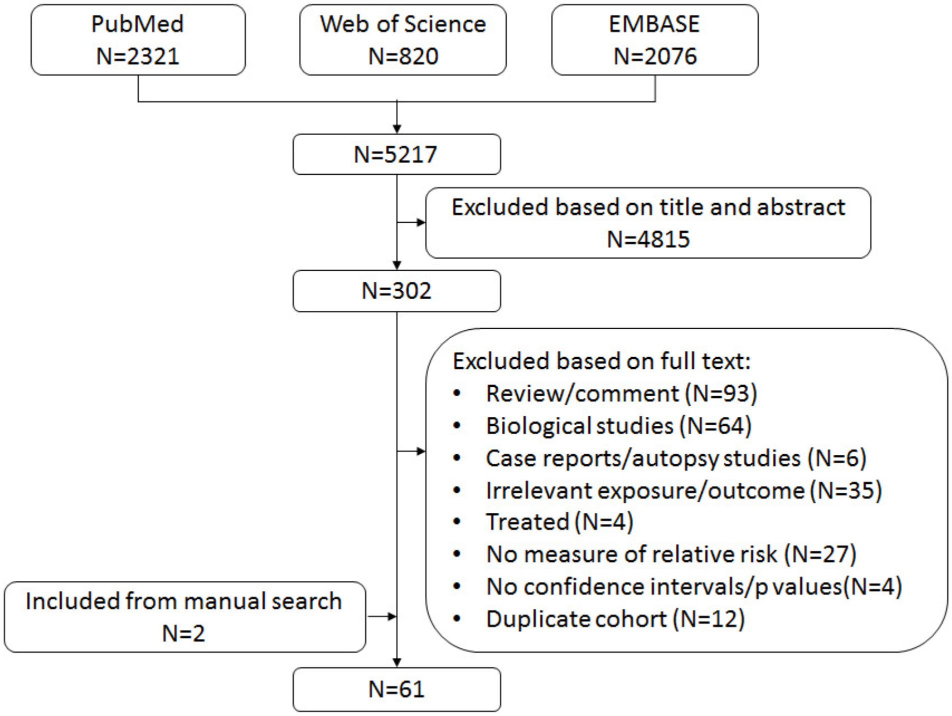

Figure 1. Flow chart.

315x233mm (72 x 72 DPI)

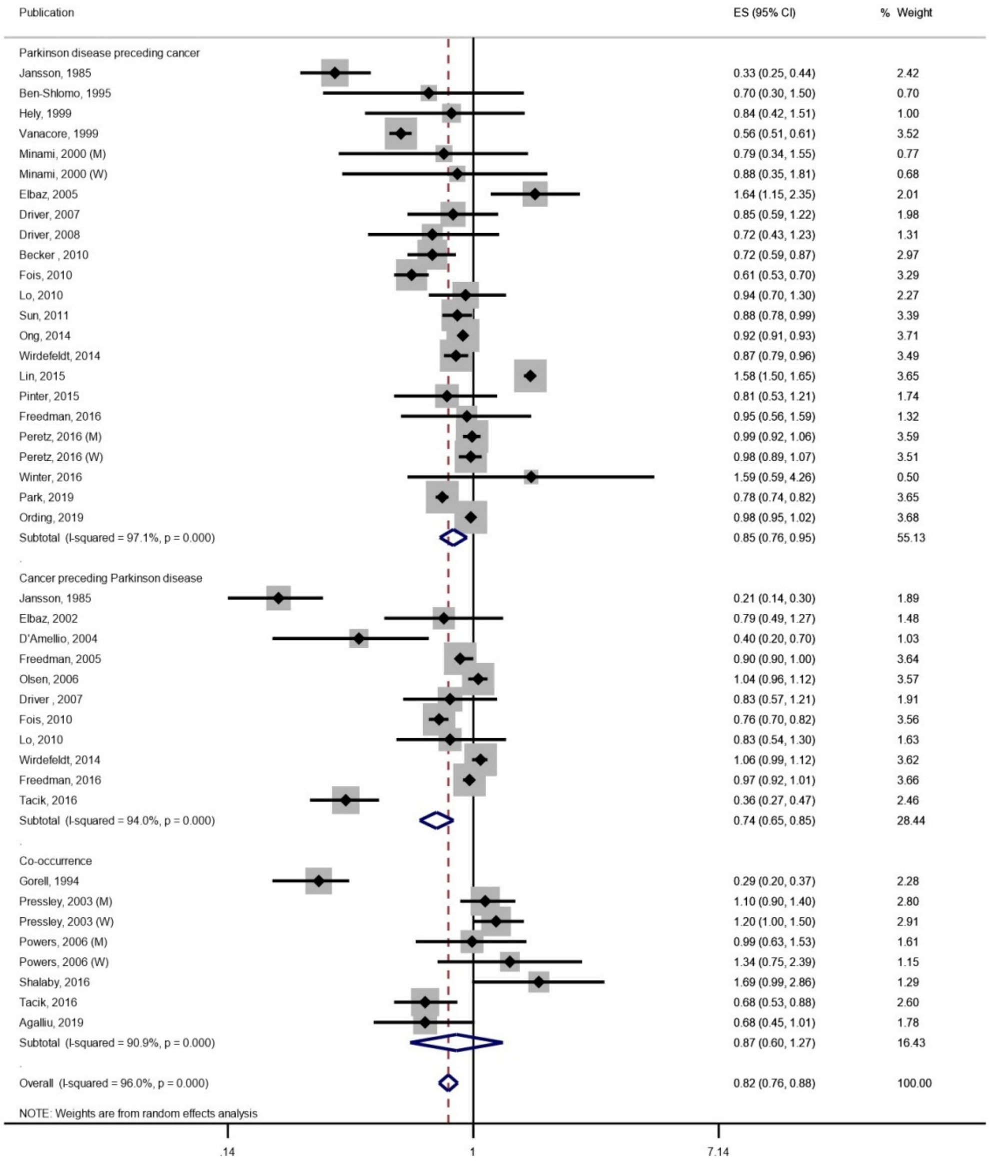

Figure 2. Association between Parkinson's disease and total cancer in 33 publications. The figure shows the estimates (ESs) and 95% confidence intervals (CIs) for each study and the pooled result from random-effects model. Studies are stratified by temporal relationship of Parkinson's disease and cancer. M: men; W: women.

239x283mm (150 x 150 DPI)

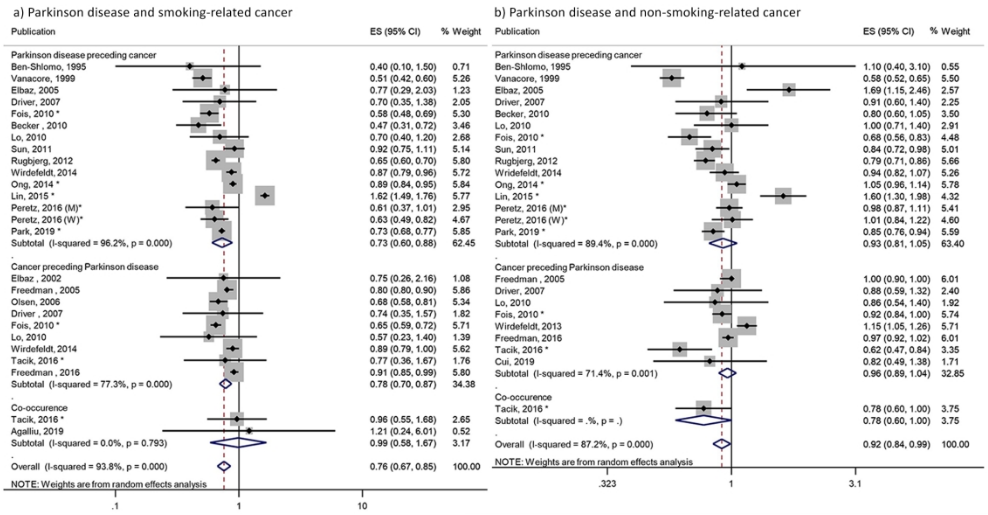

Figure 3. Association between Parkinson's disease and (A) smoking-related cancers in 21 publications, and (B) non-smoking-related cancers in 19 publications. Figure shows the estimates (ESs) and 95% confidence intervals (CIs) for each study and the pooled result from random effects model. Studies are stratified by temporal relationship of Parkinson's disease and cancer. M: men; W: women; *: pooled risk estimates calculated from individual ES in original publication.

763x406mm (72 x 72 DPI)

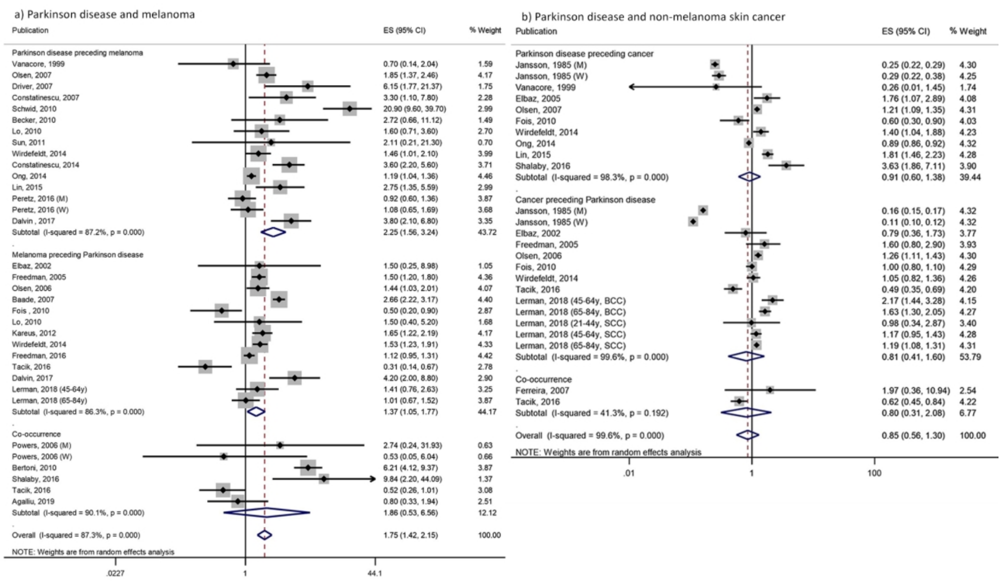

Figure 4. Association between Parkinson’s disease and (A) melanoma in 27 publications, and (B) non-melanoma skin cancers in 15 publications. Figure shows the estimates (ESs) and 95% confidence intervals (CIs) for each study and the pooled result from random effects model. Studies are stratified by temporal relationship of Parkinson’s disease and cancer. M: men; W: women; BCC: basal cell carcinoma; SCC: squamous cell carcinoma; y: years of age.

696x403mm (72 x 72 DPI)

Supplementary material for "Parkinson Disease and cancer: a systematic review and meta-analysis of 17,697,252 participants"

Content

Supplementary methods

Search strategy

Duplicate database inclusion/exclusion

Supplementary tables

Table 1. Characteristics of publications included in meta-analysis of Parkinson Disease and cancer.

Table 2. Meta regression and sub-group analysis on association between Parkinson disease and total cancer.

Table 3. Publications on risk of total cancer associated with levodopa treatment.

Supplementary figures

Figure 1. Funnel plot of studies of the association between Parkinson Disease and total cancer.

Figure 2. Association between use of levodopa and risk of total cancer in 4 publications.

Figure 3. Funnel plot of studies of the association between Parkinson Disease and a) smoking-related cancers, b) non-smoking-related cancers.

Figure 4. Funnel plot of studies of the association between Parkinson Disease and a) melanoma, b) non-melanoma skin cancers.

For peer review only

Supplementary methods

PubMed search strategy for Parkinson Disease and cancer

(("Parkinson Disease"[Mesh] OR "Parkinson Disease"[TW] OR "Parkinson's Disease"[TW] OR "Parkinsonism"[TW]) AND "cancer"[sb] AND ("Epidemiologic Studies"[Mesh] OR "Epidemiologic"[TW] OR "epidemiological"[TW] OR "Case-Control Studies"[Mesh] OR "case-control"[TW] OR "case control"[TW] OR "Case-Comparison"[TW] OR "Case Comparison"[TW] OR "Case-Compeer"[TW] OR "Case-Referent"[TW] OR "Case Referent"[TW] OR "Case-Base"[TW] OR "Case Base"[TW] OR "Cohort Studies"[Mesh] OR "cohort"[TW] OR "Concurrent"[TW] OR "Incidence"[TW] OR "Cross-Sectional Studies"[Mesh] OR "cross-sectional"[TW] OR "cross sectional"[TW] OR "Disease Frequency"[TW] OR "Prevalence"[TW] OR "Follow-Up Studies"[Mesh] OR "Follow-Up"[TW] OR "Follow Up"[TW] OR "Followup"[TW] OR "Longitudinal Studies"[Mesh] OR "longitudinal"[TW] OR "Retrospective Studies"[Mesh] OR "retrospective"[TW] OR "Prospective Studies"[Mesh] OR "prospective"[TW] OR "observational"[TW] OR "Observational Study" [Publication Type] OR "mortality studies"[TW] OR "ratio"[TW] OR "risk"[TW]) AND English[lang]) NOT ("animals"[MeSH Terms] NOT ("humans"[MeSH Terms] AND "animals"[MeSH Terms]))

Selection of publications that used same population

Leibson 2006, Elbaz 2002, Elbaz 2005, and Dalvin 2017 all used data from Mayo Clinic, Minnesota. Leibson 2006 was updated by Elbaz 2002 and 2005, therefore excluded from this meta-analysis. Elbaz 2002 studied PD risk after cancer, while Elbaz 2005 studied cancer risk after PD, therefore both publications were included. Dalvin 2017 was a cross-sectional extension of previous result, but it contained detailed analysis on melanoma, therefore it was not included in the analysis for total cancer, but was included for melanoma.

Olsen 2005, Olsen 2006, Olsen 2007, Rugbjerg 2012, Frandson 2014, Jespersen 2016, Cui 2019, and Ording 2019 all used National Hospital Register of Denmark. Frandson 2014 was a cross-sectional study that overlapped with Olsen 2006, Rugbjerg 2012, and Ording 2019, therefore was not included in this meta-analysis. Other publications varied in designs, time windows, temporal relationship, and cancer of interest.

For peer review only

There were also multiple publications from Physicians' Health Study, Women's Health Study, and Taiwan Health Registry. However, all of these groups of paper varied in designs, time windows, temporal relationship, and cancer of interest, therefore were not considered as duplicates. Other duplicates were meeting preceding/abstract of later-published articles, or duplicates that were not identified by matching in Endnote X9.

For peer review only

Supplementary tables

Table 1. Characteristics of publications included in meta-analysis of Parkinson Disease and cancer.

**Author**	**Year**	**Publication type**	**Direction**	**Study design**	**Location**	**Cohort**	**Case N**	**Control N**	**Follow time**	**Diagnosis**	**Smoking adjusted**	**Levodopa**
	2019		Co-occurrence	Cross-sectional	Europe, Israel, and the United States	Michael J. Fox Foundation	712	218	\	Diagnosed	No	No
Baade	2007	Article	Cancer preceding Parkinson Disease	Case-only cohort	Australia		127037		6.0	Coded	No	No
Becker	2010	Article	Parkinson Disease preceding cancer	1. Matched cohort 2. Case-control	UK	UK-based General Practice Research Database	466	1864	\	Validated	Yes	Yes
Ben-Shlomo	1995	Article	Parkinson Disease preceding cancer	Matched cohort	England and Wales	Second National Morbidity Study	220	421	\	Coded	No	No
Bermejo-Pareja	2012	Abstract	Parkinson Disease preceding cancer	Prospective cohort	Spain	Neurologic Disorders in Central Spain (NEDICES)	81	5197	\	\	No	No
Bertoni	2010	Article	Co-occurence	Case-only cohort	North America		2106		\	Diagnosed	No	No
Binagh	2016	Abstract	Co-occurence	Cross-sectional	Italy		529		\	Diagnosed	Yes	No
Boursi	2016	Article	Parkinson Disease preceding cancer	Case-control	UK	The Health Improvement Network	22093	85833	\	Diagnosed	Yes	Yes
Constatinescu	2007	Article	Parkinson Disease preceding cancer	Case-only cohort	North America	DATATOP	800		4.61	Diagnosed	No	No
Constatinescu	2014	Article	Parkinson Disease	Case-only cohort	US	NET-PD	1737		3.71	Diagnosed	No	No

For peer review only

For peer review only - http://bmjopen.bmj.com/site/about/guidelines.xhtml

			preceding cancer									
Cui	2019	Article	Cancer preceding Parkinson Disease	Case-control	Denmark	National Hospital Register	1813	1887	\	Diagnosed	Yes	No
Dalvin	2017	Article	Both	1. Case-control 2. Matched cohort	Minnesota	Mayo clinic	974	2922	5	Coded	No	No
D'Amellio	2004	Article	Cancer preceding Parkinson Disease	Case-control	Italy		222	222	\	Diagnosed	Yes	No
Driver	2007	Article	Parkinson Disease preceding cancer	Matched cohort	US	Physicians' Health Study	487	487	5.2	Validated	Yes	No
Driver	2007	Article	Cancer preceding Parkinson Disease	Case-control	US	Physicians' Health Study	487	487	\	Validated	Yes	No
Driver	2008	Article	Parkinson Disease preceding cancer	Matched cohort	US	Physicians' Health Study	560	560	5.8	Validated	Yes	No
Elbaz	2002	Article	Cancer preceding Parkinson Disease	Case-control	Minnesota	Mayo clinic	196	196	5.5	Diagnosed	No	No
Elbaz	2005	Article	Parkinson Disease preceding cancer	Matched cohort	Minnesota	Mayo clinic	196	185	8	Diagnosed	Yes	Yes
Fall	2003	Article	Parkinson Disease preceding cancer	Matched cohort	Sweden		170	510	4.8	Diagnosed	No	No
Ferreira	2007	Article	Co-occurence	Cross-sectional	Portugal	The Lisbon University Hospital	150	146	\	Diagnosed	No	No

For peer review only

Fois	2010	Article	Both	Case-only cohort	UK	Oxford Record Linkage Study	4355		3.2	Coded	No	No
Freedman	2005	Article	Cancer preceding Parkinson Disease	Case-only cohort	US	SEER-Medicare	1900000		8.5	Coded	No	No
Freedman	2016	Letter	Parkinson Disease preceding cancer	Case-control	US (Asian Americans)	SEER-Medicare	20627	5558	\	Coded	No	No
Freedman	2016	Article	Cancer preceding Parkinson Disease	1. Case-control 2. cohort	US	SEER-Medicare	743779	419432	2.8	Coded	No	No
Gorell	1994	Article	Co-occurence	Cross-sectional	Michigan		8629	208933	\	Coded	No	No
Hely	1999	Article	Parkinson Disease preceding cancer	Case-only cohort	Australia	Sydney Multicenter Study of PD	130		9.1	Diagnosed	No	No
Jansson	1985	Article	Both	Prospective cohort	US		406		8.6	Diagnosed	Yes	No
Jamrozik	2005	Abstract	Cancer preceding Parkinson Disease	Case-control	Poland		100	100	\	\	No	No
Jespersen	2016	Article	Co-occurence	Case-control	Denmark	National Registry	45429	227145	\	Coded	No	No
Kareus	2012	Article	Cancer preceding Parkinson Disease	Case-control	US	Utah Cancer Registry	2300000			Coded	No	No
Kelm	2018	Abstract	Co-occurence	Case-control	US	Northwestern Medicine Enterprise Data Warehouse medical record	4751	9494	5.75	Coded	No	Yes

For peer review only

Lai	2013	Letter	Co-occurence	Case-control	Taiwan	National Health	2822	11288	\	Coded	Yes	No
Lai	2015	Article	Co-occurence	Case-control	Taiwan	National Health	1815	7260	\	Coded	No	No
Lerman	2018	Article	Parkinson Disease preceding cancer	Prospective cohort	Isreal	Maccabi Health Services	7727	1243968	\	Coded	Yes	No
Liao	2015	Article	Parkinson Disease preceding cancer	Case-control	Taiwan	National Health	13861	55444	\	Coded	No	No
Liao	2017	Article	Parkinson Disease preceding cancer	Case-control	Taiwan	National Health	64619	64619	\	Coded	No	No
Lin	2015	Article	Parkinson Disease preceding cancer	Matched cohort	Taiwan	National Health	62023	124046	\	Coded	No	No
Lo	2010	Article	Both	Matched cohort	US	PEAK	692	761	5.0; 4.3	Diagnosed	Yes	No
Minami	2000	Article	Parkinson Disease preceding cancer	Case-only cohort	Japan		228		6.97	Validated	No	No
Naghavi-Behzad	2016	Abstract	Parkinson Disease preceding cancer	Case-control	Iran		\		\	\	No	No
Olsen	2005	Denmark	Parkinson Disease preceding cancer	National Hospital Register			14088		5.0	Coded	No	No
Olsen	2006	Article	Cancer preceding Parkinson Disease	Case-control	Denmark	National Hospital Register	8090	32320	\	Coded	No	No
Olsen	2007	Article	Parkinson Disease preceding cancer	Case-only cohort	Denmark	National Hospital Register	14088		\	Coded	No	Yes

For peer review only

Ong	2014	Article	Parkinson Disease preceding cancer	Prospective cohort	UK	NHS hospital	219194	9015614	\	Coded	No	No
Ording	2019	Article	Parkinson Disease preceding cancer	Case-only cohort	Denmark	National Hospital Register	28835		4.0	Coded	No	No
Park	2019	Article	Parkinson Disease preceding cancer	Matched cohort	South Korea	NHI	52009	260045	\	Coded	No	No
Peretz	2016	Article	Parkinson Disease preceding cancer	Case-only cohort	Israel	Maccabi Health Services	7125		10.5	Validated	No	No
Pinter	2015	Article	Parkinson Disease preceding cancer	Case-only cohort	Austria		237		14.8	Coded	No	No
Piri	2016	Abstract	Parkinson Disease preceding cancer	Prospective cohort		Cancer Registry Database	2584		\	Diagnosed	No	No
Powers	2006	Article	Co-occurence	Case-control	Seattle		352	484	\	Diagnosed	Yes	No
Pressley	2003	Article	Co-occurence	Cross-sectional	US	National Long-Term Care Survey	791	24040	\	Coded	No	No
Rugbjerg	2012	Article	Parkinson Disease preceding cancer	Case-only cohort	Denmark	National Hospital Register	20343		5.7	Coded	No	No
Schwid	2010	Article	Parkinson Disease preceding cancer	Case-only cohort	US	PRECEPT	806		1.8	Diagnosed /verified	No	No
Shalaby	2016	Article	Co-occurence	Case-control	US	Columbia University Medical Cente	108	124	\	Self-report	No	No

For peer review only

Sun	2011	Article	Parkinson Disease preceding cancer	Matched cohort	Taiwan	NHI	4957	19828	\	Coded	No	No
Tacik	2016	Article	1. Co-occurrence 2. cancer preceding PD	Prospective cohort	Florida	Mayo clinic	971	478	4.6	Diagnosed	No	No
Tang	2016	Article	Parkinson Disease preceding cancer	Matched cohort	Taiwan	NHI	2998	11992	\	Coded	No	No
Vanacore	1999	Communication	Parkinson Disease preceding cancer	Case-only cohort	Italy		10322		5.7	Drug	No	No
Wing	2012	Abstract	Both	Prospective cohort	UK		8549	42160	\	\	Yes	No
Winter	2016	Article	Parkinson Disease preceding cancer	Matched cohort	US	Women's Health Study	396	396	6.2	Self-report	Yes	No
Wirdefeldt	2014	Article	Both	Matched cohort	Sweden		11786	58930	\	Coded	No	No

For peer review only

Table 2. Subgroup-analysis of the association between Parkinson disease and cancer.

	No. of publications	Pooled RR (95% CI)	P for significance	P for heterogeneity	P difference
**Age**					**0.10**
< 69.3 years	13	0.70 (0.42, 1.19)	0.21	<0.001	
≥ 69.3 years	14	0.90 (0.81, 1.00)	0.05	<0.001	
**Sex**					**0.31**
Men-dominant	23	0.76 (0.57, 1.02)	0.07	<0.001	
Women-dominant	12	0.91 (0.70, 1.17)	0.45	<0.001	
**Ethnicity**					**0.19**
Caucasian-dominant	27	0.75 (0.59, 0.96)	0.02	<0.001	
Asian-dominant	6	0.98 (0.75, 1.28)	0.88	<0.001	
**Study design**					**0.92**
Prospective cohort	24	0.79 (0.65, 0.96)	0.05	<0.001	
Other	9	0.79 (0.65, 0.96)	0.02	<0.001	
**Newcastle-Ottawa quality score**					**0.31**
≤ 6	12	0.87 (0.71, 1.08)	0.21	<0.001	
≥ 7	21	0.75 (0.57, 0.98)	0.04	<0.001	
**Period of study**					**0.19**
< 2010	16	0.73 (0.61, 0.88)	0.001	<0.001	
≥ 2010	17	0.88 (0.80, 0.96)	0.003	<0.001	

6 publications did not report mean/median age or age range.

2 publications did not report sex ratio. 4 publications separately report risk estimates for men and women, therefore counted in both sex groups.

For peer review only

Table 3. Publications on risk of total cancer associated with levodopa treatment.

Publication	Estimation (95% confidence interval)	Note
Elbaz, 2005	1.26 (0.39, 4.12)	4th (>1,313 g) compared to 1st quartile of cumulative levodopa
Constanescu, 2007	1.4 (0.3, 4.3)	After levodopa use
Olsen, 2007	1.0 (0.5, 2.0)	≥1370 g compared to 600-1369 g of cumulative levodopa
Becker, 2010	0.7 (0.56, 0.88)	≥5 prescription of levodopa

For peer review only

Supplementary figures

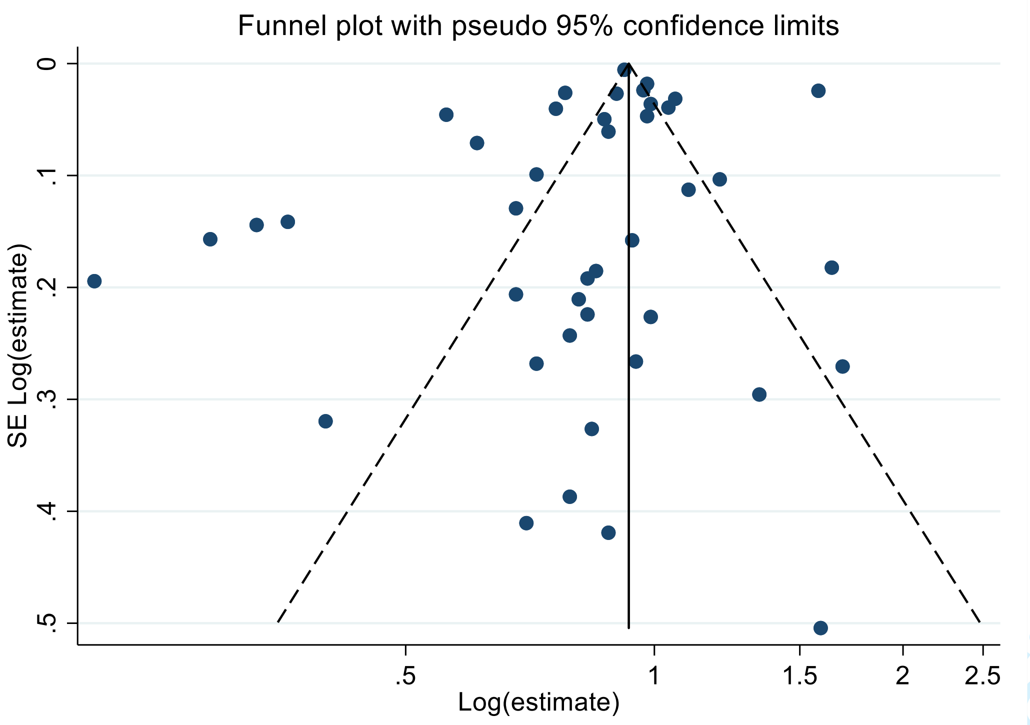

Figure 1. Funnel plot of studies of the association between Parkinson Disease and total cancer. The log-transformed risk estimates from each study is plotted on the horizontal axis, and its standard error is plotted on the vertical axis.

For peer review only - http://bmjopen.bmj.com/site/about/guidelines.xhtml

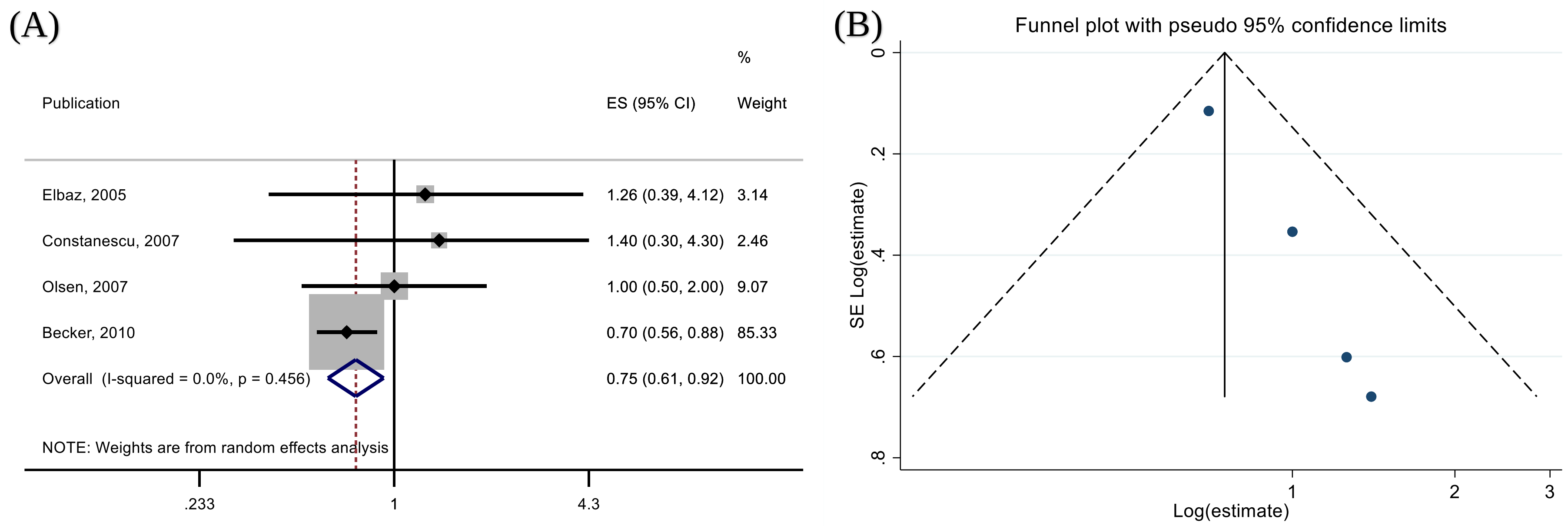

Figure 2. a) Individual and pooled estimates of the association between use of levodopa and risk of total cancer in 4 publications. Figure shows the estimates (ESs) and 95% confidence intervals (CIs) for each individual studies and the pooled result from random effects model. b) Funnel plots of these 4 publications.

For peer review only

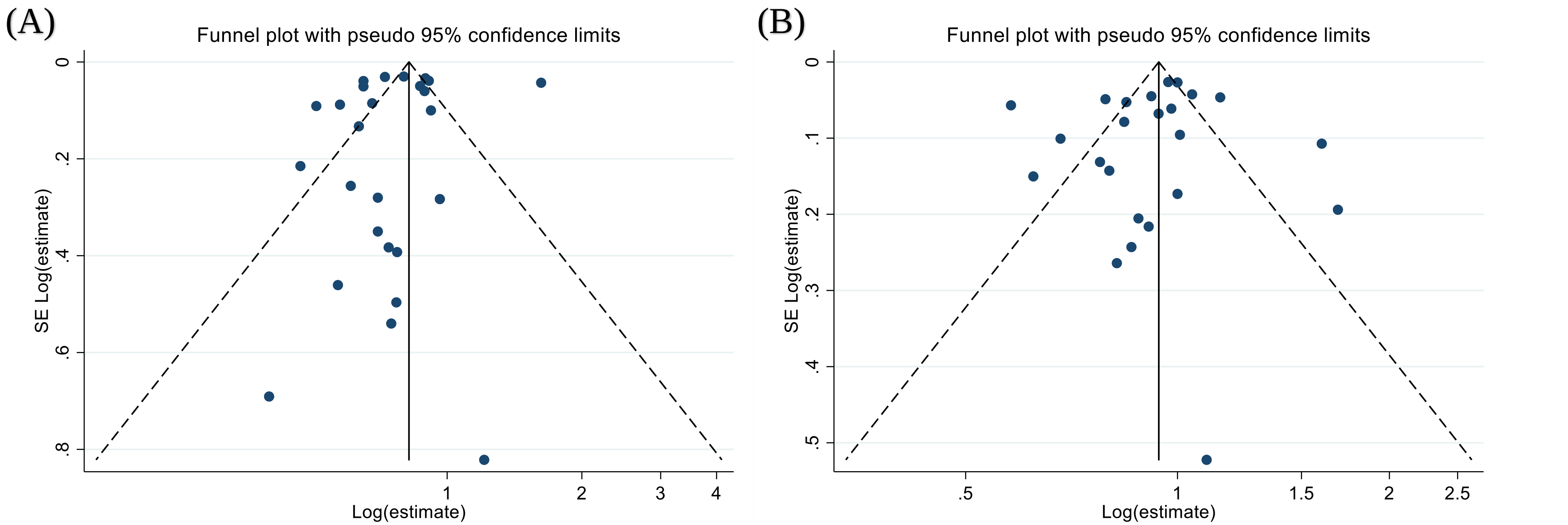

Figure 3. Funnel plot of studies of the association between Parkinson Disease and a) smoking-related cancers, b) non-smoking-related cancers. The log-transformed risk estimates from each study is plotted on the horizontal axis, and its standard error is plotted on the vertical axis.

For peer review only

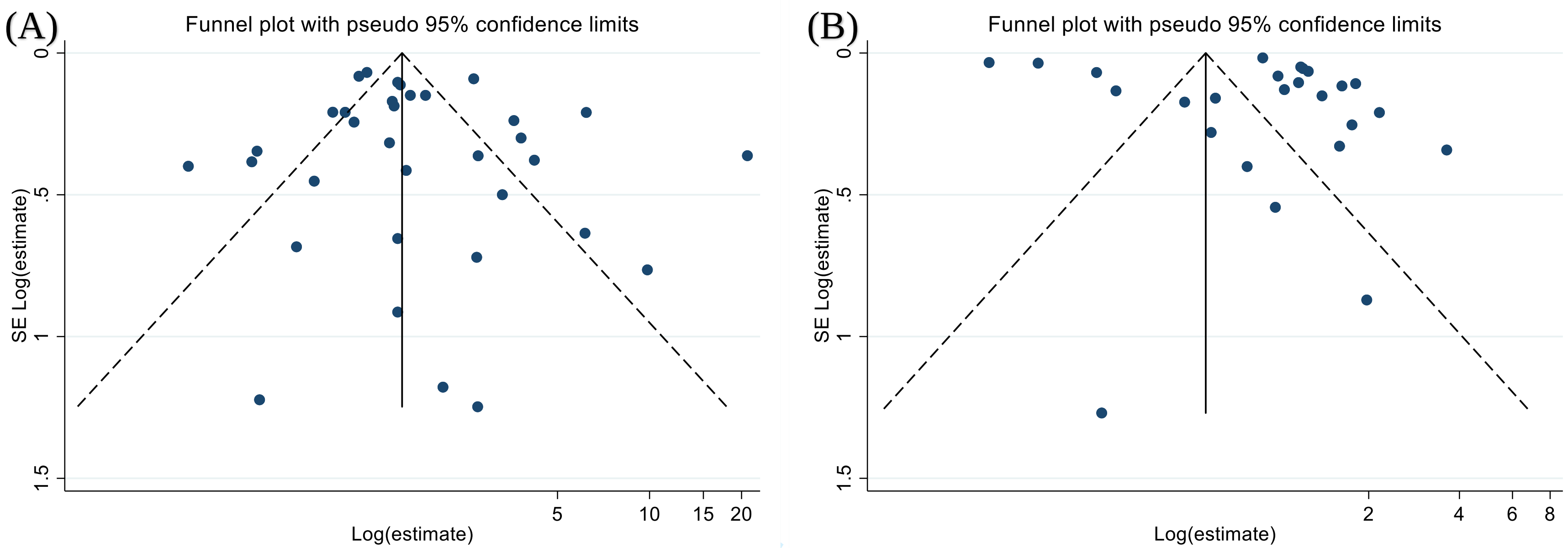

Figure 4. Funnel plot of studies of the association between Parkinson Disease and a) melanoma, b) non-melanoma skin cancers. The log-transformed risk estimates from each study is plotted on the horizontal axis, and its standard error is plotted on the vertical axis.

PRISMA 2009 Checklist

Section/topic	#	Checklist item	Reported on page #
**TITLE**			
Title	1	Identify the report as a systematic review, meta-analysis, or both.	1
**ABSTRACT**			
Structured summary	2	Provide a structured summary including, as applicable: background; objectives; data sources; study eligibility criteria, participants, and interventions; study appraisal and synthesis methods; results; limitations; conclusions and implications of key findings; systematic review registration number.	2 & 3
**INTRODUCTION**			
Rationale	3	Describe the rationale for the review in the context of what is already known.	5
Objectives	4	Provide an explicit statement of questions being addressed with reference to participants, interventions, comparisons, outcomes, and study design (PICOS).	5
**METHODS**			
Protocol and registration	5	Indicate if a review protocol exists, if and where it can be accessed (e.g., Web address), and, if available, provide registration information including registration number.	7 and supplementary material
Eligibility criteria	6	Specify study characteristics (e.g., PICOS, length of follow-up) and report characteristics (e.g., years considered, language, publication status) used as criteria for eligibility, giving rationale.	7
Information sources	7	Describe all information sources (e.g., databases with dates of coverage, contact with study authors to identify additional studies) in the search and date last searched.	7
Search	8	Present full electronic search strategy for at least one database, including any limits used, such that it could be repeated.	7
Study selection	9	State the process for selecting studies (i.e., screening, eligibility, included in systematic review, and, if applicable, included in the meta-analysis).	7
Data collection process	10	Describe method of data extraction from reports (e.g., piloted forms, independently, in duplicate) and any processes for obtaining and confirming data from investigators.	8
Data items	11	List and define all variables for which data were sought (e.g., PICOS, funding sources) and any assumptions and simplifications made.	8
Risk of bias in individual studies	12	Describe methods used for assessing risk of bias of individual studies (including specification of whether this was done at the study or outcome level), and how this information is to be used in any data synthesis.	8
Summary measures	13	State the principal summary measures (e.g., risk ratio, difference in means).	8

For peer review only

PRISMA 2009 Checklist

Synthesis of results	14	Describe the methods of handling data and combining results of studies, if done, including measures of consistency (e.g., I^2) for each meta-analysis.	9

Section/topic	#	Checklist item	Reported on page #
Risk of bias across studies	15	Specify any assessment of risk of bias that may affect the cumulative evidence (e.g., publication bias, selective reporting within studies).	8
Additional analyses	16	Describe methods of additional analyses (e.g., sensitivity or subgroup analyses, meta-regression), if done, indicating which were pre-specified.	9
**RESULTS**			
Study selection	17	Give numbers of studies screened, assessed for eligibility, and included in the review, with reasons for exclusions at each stage, ideally with a flow diagram.	10 and supplementary material
Study characteristics	18	For each study, present characteristics for which data were extracted (e.g., study size, PICOS, follow-up period) and provide the citations.	10, Table 1, and Supplementary material
Risk of bias within studies	19	Present data on risk of bias of each study and, if available, any outcome level assessment (see item 12).	10, 11, and supplementary material
Results of individual studies	20	For all outcomes considered (benefits or harms), present, for each study: (a) simple summary data for each intervention group (b) effect estimates and confidence intervals, ideally with a forest plot.	10, 11, Figure 1-4
Synthesis of results	21	Present results of each meta-analysis done, including confidence intervals and measures of consistency.	10, 11
Risk of bias across studies	22	Present results of any assessment of risk of bias across studies (see Item 15).	10, 11, and supplementary material
Additional analysis	23	Give results of additional analyses, if done (e.g., sensitivity or subgroup analyses, meta-regression [see Item 16]).	10
**DISCUSSION**			
Summary of evidence	24	Summarize the main findings including the strength of evidence for each main outcome; consider their relevance to key groups (e.g., healthcare providers, users, and policy makers).	12-15

For peer review only

PRISMA 2009 Checklist

Limitations	25	Discuss limitations at study and outcome level (e.g., risk of bias), and at review-level (e.g., incomplete retrieval of identified research, reporting bias).	12,14,15
Conclusions	26	Provide a general interpretation of the results in the context of other evidence, and implications for future research.	15
**FUNDING**			
Funding	27	Describe sources of funding for the systematic review and other support (e.g., supply of data); role of funders for the systematic review.	16

From: Moher D, Liberati A, Tetzlaff J, Altman DG, The PRISMA Group (2009). Preferred Reporting Items for Systematic Reviews and Meta-Analyses: The PRISMA Statement. PLoS Med 6(7): e1000097. doi:10.1371/journal.pmed1000097

For more information, visit: **www.prisma-statement.org**.

For peer review only

For peer review only - http://bmjopen.bmj.com/site/about/guidelines.xhtml

BMJ Open

Parkinson's disease and cancer: a systematic review and meta-analysis of over 17 million participants

Journal:	*BMJ Open*
Manuscript ID	bmjopen-2020-046329.R1
Article Type:	Original research
Date Submitted by the Author:	03-May-2021
Complete List of Authors:	Zhang, Xinyaun; The Pennsylvania State University, Nutritional Science<br>Guarin, David; Massachusetts General Hospital, Neurology<br>Mohammadzadehhonarvar, Niyaz; Massachusetts General Hospital, Neurology<br>Chen, Xiqun; Massachusetts General Hospital, Neurology<br>Gao, Xiang; The Pennsylvania State University, Nutritional Science
**Primary Subject Heading**:	Epidemiology
Secondary Subject Heading:	Neurology
Keywords:	ONCOLOGY, EPIDEMIOLOGY, Parkinson-s disease < NEUROLOGY, Epidemiology < ONCOLOGY

SCHOLARONE™ Manuscripts

I, the Submitting Author has the right to grant and does grant on behalf of all authors of the Work (as defined in the below author licence), an exclusive licence and/or a non-exclusive licence for contributions from authors who are: i) UK Crown employees; ii) where BMJ has agreed a CC-BY licence shall apply, and/or iii) in accordance with the terms applicable for US Federal Government officers or employees acting as part of their official duties; on a worldwide, perpetual, irrevocable, royalty-free basis to BMJ Publishing Group Ltd ("BMJ") its licensees and where the relevant Journal is co-owned by BMJ to the co-owners of the Journal, to publish the Work in this journal and any other BMJ products and to exploit all rights, as set out in our licence.

The Submitting Author accepts and understands that any supply made under these terms is made by BMJ to the Submitting Author unless you are acting as an employee on behalf of your employer or a postgraduate student of an affiliated institution which is paying any applicable article publishing charge ("APC") for Open Access articles. Where the Submitting Author wishes to make the Work available on an Open Access basis (and intends to pay the relevant APC), the terms of reuse of such Open Access shall be governed by a Creative Commons licence – details of these licences and which Creative Commons licence will apply to this Work are set out in our licence referred to above.

Other than as permitted in any relevant BMJ Author's Self Archiving Policies, I confirm this Work has not been accepted for publication elsewhere, is not being considered for publication elsewhere and does not duplicate material already published. I confirm all authors consent to publication of this Work and authorise the granting of this licence.

For peer review only

Parkinson's disease and cancer: a systematic review and meta-analysis of over 17 million participants

Xinyuan Zhang, BS[1#], David Guarin, BA[2#], Niyaz Mohammadzadeh honarvar,PhD[2], Xiqun Chen, MD, PhD[1*], Xiang Gao, MD, PhD[2*]

[1]Department of Nutritional Sciences, The Pennsylvania State University, University Park, PA 16802, USA

[2]Department of Neurology, Massachusetts General Hospital, Harvard Medical School, Boston, MA 02114, USA

#Co-first author; *Co-senior author

Correspondence should be addressed to Xiqun Chen**;** xchen17@mgh.harvard.edu

Word Count: 2690

Keywords: Neoplasms; Epidemiology; Meta-analysis; Parkinson's disease; Odds ratio.

For peer review only - http://bmjopen.bmj.com/site/about/guidelines.xhtml

Abstract

Objective

To systematically review and qualitatively evaluate epidemiological evidence on associations between PD and cancer via meta-analysis.

Data Sources

MEDLINE via PubMed, Web of Science, and EMBASE, until March 2021.

Study Selection

Included were publications that 1) were original epidemiological studies on PD and cancer; 2) reported risk estimates; 3) were in English. Exclusion criteria included: 1) review/comments; 2) biological studies; 3) case report/autopsy studies; 4) irrelevant exposure/outcome; 5) treated cases; 6) no measure of risk estimates; 7) no confidence intervals/exact p values; and 8) duplicates.

Data extraction and Synthesis

PRISMA and MOOSE guidelines were followed in data extraction. Two-step screening was performed by two authors blinded to each other. A random-effects model was used to calculate pooled relative risk (RR).

Main Outcomes and Measures

We included publications that assessed the risk of PD in individuals with vs without cancer and the risk of cancer in individuals with vs without PD.

Results

For peer review only

For peer review only - http://bmjopen.bmj.com/site/about/guidelines.xhtml

A total of 63 studies and 17,994,584 participants were included. Meta-analysis generated a pooled relative risk of 0.82 (n = 33; 95% CI: 0.76, 0.88; p <0.001) for association between PD and total cancer, 0.76 (n = 21; 95% CI: 0.67, 0.85; p <0.001) for PD and smoking-related cancer, and 0.92 (n = 19; 95% CI: 0.84, 0.99; p = 0.03) for non-smoking-related cancer. PD was associated with an increased risk of melanoma (n = 29; pooled relative risk = 1.75; 95% CI: 1.43, 2.14; p <0.001) but not for other skin cancers (n=17; pooled relative risk = 0.90; 95%CI: 0.60, 1.34; p = 0.60).

Conclusions

PD and total cancer were inversely associated. This inverse association persisted for both smoking-related and non-smoking-related cancers. PD was positively associated with melanoma. These results provide evidence for further investigations for possible mechanistic associations between PD and cancer.

For peer review only

Article Summary

Strengths and limitations of this study

- Unlike recent meta-analyses, this study stratifies analysis for smoking vs non-smoking cancers.
- Heterogeneity between included studies was analyzed via meta-regression.
- Despite best efforts, high heterogeneity in methodology and cohorts of included studies cannot be fully dealt with by statistical methods.

For peer review only

For peer review only - http://bmjopen.bmj.com/site/about/guidelines.xhtml

Introduction

Parkinson's disease (PD) is the second most common neurodegenerative disease affecting more than 10 milion people worldwide. It is characterized by premature cell death of dopaminergic neurons in the substantia nigra pars compacta (SNpc) . Clinically, PD is manifested by tremor, rigidity, bradykinesia and postural instability. Non-motor symtoms are also common. Symptomatic treatments for PD are available and effective, however there is currently no therapy known to modify disease progression. Among enviromental factors that have been associated with the risk of developing PD, age is the main risk factor, whereas smoking has been inversely associated with PD[1 2]. Familial PD accounts for 5%–15% of total PD. The most common genetic cause of PD is mutations in *LRRK2*. Other PD-related genes include *PARK2*, *PARK7*, *PINK1*, and *SNCA*. PD is increasingly recognized as a systemic disorder. Oxidative stress, mitochodia dysfuntion, energy failure, immune dysregulation and chornic inflammation have been proposed to contribute to neurodegeneration in PD [3].

Cancer is characterized by uncontrolled cell proliferation and growth. It is among the leading causes of death worldwide [4]. Growing evidence suggests that PD and cancer may be associated [5]. Similar to PD, cancer incidence increases with age [6]. Smoking also modifies the risk of certain cancer, especially lung cancer, though in the opposite direction to the risk of PD [7]. In addition, PD related genes have been implicated in cancer. *PARK2* has been identified as a potent tumor suppressor gene, whereas mutations in *LRRK2* have been associated with an increased risk of cancer [8]. While a positive, bidirectional link between PD and melanoma, a malignant tumor that develops from melanocytes is well-documented [9], there appears to be an inverse association between PD and total cancer [10]. However, it remains unclear whether PD and cancer are associated mechanistically, or the findings were confounded by other factors, such as study

For peer review only - http://bmjopen.bmj.com/site/about/guidelines.xhtml

designs and smoking. Clearly documenting these associations is important for bridging the interdisciplinary knowledge gap and developing novel preventive and treatment strategies for both PD and cancer. An individual study may lack the power to detect an association. A meta-analysis can increase precision in estimating risk [11], especially in subsets of cancers with even fewer cases. We thus conducted a meta-analysis to systematically review the population-based evidence for the potential association between PD and cancer. To better elucidate PD-cancer relation, we first stratified studies according to the temporal association between the two diseases into three categories: PD preceding cancer, cancer preceding PD, and co-occurrence. Secondly, we performed sensitivity analyses in which variations in study design and qualities, and levodopa treatment, were evaluated. Thirdly, we separately analyzed smoking-related cancers and non-smoking-related cancers to address smoking as a potential confounding factor. Finally, we specifically analyzed the associations between PD and melanoma, non-melanoma skin cancers, and other major cancers (eg, prostate cancer, colon cancer, and breast cancer).

Methods

Literature search and data extraction

This meta-analysis followed the MOOSE guidelines for reporting meta-analysis on observational studies and was registered on PROSPERO (CRD42020162103). We searched all published literature that reported PD association with cancer in MEDLINE via PubMed, Web of Science, and EMBASE up to March 1, 2021. Search items related to "Parkinson's disease", "cancer", and "epidemiologic studies" were identified and modified for each database. We constrained our search in human studies and in the English language. Detailed search terms can be found in

For peer review only - http://bmjopen.bmj.com/site/about/guidelines.xhtml

Supplementary materials. Duplicates were matched based on author, year, and title in Endnote X9 and manually compared before removing.

The inclusion criteria were: 1) original studies that were conducted in an epidemiological setting; 2) studies reported either an odds ratio (OR), risk ratio (RR), hazard ratio (HR), standardized incidence/mortality ratio (SIR/SMR), or other reliable measures of estimated risk; 3) studies in which PD and cancer cases were ascertained by doctor's diagnosis, hospitalization record, disease identification codes, or self-report on the diagnosis. Exclusion criteria included: 1) reviews or comments; 2) non-epidemiological studies; 3) case reports/autopsy studies; 4) irrelevant exposure/outcome; 5) treated cases; 6) no measure of risk estimates; 7) no confidence intervals/exact p values; and 8) duplicates. Parkinsonism that does not meet the criteria for PD and benign neoplasm were not included. Previous meta-analyses were used as references for manual searching of related publications. Two first authors (X. Z., BS and D. G., BA) independently screened the publications in two steps: title/abstract screening and full-text screening. Any discrepancy was reviewed and reconciled by two senior authors (X. C. and X. G.). During full-text screening, we found 5 groups of publications using the same population or dataset. Details of inclusion and exclusion step are reported in Supplementary methods. After screening references of included publications, we found two other eligible publications that were not captured by search items [12 13].

Data extraction

From each of the included publications, we extracted information on the first author, year of the study, study type, country origin, population, mean age, dominant sex, dominant ethnicity, cases and controls population size, measure of risk, PD and cancer ascertainment methods, adjusted

For peer review only - http://bmjopen.bmj.com/site/about/guidelines.xhtml

covariates, levodopa use, and estimated risk with lower and upper confidence intervals (CIs) for each type of cancer. The temporal association was defined per each individual study definition, most of which was based on the diagnosis date of the two diseases. Dominant sex and ethnicity were defined as the major sex and race/ethnicity (>50%) of the studied population, respectively.

The type of study was categorized into prospective study, case-control study, case-only cohort study, and cross-sectional study.

Statistical analysis

All analyses were performed in STATA SE 15. Cochran's Q statistic and I-squared were calculated to examine heterogeneity among studies. Cochran's Q was computed as the sum of variance from the pooled estimates and compared to chi-squared distribution with k-1 (k = number of publications) degree of freedom. I-squared was calculated as the percentage of variation across studies due to heterogeneity rather than chance [14]. Due to the high heterogeneity of included publications (p-value for Q statistics <0.05, I-squared >50% for all), pooled effect sizes (including RR, OR, HR, SIR, and SMR) were calculated using random-effects models to account for unobserved heterogeneity. Egger test and funnel plots were performed to assess publication bias.

For total cancer, we performed three sensitivity analyses. First, 4 publications from meeting proceedings/abstracts were further included; second, 8 mortality publications were excluded; third, 2 publications using invalidated, self-report diagnosis of either cancer or PD were excluded. Further, we performed six subgroup analyses, looking at the variance of the included publications in population age, dominant sex, dominant race/ethnicity, study design, study quality, and year of study. Age was separated into two groups by the mean age of the included

For peer review only - http://bmjopen.bmj.com/site/about/guidelines.xhtml

studies (69.3 years). Dominant ethnicity was categorized into Caucasian-dominant and Asian-dominant. The study design was categorized into cohort studies and other types of studies. Study quality was assessed by the Newcastle-Ottawa Scale for cohort studies and for case-control studies [15]], which is based on the definition of case/control, the definition of exposure/outcome, covariates, and other relevant factors. The score ranged from 0–9, and we separated the included studies into low quality group (< 7) and high quality group (≥7), based on the mean quality score of the included studies. Proceedings/abstracts were not included in the quality check. The difference between groups was tested by the meta-regression method.

We categorized cancers into smoking-related and non-smoking-related cancers according to National Cancer Institute and Centers for Disease Control and Prevention's definition [16]. Smoking-related cancers include cancer of the lung, larynx, mouth, esophagus, throat, bladder, kidney, liver, stomach, pancreas, colon and rectum, and cervix, as well as acute myeloid leukemia. Cancers of other sites, including melanoma, were regarded as not associated with smoking. If a publication reported grouped smoking- and non-smoking-related cancers, the risk estimates were extracted directly. If a publication reported individual cancers only, and the number of sites is more than 10, we first categorized individual cancers into smoking-related and non-smoking-related groups accordingly [16], calculated pooled RR and 95% CI in each group using a random-effects model, and then included the resulting pooled RR in the final meta-analysis.

We specifically evaluated the association between PD and melanoma, and other skin cancers. Cancers of other specific sites were included in this meta-analysis if there were more than 10 publications. Included were lung cancer, colorectal cancer, breast cancer, and prostate cancer.

For peer review only - http://bmjopen.bmj.com/site/about/guidelines.xhtml

Results

In total, we included 63 publications in this meta-analysis (Figure 1) [12 13 17-77]. Characteristics of all publications are listed in Supplementary table 1.

PD and total cancer

Combining 33 publications [12 13 18 27-32 35-39 41 50-52 54 57-62 64 65 69-71 73 75 76], pooled RR for association between PD and cancer was 0.82 (95% CI: 0.76, 0.88; p <0.001; Figure 2). We did not observe evidence for existence of publication bias (Egger test p = 0.27; supplementary figure 1). After stratified by temporal sequence, PD was significantly associated with a lower future risk of cancer (n = 21, pooled RR = 0.85; 95% CI: 0.76, 0.95; p = 0.004), and similar association was observed for cancer with a lower future risk of PD (n = 11, pooled RR = 0.74; 95% CI: 0.65, 0.85; p = <0.001). The significant inverse association persisted after further including meeting abstracts, excluding mortality studies, and excluding self-report outcomes that were not validated (table 1). Meta regression did not find significant difference between subgroups stratified by age (<69.3 years vs ≥69.3 years; mean value of the included studies), sex (men- vs women-dominant cohorts), ethnicity (Caucasian vs Asian), study design (cohort vs others), study quality (scored <7 vs ≥ 7), or year of study (before 2010, or 2010 and after, supplementary table 2).

We found 4 publications that examined the risk of cancers associated with the treatment of levodopa in PD patients (supplementary table 3) [18 24 31 56]. Although there was a significant lower risk of cancer after levodopa treatment or with higher cumulative levodopa treatment (pooled RR = 0.75; 95% CI: 0.61, 0.92; p = 0.007; supplementary figure 2a), Egger test (p =

For peer review only - http://bmjopen.bmj.com/site/about/guidelines.xhtml
For peer review only

0.005) and funnel plot (supplementary figure 2b) showed a significant publication bias and thus a potentially over-estimated result.

Smoking- and non-smoking-related cancers

Combining 21 publications [12 18 28 30-32 35 37 38 50 51 54 57-60 66 70 71 73 76], the pooled RR for association between PD and smoking-related cancers was 0.76 (95% CI: 0.67, 0.85; p <0.001; figure 3a). PD was also inversely associated with non-smoking-related cancers (n = 19; pooled RR = 0.92; 95% CI: 0.84, 0.99; p = 0.03; figure 3b) [12 18 25 28 30 31 35 37 38 50 51 57-59 66 70 71 73 76]. No publication bias was observed for both analyses (Egger test p = 0.45 and 0.50, respectively; supplementary figure 3).

Melanoma and non-melanoma skin cancer

Combining 29 publications [17 18 20 23 24 26 30 32 35 37 38 43 47 50 51 54 56 57 59 60 64 68-71 73 76-78], the pooled RR for association between PD and melanoma was 1.75 (95% CI: 1.43, 2.14; p <0.001; figure 4a). No publication bias was observed (Egger test p = 0.28; supplementary figure 4a). We did not find a statistically significant association between PD and non-melanoma skin cancer (n = 17; pooled RR = 0.90; 95% CI: 0.60, 1.34; p = 0.60; figure 4b) [31 32 34 35 37 41 47 50 54 56 57 67 69 71 73 76 77]. Egger test suggested no publication bias (p = 0.53), but funnel plot suggested potential over-estimation by small studies (supplementary figure 4b).

Other site-specific cancers

Lung cancer and colorectal cancer, two major cancers in the smoking-related category, both showed a significant inverse association with PD. There was no significant association between PD and breast cancer and prostate cancer (Table 1).

For peer review only - http://bmjopen.bmj.com/site/about/guidelines.xhtml

Discussion

In this meta-analysis of 63 publications and 17,994,584 participants, a significant inverse association between PD and total cancer was observed, with an 18% lower risk on both sides. Individuals with PD had a 15% lower risk of developing cancer, and vice versa, individuals with cancer had a 26% lower risk of developing PD. The inverse association was stronger for smoking-related cancers, compared to non-smoking-related cancers, though both achieved statistical significance. In contrast, PD was significantly associated with a 75% higher risk of melanoma. The overall inverse association is consistent with two published meta-analyses on this topic, which reported a 27% and 6% significantly lower risk for total cancer, respectively [10 79]. Relative to these two published meta-analyses, our study included a large number of studies and participants. The latest meta-analysis, for example, included 15 studies and 1,480,239 participants for examining the association between PD and total cancer [10 79]. In addition, this study did not stratify smoking-related and non-smoking-related cancers despite the analysis of associations between PD and specific cancers. Further, these two meta-analyses included both PD and parkinsonism [10 79].

One of the possible explanations for the inverse association between PD and total cancer is smoking. Smoking has been consistently associated with a low risk of PD and a high risk of many types of cancer [7]. Moreover, there is evidence that PD patients are less likely to be smokers [80]. Of note, only 10 out of the 32 publications included were adjusted for smoking behavior for total cancer risk in their original analysis [18 25 27-31 51 64 75], which may introduce residual confounding for the observed association between PD and total cancer. However, we also found that non-smoking-related cancer was inversely associated with PD, even when melanoma was included. Because only 4 publications separately reported risk estimates for total

For peer review only - http://bmjopen.bmj.com/site/about/guidelines.xhtml

cancer or non-smoking-related cancers after excluding melanoma[25 54 55 76], we did not perform a meta-analysis in these secondary categories. Our findings suggest that smoking is unlikely the only factor contributing to the observed inverse relation between PD and total cancer. Future studies with carefully adjusted smoking habits or environmental smoking exposure are warranted to better address this issue.

Our results, in line with the previous meta-analysis [10 79], suggest an inverse comorbidity between PD and cancer. The biological bases underlying the association is far from clear. Dysregulated cellular processes including those involved in the regulation of cell cycle, mitochondrial function, DNA repair, cell metabolism, and immune responses have been implicated in degeneration of neurons and tumorigenesis in dividing cells, often in the opposite directions. Cell proliferation and survival signals such as Wnt, P53, and PI3K/AKT may be upregulated in cancer and downregulated in neurodegeneration. The ubiquitin proteasome pathway of protein degradation on the other hand may be downregulated in neurodegeneration and upregulated in cancer [81-83]. Understanding the biological pathways would further facilitate investigations on potential strategies for better prevention, surveillance, and treatment of both PD and cancer.

Several common gene mutations have been implicated in PD and cancer [84]. *PARK2* was found to be a potent tumor suppressor gene [85 86] . Other PD-related genes *PINK1*, *PARK7*, and *LRRK2* have also been linked to cancer [60 87 88] . PD patients carrying *LRRK2* G2019S mutation have been associated with an overall increased risk of cancer, especially for hormone-related cancer and breast cancer [85], and most recently, lukemia, colon cancer, and skin cancer when compared with noncarrier PD [89]. Another PD-related *LRRK2* mutation R1441G was found to be associated with higher prevalence of hematological cancers [90]. Both G2019S and R1441G show increased LRRK2 kinase activity[91]. However, a recent study demonstrated that loss of *LRRK2* could

For peer review only - http://bmjopen.bmj.com/site/about/guidelines.xhtml

promote lung cancer development, adding to the complexicity of LRRK2-cancer link [92]. We found that only 14 of the included studies specifically identified idiopathic PD and excluded genetically conditioned PD. This limits our systematic review to fully synthesize the potential genetic overlaps between PD and cancer.

Although similarly characterized pathologically by over proliferation, different cancers are highly heterogeneous. While it remains to be determined whether the general inverse association exists across cancers of different sites and evolutionary origins, we and others have consistently shown that it did not apply to melanoma [9 93]. In this meta-analysis, we replicated the well-documented positive link between PD and melanoma. It has long been proposed that levodopa as the mainstay therapy for PD and a common precursor for both dopamine and melanin may contribute to the higher risk of melanoma in PD [94 95] . In this meta-analysis, we found a 37% higher risk of newly-developed PD after diagnosis of melanoma, suggesting that the observed PD-melanoma association may not be fully explained by the role of levodopa, if any [96]. Previously, we reported that the risk of incident PD is higher in people with a family history of melanoma among their first-degree relatives [93]. One plausible biological explanation of the association is the regulation of pigmentation by the *MC1R* gene, which presents and functions in both melanocytes and dopaminergic neurons [97 98] . Other genetic mutations, such as *CYP2D6* polymorphism and *VDR* polymorphism, might also be involved in both conditions [99-102] .

Despite all our effort in synthesizing all epidemiological evidence, the intrinsic limitations of meta-analysis cannot be avoided. First, studies included in this analysis came from diverse populations, with diverse designs and treatment strategies. They varied across assessments, statistical methods, and adjusted covariates. Although meta-regression did not find differences in age, sex, ethnicity, study design, and study quality, the highly heterogeneous nature of this meta-

For peer review only - http://bmjopen.bmj.com/site/about/guidelines.xhtml

analysis limits its interpretation into robust conclusions. Second, due to lack of access to original data, we could not adjust uniformly for confounders. We addressed this shortcoming by stratifying cancers into smoking-related or non-smoking-related cancers. However, there may be residual confounding since only a few studies adjusted for family history of PD/cancer, use of medications, sun exposure, duration of PD/cancer, use of medical care, or diet (eg, caffeine consumption) [103-105]. Third, many large-scale studies included in this meta-analysis used local/national registry databases, with disease diagnosis mostly based on International Classification of Disease codes. Notification to registries might not be complete, therefore the cases might be under-reported. Moreover, diagnosis criteria may slightly vary in different countries, hospitals, etc. Thus it is challenging to confirm and validate the information from these datasets. Lastly, all publications included in this meta-analysis were based on populations from North America, Europe, Australia, and Central and East Asia; No study has examined the association of PD and cancer in less-developed regions such as Africa, Southeast Asia, or South America. This could be due to difficulties in disease diagnosis and registry in these regions. Recent findings suggested positive associations between PD and most cancers in an East Asian population, highlighting possible discrepancies among different populations with different ethnic backgrounds [50 88]. Future studies should address the potentially important role of race/ethnicity and social-economic status.

We reviewed the current epidemiological evidence for the association between cancer and PD, with a meta-analysis of over 17 million individuals. We found that PD was associated with low risk of total cancer, except for melanoma, with which a positive association was identified. Despite the limitations, our study provided an overall picture of the association between the two

For peer review only - http://bmjopen.bmj.com/site/about/guidelines.xhtml

major disease entities. Future studies should aim to better understand the links between these two major chronic disease entities using epidemiological, clinical, and biological approaches.

Funding: This work was supported by the National Institutes of Health [R01NS102735], the Parkinson's Disease Foundation [PF-APDA-SFW-1914], the Jane & Alan Batkin Foundation [N/A], the Farmer Family Foundation [N/A], and the Michael J. Fox Foundation and the Aligning Science Across Parkinson's Initiative (ASAP-000312). None of the listed funders had a role in the design and conduct of the study; collection, management, analysis, and interpretation of the data; preparation, review, or approval of the manuscript; and decision to submit the manuscript for publication.

Data Availability: The data used to support the findings of this article are included within the article and the supplementary material.

Patient and Public Involvement Statement: It was not appropriate to involve patients or the public in the design, or conduct, or reporting, or dissemination plans of our research.

Ethics Statement: N/A

Competing Interests: The authors declare no conflicts of interest.

Acknowledgements: We thank Christina Wissinger, PhD, from Health Sciences Liaison Librarian of Penn State, who helped with search strategy. No financial compensation was made.

Author Contributions

Name	Location	Contribution

For peer review only - http://bmjopen.bmj.com/site/about/guidelines.xhtml

Xinyuan Zhang, BSc	The Pennsylvania State University	Concept and design; Acquisition, analysis, or interpretation of data; Statistical analysis; Drafting of the manuscript; revised the manuscript for intellectual content
David Guarin	Massachusetts General Hospital	Concept and design; Acquisition, analysis, or interpretation of data; revised the manuscript for intellectual content
**Niyaz Mohammadzadehhonarvar**	Massachusetts General Hospital	Acquisition, analysis, or interpretation of data; revised the manuscript for intellectual content
Xiqun Chen, MD, PhD	Massachusetts General Hospital	Concept and design; Acquisition, analysis, or interpretation of data; Drafting of the manuscript; revised the manuscript for intellectual content
Xiang Gao, MD, PhD	The Pennsylvania State University	Concept and design; Acquisition, analysis, or interpretation of data; Drafting of the manuscript; revised the manuscript for intellectual content

For peer review only

References

1. Zafar S, Yaddanapudi SS. Parkinson Disease. StatPearls. Treasure Island (FL): StatPearls Publishing Copyright © 2021, StatPearls Publishing LLC. 2021.

For peer review only - http://bmjopen.bmj.com/site/about/guidelines.xhtml

2. Mappin-Kasirer B, Pan H, Lewington S, et al. Tobacco smoking and the risk of Parkinson disease: A 65-year follow-up of 30,000 male British doctors. *Neurology* 2020;94(20):e2132-e38.
3. Lesage S, Brice A. Parkinson's disease: from monogenic forms to genetic susceptibility factors. *Hum Mol Genet* 2009;18(R1):R48-59. doi: 10.1093/hmg/ddp012 [published Online First: 2009/03/20]
4. Ferlay J, Colombet M, Soerjomataram I, et al. Estimating the global cancer incidence and mortality in 2018: GLOBOCAN sources and methods. *International journal of cancer* 2019;144(8):1941-53.
5. Ejma M, Madetko N, Brzecka A, et al. The Links between Parkinson's Disease and Cancer. *Biomedicines* 2020;8(10) doi: 10.3390/biomedicines8100416 [published Online First: 2020/10/18]
6. Kendal WS. Dying with cancer: the influence of age, comorbidity, and cancer site. *Cancer* 2008;112(6):1354-62. doi: 10.1002/cncr.23315 [published Online First: 2008/02/21]
7. Mappin-Kasirer B, Pan H, Lewington S, et al. Tobacco smoking and the risk of Parkinson disease. *Neurology* 2020;94(20):e2132. doi: 10.1212/WNL.0000000000009437
8. Feng DD, Cai W, Chen X. The associations between Parkinson's disease and cancer: the plot thickens. *Translational neurodegeneration* 2015;4:20-20. doi: 10.1186/s40035-015-0043-z
9. Huang P, Yang XD, Chen SD, et al. The association between Parkinson's disease and melanoma: a systematic review and meta-analysis. *Transl Neurodegener* 2015;4:21. doi: 10.1186/s40035-015-0044-y [published Online First: 2015/11/05]
10. Bajaj A, Driver JA, Schernhammer ES. Parkinson's disease and cancer risk: a systematic review and meta-analysis. *Cancer Causes Control* 2010;21(5):697-707. doi: 10.1007/s10552-009-9497-6 [published Online First: 2010/01/08]
11. Walker E, Hernandez AV, Kattan MW. Meta-analysis: Its strengths and limitations. *Cleve Clin J Med* 2008;75(6):431-9. doi: 10.3949/ccjm.75.6.431 [published Online First: 2008/07/04]
12. Ben-Shlomo Y, Marmot M. Survival and cause of death in a cohort of patients with parkinsonism: possible clues to aetiology? *Journal of Neurology, Neurosurgery & Psychiatry* 1995;58(3):293-99.
13. Hely MA, Morris JG, Traficante R, et al. The sydney multicentre study of Parkinson's disease: progression and mortality at 10 years. *Journal of neurology, neurosurgery, and psychiatry* 1999;67(3):300-07. doi: 10.1136/jnnp.67.3.300
14. Higgins JP, Thompson SG, Deeks JJ, et al. Measuring inconsistency in meta-analyses. *Bmj* 2003;327(7414):557-60.
15. Wells GA, Shea B, O'Connell Da, et al. The Newcastle-Ottawa Scale (NOS) for assessing the quality of nonrandomised studies in meta-analyses: Oxford, 2000.
16. Lushniak BD, Samet JM, Pechacek TF, et al. The health consequences of smoking—50 years of progress: A report of the surgeon general. 2014
17. Baade PD, Fritschi L, Freedman DM. Mortality due to amyotrophic lateral sclerosis and Parkinson's disease among melanoma patients. *Neuroepidemiology* 2007;28(1):16-20. doi: 10.1159/000097851 [published Online First: 2006/12/14]
18. Becker C, Brobert GP, Johansson S, et al. Cancer risk in association with Parkinson disease: a population-based study. *Parkinsonism Relat Disord* 2010;16(3):186-90. doi: 10.1016/j.parkreldis.2009.11.005 [published Online First: 2009/12/01]
19. Bermejo-Pareja F, Benito-Leon J, Trincado R, et al. Neurodegenerative Diseases (Alzheimer and Parkinson), but Not Vascular Dementia, Are Negatively Associated to Cancer as Cause of Death in the NEDICES Cohort (P01.086). *Neurology* 2012;78(Meeting Abstracts 1):P01.086-P01.86. doi: 10.1212/WNL.78.1_MeetingAbstracts.P01.086
20. Bertoni JM, Arlette JP, Fernandez HH, et al. Increased melanoma risk in Parkinson disease: a prospective clinicopathological study. *Arch Neurol* 2010;67(3):347-52. doi: 10.1001/archneurol.2010.1 [published Online First: 2010/03/10]

For peer review only - http://bmjopen.bmj.com/site/about/guidelines.xhtml

21. Binaghi E, Mascia MM, Solla P, et al. Correlation between Parkinson's disease and malignant tumors. *Movement Disorders* 2016;31:S631-S32.
22. Boursi B, Mamtani R, Haynes K, et al. Parkinson's disease and colorectal cancer risk-A nested case control study. *Cancer Epidemiol* 2016;43:9-14. doi: 10.1016/j.canep.2016.05.007 [published Online First: 2016/05/28]
23. Constantinescu R, Elm J, Auinger P, et al. Malignant melanoma in early-treated Parkinson's disease: the NET-PD trial. *Mov Disord* 2014;29(2):263-5. doi: 10.1002/mds.25734 [published Online First: 2013/12/11]
24. Constantinescu R, Romer M, Kieburtz K, et al. Malignant melanoma in early Parkinson's disease: the DATATOP trial. *Mov Disord* 2007;22(5):720-2. doi: 10.1002/mds.21273 [published Online First: 2007/03/22]
25. Cui X, Liew Z, Hansen J, et al. Cancers Preceding Parkinson's Disease after Adjustment for Bias in a Danish Population-Based Case-Control Study. *Neuroepidemiology* 2019;52(3-4):136-43. doi: 10.1159/000494292 [published Online First: 2019/01/21]
26. Dalvin LA, Damento GM, Yawn BP, et al. Parkinson Disease and Melanoma: Confirming and Reexamining an Association. *Mayo Clin Proc* 2017;92(7):1070-79. doi: 10.1016/j.mayocp.2017.03.014 [published Online First: 2017/07/10]
27. D'Amelio M, Ragonese P, Morgante L, et al. Tumor diagnosis preceding Parkinson's disease: a case-control study. *Mov Disord* 2004;19(7):807-11. doi: 10.1002/mds.20123 [published Online First: 2004/07/16]
28. Driver JA, Kurth T, Buring JE, et al. Prospective case-control study of nonfatal cancer preceding the diagnosis of Parkinson's disease. *Cancer Causes Control* 2007;18(7):705-11. doi: 10.1007/s10552-007-9005-9 [published Online First: 2007/06/15]
29. Driver JA, Kurth T, Buring JE, et al. Parkinson disease and risk of mortality: a prospective comorbidity-matched cohort study. *Neurology* 2008;70(16 Pt 2):1423-30. doi: 10.1212/01.wnl.0000310414.85144.ee [published Online First: 2008/04/17]
30. Driver JA, Logroscino G, Buring JE, et al. A prospective cohort study of cancer incidence following the diagnosis of Parkinson's disease. *Cancer Epidemiol Biomarkers Prev* 2007;16(6):1260-5. doi: 10.1158/1055-9965.EPI-07-0038 [published Online First: 2007/06/06]
31. Elbaz A, Peterson BJ, Bower JH, et al. Risk of cancer after the diagnosis of Parkinson's disease: a historical cohort study. *Mov Disord* 2005;20(6):719-25. doi: 10.1002/mds.20401 [published Online First: 2005/02/11]
32. Elbaz A, Peterson BJ, Yang P, et al. Nonfatal cancer preceding Parkinson's disease: a case-control study. *Epidemiology* 2002;13(2):157-64. doi: 10.1097/00001648-200203000-00010 [published Online First: 2002/03/07]
33. Fall PA, Saleh A, Fredrickson M, et al. Survival time, mortality, and cause of death in elderly patients with Parkinson's disease: a 9-year follow-up. *Mov Disord* 2003;18(11):1312-6. doi: 10.1002/mds.10537 [published Online First: 2003/11/26]
34. Ferreira J, Silva JM, Freire R, et al. Skin cancers and precancerous lesions in Parkinson's disease patients. *Mov Disord* 2007;22(10):1471-5. doi: 10.1002/mds.21575 [published Online First: 2007/05/23]
35. Fois AF, Wotton CJ, Yeates D, et al. Cancer in patients with motor neuron disease, multiple sclerosis and Parkinson's disease: record linkage studies. *J Neurol Neurosurg Psychiatry* 2010;81(2):215-21. doi: 10.1136/jnnp.2009.175463 [published Online First: 2009/09/04]
36. Freedman DM, Pfeiffer RM. Associations Between Parkinson Disease and Cancer in US Asian Americans. *Jama Oncology* 2016;2(8):1093-94. doi: 10.1001/jamaoncol.2016.0729

For peer review only - http://bmjopen.bmj.com/site/about/guidelines.xhtml

37. Freedman DM, Travis LB, Gridley G, et al. Amyotrophic lateral sclerosis mortality in 1.9 million US cancer survivors. *Neuroepidemiology* 2005;25(4):176-80. doi: 10.1159/000087447 [published Online First: 2005/08/17]
38. Freedman DM, Wu J, Chen H, et al. Associations between cancer and Parkinson's disease in U.S. elderly adults. *Int J Epidemiol* 2016;45(3):741-51. doi: 10.1093/ije/dyw016 [published Online First: 2016/03/19]
39. Gorell JM, Johnson CC, Rybicki BA. Parkinson's disease and its comorbid disorders: an analysis of Michigan mortality data, 1970 to 1990. *Neurology* 1994;44(10):1865-8. doi: 10.1212/wnl.44.10.1865 [published Online First: 1994/10/01]
40. Jamrozik Z, Janik P, Friedman A, et al. Incidence of prostate cancer in patients with Parkinson's disease. *European Journal of Neurology* 2005;12:98-98.
41. Jansson B, Jankovic J. Low cancer rates among patients with Parkinson's disease. *Ann Neurol* 1985;17(5):505-9. doi: 10.1002/ana.410170514 [published Online First: 1985/05/01]
42. Jespersen CG, Norgaard M, Borre M. Parkinson's disease and risk of prostate cancer: A Danish population-based case-control study, 1995-2010. *Cancer Epidemiol* 2016;45:157-61. doi: 10.1016/j.canep.2016.11.002 [published Online First: 2016/11/14]
43. Kareus SA, Figueroa KP, Cannon-Albright LA, et al. Shared predispositions of parkinsonism and cancer: a population-based pedigree-linked study. *Arch Neurol* 2012;69(12):1572-7. doi: 10.1001/archneurol.2012.2261 [published Online First: 2012/09/05]
44. Kelm RC, K. P, J. LS, et al. Melanoma and Parkinson disease: A large, urban, single-center, midwestern U.S. population study. *Journal of the American Academy of Dermatology* 2018;79(3) doi: 10.1016/j.jaad.2018.05.765
45. Lai SW, Liao KF, Lin CH, et al. Parkinson's disease and lung cancer: a population-based case-control study in Taiwan. *Geriatr Gerontol Int* 2013;13(1):238-40. doi: 10.1111/j.1447-0594.2012.00919.x [published Online First: 2013/01/05]
46. Lai SW, Lin CL, Liao KF, et al. Parkinson's disease and hepatocellular carcinoma in older people: A population-based case-control study in Taiwan. *International Medical Journal* 2015;22(4):313-14.
47. Lerman S, Amichai B, Weinstein G, et al. Parkinson's Disease, Melanoma, and Keratinocyte Carcinoma: A Population-Based Study. *Neuroepidemiology* 2018;50(3-4):168-73. doi: 10.1159/000487855 [published Online First: 2018/03/23]
48. Liao KF, Lin CL, Lai SW. Parkinson's disease and risk of colorectal cancer: A population-based case-control study in Taiwan. *Neurology Asia* 2017;22(2):133-38.
49. Liao KF, Lin CL, Lai SW, et al. Parkinson's disease and risk of pancreatic cancer: a population-based case-control study in Taiwan. *Neurology Asia* 2015;20(3):251-55.
50. Lin PY, Chang SN, Hsiao TH, et al. Association Between Parkinson Disease and Risk of Cancer in Taiwan. *JAMA Oncol* 2015;1(5):633-40. doi: 10.1001/jamaoncol.2015.1752 [published Online First: 2015/07/17]
51. Lo RY, Tanner CM, Van Den Eeden SK, et al. Comorbid cancer in Parkinson's disease. *Mov Disord* 2010;25(12):1809-17. doi: 10.1002/mds.23246 [published Online First: 2010/07/30]
52. Minami Y, Yamamoto R, Nishikouri M, et al. Mortality and cancer incidence in patients with Parkinson's disease. *J Neurol* 2000;247(6):429-34. doi: 10.1007/s004150070171 [published Online First: 2000/08/10]
53. Naghavi-Behzad M, Piri R. Risk of cancer among patients with Parkinson disease. *Parkinsonism Relat Disord* 2016;22:e34.

For peer review only - http://bmjopen.bmj.com/site/about/guidelines.xhtml

54. Olsen JH, Friis S, Frederiksen K. Malignant melanoma and other types of cancer preceding Parkinson disease. *Epidemiology* 2006;17(5):582-7. doi: 10.1097/01.ede.0000229445.90471.5e [published Online First: 2006/07/14]
55. Olsen JH, Friis S, Frederiksen K, et al. Atypical cancer pattern in patients with Parkinson's disease. *Br J Cancer* 2005;92(1):201-5. doi: 10.1038/sj.bjc.6602279 [published Online First: 2004/12/08]
56. Olsen JH, Tangerud K, Wermuth L, et al. Treatment with levodopa and risk for malignant melanoma. *Mov Disord* 2007;22(9):1252-7. doi: 10.1002/mds.21397 [published Online First: 2007/05/31]
57. Ong EL, Goldacre R, Goldacre M. Differential risks of cancer types in people with Parkinson's disease: a national record-linkage study. *Eur J Cancer* 2014;50(14):2456-62. doi: 10.1016/j.ejca.2014.06.018 [published Online First: 2014/07/30]
58. Park JH, Kim DH, Park YG, et al. Cancer risk in patients with Parkinson's disease in South Korea: A nationwide, population-based cohort study. *Eur J Cancer* 2019;117:5-13. doi: 10.1016/j.ejca.2019.04.033 [published Online First: 2019/06/24]
59. Peretz C, Gurel R, Rozani V, et al. Cancer incidence among Parkinson's disease patients in a 10-yrs time-window around disease onset: A large-scale cohort study. *Parkinsonism Relat Disord* 2016;28:68-72. doi: 10.1016/j.parkreldis.2016.04.028 [published Online First: 2016/05/11]
60. Agalliu I, Ortega RA, Luciano MS, et al. Cancer outcomes among Parkinson's disease patients with leucine rich repeat kinase 2 mutations, idiopathic Parkinson's disease patients, and nonaffected controls. *Mov Disord* 2019;34(9):1392-98. doi: 10.1002/mds.27807 [published Online First: 2019/07/28]
61. Ording AG, Veres K, Horváth-Puhó E, et al. Alzheimer's and Parkinson's Diseases and the Risk of Cancer: A Cohort Study. *J Alzheimers Dis* 2019;72(4):1269-77. doi: 10.3233/jad-190867 [published Online First: 2019/11/11]
62. Pinter B, Diem-Zangerl A, Wenning GK, et al. Mortality in Parkinson's disease: a 38-year follow-up study. *Mov Disord* 2015;30(2):266-9. doi: 10.1002/mds.26060 [published Online First: 2014/12/03]
63. Piri R, Naghavi-Behzad M. Association between cancers and risk of Parkinson disease: A cohort study. *Parkinsonism Relat Disord* 2016;22:e33.
64. Powers KM, Smith-Weller T, Franklin GM, et al. Diabetes, smoking, and other medical conditions in relation to Parkinson's disease risk. *Parkinsonism Relat Disord* 2006;12(3):185-9. doi: 10.1016/j.parkreldis.2005.09.004 [published Online First: 2005/12/21]
65. Pressley JC, Louis ED, Tang MX, et al. The impact of comorbid disease and injuries on resource use and expenditures in parkinsonism. *Neurology* 2003;60(1):87-93. doi: 10.1212/wnl.60.1.87 [published Online First: 2003/01/15]
66. Rugbjerg K, Friis S, Lassen CF, et al. Malignant melanoma, breast cancer and other cancers in patients with Parkinson's disease. *Int J Cancer* 2012;131(8):1904-11. doi: 10.1002/ijc.27443 [published Online First: 2012/01/27]
67. Ryu HJ, Park JH, Choi M, et al. Parkinson's disease and skin cancer risk: a nationwide population-based cohort study in Korea. *Journal of the European Academy of Dermatology and Venereology* 2020;34(12):2775-80.
68. Schwid SR, Bausch J, Oakes D, et al. Cancer incidence in a trial of an antiapoptotic agent for Parkinson's disease. *Mov Disord* 2010;25(12):1801-8. doi: 10.1002/mds.23006 [published Online First: 2010/07/30]
69. Shalaby SY, Louis ED. Increased Odds of Melanoma: Parkinson's Disease, Essential Tremor, Dystonia versus Controls. *Neuroepidemiology* 2016;46(2):128-36. doi: 10.1159/000443794 [published Online First: 2016/01/29]

For peer review only - http://bmjopen.bmj.com/site/about/guidelines.xhtml

70. Sun LM, Liang JA, Chang SN, et al. Analysis of Parkinson's disease and subsequent cancer risk in Taiwan: a nationwide population-based cohort study. *Neuroepidemiology* 2011;37(2):114-9. doi: 10.1159/000331489 [published Online First: 2011/10/12]
71. Tacik P, Curry S, Fujioka S, et al. Cancer in Parkinson's disease. *Parkinsonism Relat Disord* 2016;31:28-33. doi: 10.1016/j.parkreldis.2016.06.014 [published Online First: 2016/07/04]
72. Tang CF, Lu MK, Muo CH, et al. Increased risk of brain tumor in patients with Parkinson's disease: a nationwide cohort study in Taiwan. *Acta Neurol Scand* 2016;134(2):148-53. doi: 10.1111/ane.12524 [published Online First: 2015/10/29]
73. Vanacore N, Spila-Alegiani S, Raschetti R, et al. Mortality cancer risk in parkinsonian patients: a population-based study. *Neurology* 1999;52(2):395-8. doi: 10.1212/wnl.52.2.395 [published Online First: 1999/02/05]
74. Wing K, Douglas I, Bhaskaran K, et al. A Cohort Analysis of the Association between Parkinson's Disease and Cancer Using the UK General Practice Research Database. *Pharmacoepidemiology and Drug Safety* 2012;21:442-43.
75. Winter AC, Rist PM, Buring JE, et al. Prospective comorbidity-matched study of Parkinson's disease and risk of mortality among women. *Bmj Open* 2016;6(9):5. doi: 10.1136/bmjopen-2016-011888
76. Wirdefeldt K, Weibull CE, Chen H, et al. Parkinson's disease and cancer: A register-based family study. *Am J Epidemiol* 2014;179(1):85-94. doi: 10.1093/aje/kwt232 [published Online First: 2013/10/22]
77. Dinesh D, Lee JS, Gao X, et al. Skin conditions in early Parkinson's disease. *Parkinsonism & Related Disorders* 2021;84:40-46. doi: https://doi.org/10.1016/j.parkreldis.2021.01.018
78. Ryu HJ, Park JH, Choi M, et al. Parkinson's disease and skin cancer risk: a nationwide population-based cohort study in Korea. *J Eur Acad Dermatol Venereol* 2020;34(12):2775-80. doi: 10.1111/jdv.16462 [published Online First: 2020/04/15]
79. Zhang P, Liu B. Association between Parkinson's Disease and Risk of Cancer: A PRISMA-compliant Meta-analysis. *ACS Chem Neurosci* 2019;10(10):4430-39. doi: 10.1021/acschemneuro.9b00498 [published Online First: 2019/10/05]
80. Larsson SC, Carter P, Kar S, et al. Smoking, alcohol consumption, and cancer: A mendelian randomisation study in UK Biobank and international genetic consortia participants. *PLoS Med* 2020;17(7):e1003178. doi: 10.1371/journal.pmed.1003178 [published Online First: 2020/07/24]
81. Driver JA. Inverse association between cancer and neurodegenerative disease: review of the epidemiologic and biological evidence. *Biogerontology* 2014;15(6):547-57. doi: 10.1007/s10522-014-9523-2 [published Online First: 2014/08/13]
82. Ibanez K, Boullosa C, Tabares-Seisdedos R, et al. Molecular evidence for the inverse comorbidity between central nervous system disorders and cancers detected by transcriptomic meta-analyses. *PLoS Genet* 2014;10(2):e1004173. doi: 10.1371/journal.pgen.1004173 [published Online First: 2014/03/04]
83. Klus P, Cirillo D, Botta Orfila T, et al. Neurodegeneration and Cancer: Where the Disorder Prevails. *Sci Rep* 2015;5:15390. doi: 10.1038/srep15390 [published Online First: 2015/10/27]
84. Dube U, Ibanez L, Budde JP, et al. Overlapping genetic architecture between Parkinson disease and melanoma. *Acta Neuropathol* 2020;139(2):347-64. doi: 10.1007/s00401-019-02110-z [published Online First: 2019/12/18]
85. Gong Y, Zack TI, Morris LG, et al. Pan-cancer genetic analysis identifies PARK2 as a master regulator of G1/S cyclins. *Nat Genet* 2014;46(6):588-94. doi: 10.1038/ng.2981 [published Online First: 2014/05/06]

For peer review only - http://bmjopen.bmj.com/site/about/guidelines.xhtml

86. Gupta A, Anjomani-Virmouni S, Koundouros N, et al. PARK2 Depletion Connects Energy and Oxidative Stress to PI3K/Akt Activation via PTEN S-Nitrosylation. *Mol Cell* 2017;65(6):999-1013.e7. doi: 10.1016/j.molcel.2017.02.019 [published Online First: 2017/03/18]
87. Agalliu I, San Luciano M, Mirelman A, et al. Higher frequency of certain cancers in LRRK2 G2019S mutation carriers with Parkinson disease: a pooled analysis. *JAMA Neurol* 2015;72(1):58-65. doi: 10.1001/jamaneurol.2014.1973 [published Online First: 2014/11/18]
88. Feng DD, Cai W, Chen X. The associations between Parkinson's disease and cancer: the plot thickens. *Transl Neurodegener* 2015;4:20. doi: 10.1186/s40035-015-0043-z [published Online First: 2015/10/28]
89. Shu L, Zhang Y, Pan H, et al. Clinical Heterogeneity Among LRRK2 Variants in Parkinson's Disease: A Meta-Analysis. *Frontiers in Aging Neuroscience* 2018;10(283) doi: 10.3389/fnagi.2018.00283
90. Ruiz-Martínez J, de la Riva P, Rodríguez-Oroz MC, et al. Prevalence of cancer in Parkinson's disease related to R1441G and G2019S mutations in LRRK2. *Mov Disord* 2014;29(6):750-5. doi: 10.1002/mds.25778 [published Online First: 2013/12/21]
91. Ysselstein D, Nguyen M, Young TJ, et al. LRRK2 kinase activity regulates lysosomal glucocerebrosidase in neurons derived from Parkinson's disease patients. *Nature communications* 2019;10(1):5570. doi: 10.1038/s41467-019-13413-w [published Online First: 2019/12/06]
92. Lebovitz C, Wretham N, Osooly M, et al. Loss of Parkinson's susceptibility gene LRRK2 promotes carcinogen-induced lung tumorigenesis. *Scientific Reports* 2021;11(1):2097. doi: 10.1038/s41598-021-81639-0
93. Gao X, Simon KC, Han J, et al. Family history of melanoma and Parkinson disease risk. *Neurology* 2009;73(16):1286-91. doi: 10.1212/WNL.0b013e3181bd13a1 [published Online First: 2009/10/21]
94. Weissenrieder JS, Neighbors JD, Mailman RB, et al. Cancer and the Dopamine D(2) Receptor: A Pharmacological Perspective. *J Pharmacol Exp Ther* 2019;370(1):111-26. doi: 10.1124/jpet.119.256818 [published Online First: 2019/04/20]
95. Siple JF, Schneider DC, Wanlass WA, et al. Levodopa therapy and the risk of malignant melanoma. *Ann Pharmacother* 2000;34(3):382-5. doi: 10.1345/aph.19150 [published Online First: 2000/08/05]
96. Bose A, Petsko GA, Eliezer D. Parkinson's Disease and Melanoma: Co-Occurrence and Mechanisms. *J Parkinsons Dis* 2018;8(3):385-98. doi: 10.3233/JPD-171263 [published Online First: 2018/07/12]
97. Chen X, Feng D, Schwarzschild MA, et al. Red hair, MC1R variants, and risk for Parkinson's disease - a meta-analysis. *Ann Clin Transl Neurol* 2017;4(3):212-16. doi: 10.1002/acn3.381 [published Online First: 2017/03/10]
98. Gao X, Simon KC, Han J, et al. Genetic determinants of hair color and Parkinson's disease risk. *Ann Neurol* 2009;65(1):76-82. doi: 10.1002/ana.21535 [published Online First: 2009/02/06]
99. Fullard ME, Duda JE. A Review of the Relationship Between Vitamin D and Parkinson Disease Symptoms. *Frontiers in neurology* 2020;11:454-54. doi: 10.3389/fneur.2020.00454
100. Campbell MJ, Trump DL. Vitamin D Receptor Signaling and Cancer. *Endocrinology and metabolism clinics of North America* 2017;46(4):1009-38. doi: 10.1016/j.ecl.2017.07.007 [published Online First: 2017/09/29]
101. Chan CWH, Law BMH, So WKW, et al. Pharmacogenomics of breast cancer: highlighting CYP2D6 and tamoxifen. *J Cancer Res Clin Oncol* 2020;146(6):1395-404. doi: 10.1007/s00432-020-03206-w [published Online First: 2020/04/10]

For peer review only - http://bmjopen.bmj.com/site/about/guidelines.xhtml

102. Ur Rasheed MS, Mishra AK, Singh MP. Cytochrome P450 2D6 and Parkinson's Disease: Polymorphism, Metabolic Role, Risk and Protection. *Neurochem Res* 2017;42(12):3353-61. doi: 10.1007/s11064-017-2384-8 [published Online First: 2017/09/06]
103. Schrempf M, Haluza D, Simic S, et al. Is Multidirectional UV Exposure Responsible for Increasing Melanoma Prevalence with Altitude? A Hypothesis Based on Calculations with a 3D-Human Exposure Model. *International journal of environmental research and public health* 2016;13(10):961. doi: 10.3390/ijerph13100961
104. Gross A, Racette BA, Camacho-Soto A, et al. Use of medical care biases associations between Parkinson disease and other medical conditions. *Neurology* 2018;90(24):e2155-e65.
105. Mahajan A, Chirra M, Dwivedi AK, et al. Skin cancer may delay onset but not progression of Parkinson's disease: a nested case-control study. *Frontiers in neurology* 2020;11:406.

Table 1. Association between Parkinson disease and cancer.

	No. of publications	Pooled RR (95% CI)	P for significance	P for heterogeneity
**Total cancer**				
All full-text publications	33	0.82 (0.76, 0.88)	<0.001	<0.001
Including abstracts	37	0.80 (0.74, 0.86)	<0.001	<0.001
Excluding mortality studies	25	0.85 (0.79, 0.92)	<0.001	<0.001
Excluding self-report diagnosis	31	0.81 (0.75, 0.87)	<0.001	<0.001
**Smoking-related cancer[1]**	21	0.76 (0.67, 0.85)	<0.001	<0.001
**Non-smoking-related cancer[2]**	19	0.92 (0.84, 0.99)	0.03	<0.001
**Site-specific cancer**				
Melanoma	29	1.75 (1.43, 2.14)	<0.001	<0.001
Non-melanoma skin cancer	17	0.90 (0.60, 1.34)	0.60	<0.001
Lung cancer	20	0.62 (0.51, 0.75)	<0.001	<0.001
Colorectal cancer	20	0.82 (0.75, 0.90)	<0.001	<0.001
Breast cancer	15	1.02 (0.93, 1.12)	0.66	0.001
Prostate cancer	17	0.93 (0.83, 1.03)	0.18	<0.001

For peer review only - http://bmjopen.bmj.com/site/about/guidelines.xhtml

[1]Smoking-related cancer includes cancer of the lung, larynx, mouth, esophagus, throat, bladder, kidney, liver, stomach, pancreas, colon and rectum, and cervix, as well as acute myeloid leukemia;

[2]Non-smoking-related cancer includes all other cancer except for those listed as smoking-related;

RR, relative risk; CI, confidence interval.

Figure 1. Flow chart.

Figure 2. Association between Parkinson's disease and total cancer in 33 publications. The figure shows the estimates (ESs) and 95% confidence intervals (CIs) for each study and the pooled result from random-effects model. Studies are stratified by temporal relationship of Parkinson's disease and cancer. M: men; W: women.

Figure 3. Association between Parkinson's disease and (A) smoking-related cancers in 21 publications, and (B) non-smoking-related cancers in 19 publications. Figure shows the estimates (ESs) and 95% confidence intervals (CIs) for each study and the pooled result from random effects model. Studies are stratified by temporal relationship of Parkinson's disease and cancer. M: men; W: women; *: pooled risk estimates calculated from individual ES in original publication.

Figure 4. Association between Parkinson's disease and (A) melanoma in 29 publications, and (B) non-melanoma skin cancers in 17 publications. Figure shows the estimates (ESs) and 95% confidence intervals (CIs) for each study and the pooled result from random effects model. Studies are stratified by temporal relationship of Parkinson's disease and cancer. M: men; W: women; BCC: basal cell carcinoma; SCC: squamous cell carcinoma; y: years of age.

For peer review only

For peer review only - http://bmjopen.bmj.com/site/about/guidelines.xhtml

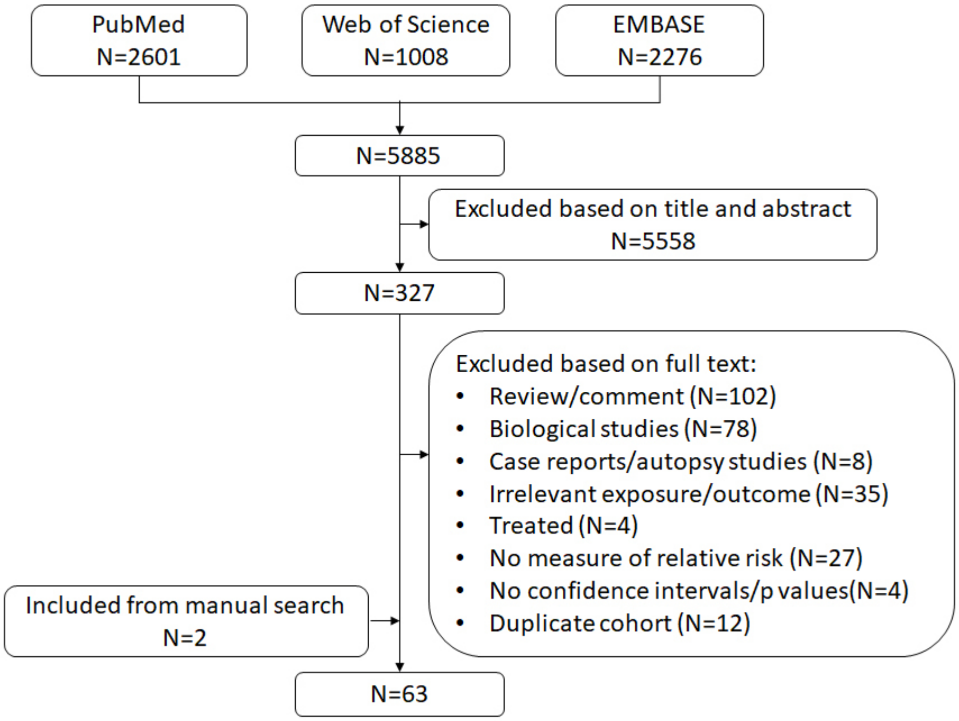

Flow chart.

314x242mm (72 x 72 DPI)

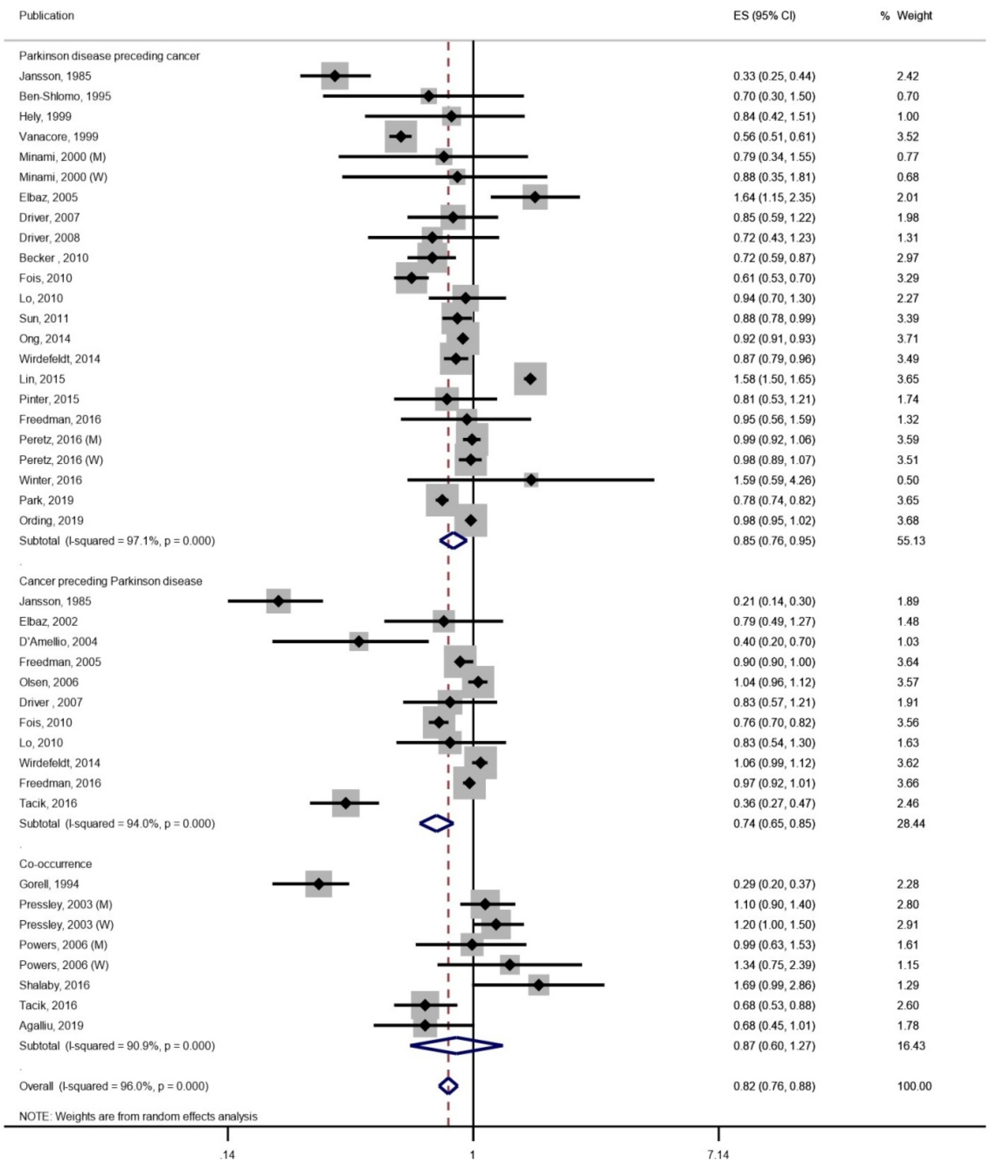

Figure 2. Association between Parkinson's disease and total cancer in 33 publications. The figure shows the estimates (ESs) and 95% confidence intervals (CIs) for each study and the pooled result from random-effects model. Studies are stratified by temporal relationship of Parkinson's disease and cancer. M: men; W: women.

239x283mm (150 x 150 DPI)

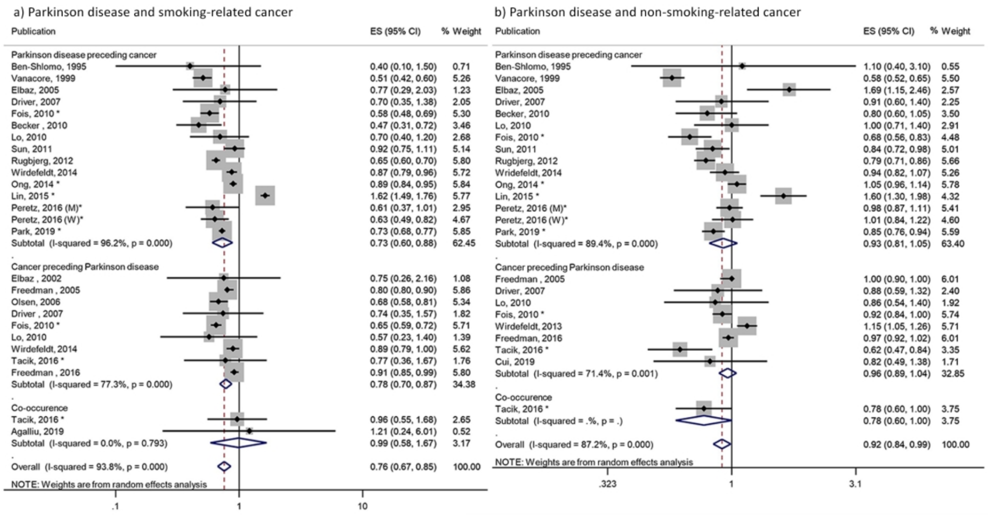

Figure 3. Association between Parkinson's disease and (A) smoking-related cancers in 21 publications, and (B) non-smoking-related cancers in 19 publications. Figure shows the estimates (ESs) and 95% confidence intervals (CIs) for each study and the pooled result from random effects model. Studies are stratified by temporal relationship of Parkinson's disease and cancer. M: men; W: women; *: pooled risk estimates calculated from individual ES in original publication.

763x406mm (72 x 72 DPI)

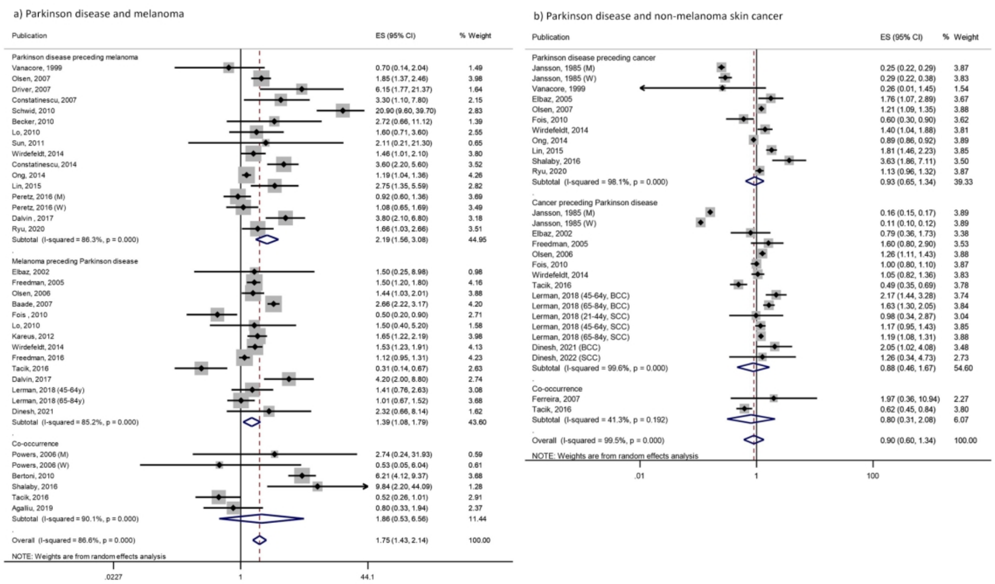

Association between Parkinson's disease and (A) melanoma in 29 publications, and (B) non-melanoma skin cancers in 17 publications. Figure shows the estimates (ESs) and 95% confidence intervals (CIs) for each study and the pooled result from random effects model. Studies are stratified by temporal relationship of Parkinson's disease and cancer. M: men; W: women; BCC: basal cell carcinoma; SCC: squamous cell carcinoma; y: years of age.

737x427mm (72 x 72 DPI)

Supplementary material for "Parkinson Disease and cancer: a systematic review and meta-analysis of 17,697,252 participants"

Content

Supplementary methods

Search strategy

Duplicate database inclusion/exclusion

Supplementary tables

Table 1. Characteristics of publications included in meta-analysis of Parkinson Disease and cancer.

Table 2. Meta regression and sub-group analysis on association between Parkinson disease and total cancer.

Table 3. Publications on risk of total cancer associated with levodopa treatment.

Supplementary figures

Figure 1. Funnel plot of studies of the association between Parkinson Disease and total cancer.

Figure 2. Association between use of levodopa and risk of total cancer in 4 publications.

Figure 3. Funnel plot of studies of the association between Parkinson Disease and a) smoking-related cancers, b) non-smoking-related cancers.

Figure 4. Funnel plot of studies of the association between Parkinson Disease and a) melanoma, b) non-melanoma skin cancers.

For peer review only - http://bmjopen.bmj.com/site/about/guidelines.xhtml

Supplementary methods

PubMed search strategy for Parkinson Disease and cancer

(("Parkinson Disease"[Mesh] OR "Parkinson Disease"[TW] OR "Parkinson's Disease"[TW] OR "Parkinsonism"[TW]) AND "cancer"[sb] AND ("Epidemiologic Studies"[Mesh] OR "Epidemiologic"[TW] OR "epidemiological"[TW] OR "Case-Control Studies"[Mesh] OR "case-control"[TW] OR "case control"[TW] OR "Case-Comparison"[TW] OR "Case Comparison"[TW] OR "Case-Compeer"[TW] OR "Case-Referent"[TW] OR "Case Referent"[TW] OR "Case-Base"[TW] OR "Case Base"[TW] OR "Cohort Studies"[Mesh] OR "cohort"[TW] OR "Concurrent"[TW] OR "Incidence"[TW] OR "Cross-Sectional Studies"[Mesh] OR "cross-sectional"[TW] OR "cross sectional"[TW] OR "Disease Frequency"[TW] OR "Prevalence"[TW] OR "Follow-Up Studies"[Mesh] OR "Follow-Up"[TW] OR "Follow Up"[TW] OR "Followup"[TW] OR "Longitudinal Studies"[Mesh] OR "longitudinal"[TW] OR "Retrospective Studies"[Mesh] OR "retrospective"[TW] OR "Prospective Studies"[Mesh] OR "prospective"[TW] OR "observational"[TW] OR "Observational Study" [Publication Type] OR "mortality studies"[TW] OR "ratio"[TW] OR "risk"[TW]) AND English[lang]) NOT ("animals"[MeSH Terms] NOT ("humans"[MeSH Terms] AND "animals"[MeSH Terms]))

Selection of publications that used same population

Leibson 2006, Elbaz 2002, Elbaz 2005, and Dalvin 2017 all used data from Mayo Clinic, Minnesota. Leibson 2006 was updated by Elbaz 2002 and 2005, therefore excluded from this meta-analysis. Elbaz 2002 studied PD risk after cancer, while Elbaz 2005 studied cancer risk after PD, therefore both publications were included. Dalvin 2017 was a cross-sectional extension of previous result, but it contained detailed analysis on melanoma, therefore it was not included in the analysis for total cancer, but was included for melanoma.

Olsen 2005, Olsen 2006, Olsen 2007, Rugbjerg 2012, Frandson 2014, Jespersen 2016, Cui 2019, and Ording 2019 all used National Hospital Register of Denmark. Frandson 2014 was a cross-sectional study that overlapped with Olsen 2006, Rugbjerg 2012, and Ording 2019, therefore was not included in this meta-analysis. Other publications varied in designs, time windows, temporal relationship, and cancer of interest.

For peer review only - http://bmjopen.bmj.com/site/about/guidelines.xhtml

There were also multiple publications from Physicians' Health Study, Women's Health Study, and Taiwan Health Registry. However, all of these groups of paper varied in designs, time windows, temporal relationship, and cancer of interest, therefore were not considered as duplicates. Other duplicates were meeting proceedings/abstracts of later-published articles, or duplicates that were not identified by matching in Endnote X9.

For peer review only

For peer review only - http://bmjopen.bmj.com/site/about/guidelines.xhtml

Supplementary tables

Table 1. Characteristics of publications included in meta-analysis of Parkinson Disease (PD) and cancer.

**Author**	**Year**	**Publication type**	**Temporal direction**	**Study design**	**Location**	**Cohort**	**Case N**	**Control N**	**Follow time, y**	**Disease ascertainment**	**Smoking adjusted**	**Quality score**
Agalliu	2019	Article	Co-occurrence	Cross-sectional	Europe, Israel, and the United States	Michael J. Fox Foundation	712	218	\	Diagnosed; idiopathic PD	No	5
Baade	2007	Article	Cancer preceding PD	Case-only cohort	Australia		127037		6.0	Coded	No	5
Becker	2010	Article	PD preceding cancer	1. Matched cohort 2. Case-control	UK	UK-based General Practice Research Database	466	1864	\	Validated; idiopathic PD	Yes	9
Ben-Shlomo	1995	Article	PD preceding cancer	Matched cohort	England and Wales	Second National Morbidity Study	220	421	\	Coded	No	7
Bermejo-Pareja	2012	Abstract	PD preceding cancer	Prospective cohort	Spain	Neurologic Disorders in Central Spain (NEDICES)	81	5197	\	\	No	\
Bertoni	2010	Article	Co-occurence	Case-only cohort	North America		2106		\	Diagnosed	No	7
Binagh	2016	Abstract	Co-occurence	Cross-sectional	Italy		529		\	Diagnosed	Yes	\
Boursi	2016	Article	PD preceding cancer	Case-control	UK	The Health Improvement Network	22093	85833	\	Diagnosed	Yes	9
Constatinescu	2007	Article	PD preceding cancer	Case-only cohort	North America	DATATOP	800		4.61	Diagnosed; idiopathic PD	No	4
Constatinescu	2014	Article	PD preceding cancer	Case-only cohort	US	NET-PD	1737		3.71	Diagnosed; idiopathic PD	No	4

For peer review only - http://bmjopen.bmj.com/site/about/guidelines.xhtml

Author	Year	Publication type	Temporal direction	Study design	Location	Cohort	Case N	Control N	Follow time, y	Disease ascertainment	Smoking adjusted	Quality score
Cui	2019	Article	Cancer preceding PD	Case-control	Denmark	National Hospital Register	1813	1887	\	Diagnosed; idiopathic PD	Yes	9
Dalvin	2017	Article	Both	1. Case-control 2. Matched cohort	Minnesota	Mayo clinic	974	2922	5	Coded	No	7
D'Amellio	2004	Article	Cancer preceding PD	Case-control	Italy		222	222	\	Diagnosed; idiopathic PD	Yes	8
Dinesh	2021	Article	Cancer preceding PD	Case-control	US	PPMI database	423	196	\	Diagnosed	No	8
Driver	2007	Article	PD preceding cancer	Matched cohort	US	Physicians' Health Study	487	487	5.2	Validated; idiopathic PD	Yes	8
Driver	2007	Article	Cancer preceding PD	Case-control	US	Physicians' Health Study	487	487	\	Validated; idiopathic PD	Yes	8
Driver	2008	Article	PD preceding cancer	Matched cohort	US	Physicians' Health Study	560	560	5.8	Validated; idiopathic PD	Yes	8
Elbaz	2002	Article	Cancer preceding PD	Case-control	Minnesota	Mayo clinic	196	196	5.5	Diagnosed	No	7
Fall	2003	Article	PD preceding cancer	Matched cohort	Sweden		170	510	4.8	Diagnosed	No	8
Elbaz	2005	Article	PD preceding cancer	Matched cohort	Minnesota	Mayo clinic	196	185	8	Diagnosed	Yes	6
Ferreira	2007	Article	Co-occurence	Cross-sectional	Portugal	The Lisbon University Hospital	150	146	\	Diagnosed; idiopathic PD	No	5
Fois	2010	Article	Both	Case-only cohort	UK	Oxford Record Linkage Study	4355		3.2	Coded	No	7

For peer review only - http://bmjopen.bmj.com/site/about/guidelines.xhtml

For peer review only

Author	Year	Publication type	Temporal direction	Study design	Location	Cohort	Case N	Control N	Follow time, y	Disease ascertainment	Smoking adjusted	Quality score
Freedman	2005	Article	Cancer preceding PD	Case-only cohort	US	SEER-Medicare	1900000		8.5	Coded	No	6
Freedman	2016	Letter	PD preceding cancer	Case-control	US (Asian Americans)	SEER-Medicare	20627	5558	\	Coded	No	7
Freedman	2016	Article	Cancer preceding PD	1. Case-control 2. cohort	US	SEER-Medicare	743779	419432	2.8	Coded	No	7
Gorell	1994	Article	Co-occurence	Cross-sectional	Michigan		8629	208933	\	Coded	No	7
Hely	1999	Article	PD preceding cancer	Case-only cohort	Australia	Sydney Multicenter Study of PD	130		9.1	Diagnosed	No	5
Jansson	1985	Article	Both	Prospective cohort	US		406		8.6	Diagnosed	Yes	\
Jamrozik	2005	Abstract	Cancer preceding PD	Case-control	Poland		100	100	\	\	No	7
Jespersen	2016	Article	Co-occurence	Case-control	Denmark	National Registry	45429	227145	\	Coded	No	7
Kareus	2012	Article	Cancer preceding PD	Case-control	US	Utah Cancer Registry	2300000			Coded	No	6
Kelm	2018	Abstract	Co-occurence	Case-control	US	Northwestern Medicine Enterprise Data Warehouse medical record	4751	9494	5.75	Coded	No	\
Lai	2013	Letter	Co-occurence	Case-control	Taiwan	National Health	2822	11288	\	Coded	Yes	8
Lai	2015	Article	Co-occurence	Case-control	Taiwan	National Health	1815	7260	\	Coded	No	8

For peer review only

For peer review only - http://bmjopen.bmj.com/site/about/guidelines.xhtml

Author	Year	Publication type	Temporal direction	Study design	Location	Cohort	Case N	Control N	Follow time, y	Disease ascertainment	Smoking adjusted	Quality score
Lerman	2018	Article	PD preceding cancer	Prospective cohort	Isreal	Maccabi Health Services	7727	1243968	\	Coded	Yes	9
Liao	2015	Article	PD preceding cancer	Case-control	Taiwan	National Health	13861	55444	\	Coded	No	8
Liao	2017	Article	PD preceding cancer	Case-control	Taiwan	National Health	64619	64619	\	Coded	No	8
Lin	2015	Article	PD preceding cancer	Matched cohort	Taiwan	National Health	62023	124046	\	Coded	No	6
Lo	2010	Article	Both	Matched cohort	US	PEAK	692	761	5.0; 4.3	Diagnosed	Yes	7
Minami	2000	Article	PD preceding cancer	Case-only cohort	Japan		228		6.97	Validated	No	6
Naghavi-Behzad	2016	Abstract	PD preceding cancer	Case-control	Iran		\		\	\	No	\
Olsen	2005	Denmark	PD preceding cancer	National Hospital Register			14088		5.0	Coded; idiopathic PD	No	8
Olsen	2006	Article	Cancer preceding PD	Case-control	Denmark	National Hospital Register	8090	32320	\	Coded; idiopathic PD	No	6
Olsen	2007	Article	PD preceding cancer	Case-only cohort	Denmark	National Hospital Register	14088		\	Coded; idiopathic PD	No	6
Ong	2014	Article	PD preceding cancer	Prospective cohort	UK	NHS hospital	219194	9015614	\	Coded	No	8
Ording	2019	Article	PD preceding cancer	Case-only cohort	Denmark	National Hospital Register	28835		4.0	Coded	No	7
Park	2019	Article	PD preceding cancer	Matched cohort	South Korea	NHI	52009	260045	\	Coded	No	8

For peer review only

For peer review only - http://bmjopen.bmj.com/site/about/guidelines.xhtml

Author	Year	Publication type	Temporal direction	Study design	Location	Cohort	Case N	Control N	Follow time, y	Disease ascertainment	Smoking adjusted	Quality score
Peretz	2016	Article	PD preceding cancer	Case-only cohort	Israel	Maccabi Health Services	7125		10.5	Validated	No	6
Pinter	2015	Article	PD preceding cancer	Case-only cohort	Austria		237		14.8	Coded	No	6
Piri	2016	Abstract	PD preceding cancer	Prospective cohort		Cancer Registry Database	2584		\	Diagnosed	No	\
Powers	2006	Article	Co-occurence	Case-control	Seattle		352	484	\	Diagnosed; idiopathic PD	Yes	8
Pressley	2003	Article	Co-occurence	Cross-sectional	US	National Long-Term Care Survey	791	24040	\	Coded	No	6
Rugbjerg	2012	Article	PD preceding cancer	Case-only cohort	Denmark	National Hospital Register	20343		5.7	Coded	No	6
Ryu	2020	Article	PD preceding cancer	Matched cohort	Korea	South Korea National Health Insurance System	70780	353900	8	Diagnosed	No	7
Schwid	2010	Article	PD preceding cancer	Case-only cohort	US	PRECEPT	806		1.8	Diagnosed/verified	No	4
Shalaby	2016	Article	Co-occurence	Case-control	US	Columbia University Medical Cente	108	124	\	Self-report	No	6
Sun	2011	Article	PD preceding cancer	Matched cohort	Taiwan	NHI	4957	19828	\	Coded	No	8
Tacik	2016	Article	1. Co-occurrence 2. cancer preceding PD	Prospective cohort	Florida	Mayo clinic	971	478	4.6	Diagnosed	No	6

For peer review only - http://bmjopen.bmj.com/site/about/guidelines.xhtml

Author	Year	Publication type	Temporal direction	Study design	Location	Cohort	Case N	Control N	Follow time, y	Disease ascertainment	Smoking adjusted	Quality score
Tang	2016	Article	PD preceding cancer	Matched cohort	Taiwan	NHI	2998	11992	\	Coded	No	7
Vanacore	1999	Communication	PD preceding cancer	Case-only cohort	Italy		10322		5.7	Drug	No	4
Wing	2012	Abstract	Both	Prospective cohort	UK		8549	42160	\	\	Yes	\
Winter	2016	Article	PD preceding cancer	Matched cohort	US	Women's Health Study	396	396	6.2	Self-report	Yes	7
Wirdefeldt	2014	Article	Both	Matched cohort	Sweden		11786	58930	\	Coded	No	6

Study design and temporal direction was defined per each individual study definition, most of which was based on the diagnosis date of two diseases. Disease ascertainment was defined per the description of whether any physicians, neurologists or movement specialists made the diagnosis. Quality score was assessed by the Newcastle-Ottawa Scale for cohort studies and for case-control studies (range 0–9).

For peer review only - http://bmjopen.bmj.com/site/about/guidelines.xhtml

Table 2. Subgroup-analysis of the association between Parkinson disease and cancer.

	No. of publications	Pooled RR (95% CI)	P for significance	P for heterogeneity	P difference
**Age**					**0.10**
< 69.3 years	13	0.70 (0.42, 1.19)	0.21	<0.001	
≥ 69.3 years	14	0.90 (0.81, 1.00)	0.05	<0.001	
**Sex**					**0.31**
Men-dominant	23	0.76 (0.57, 1.02)	0.07	<0.001	
Women-dominant	12	0.91 (0.70, 1.17)	0.45	<0.001	
**Ethnicity**					**0.19**
Caucasian-dominant	27	0.75 (0.59, 0.96)	0.02	<0.001	
Asian-dominant	6	0.98 (0.75, 1.28)	0.88	<0.001	
**Study design**					**0.92**
Prospective cohort	24	0.79 (0.65, 0.96)	0.05	<0.001	
Other	9	0.79 (0.65, 0.96)	0.02	<0.001	
**Newcastle-Ottawa quality score**					**0.31**
≤ 6	12	0.87 (0.71, 1.08)	0.21	<0.001	
≥ 7	21	0.75 (0.57, 0.98)	0.04	<0.001	
**Period of study**					**0.19**
< 2010	16	0.73 (0.61, 0.88)	0.001	<0.001	
≥ 2010	17	0.88 (0.80, 0.96)	0.003	<0.001	

6 publications did not report mean/median age or age range.

2 publications did not report sex ratio. 4 publications separately report risk estimates for men and women, therefore counted in both sex groups.

For peer review only

For peer review only - http://bmjopen.bmj.com/site/about/guidelines.xhtml

Table 3. Publications on risk of total cancer associated with levodopa treatment.

Publication	Estimation (95% confidence interval)	Note
Elbaz, 2005	1.26 (0.39, 4.12)	4th (>1,313 g) compared to 1st quartile of cumulative levodopa
Constanescu, 2007	1.4 (0.3, 4.3)	After levodopa use
Olsen, 2007	1.0 (0.5, 2.0)	≥1370 g compared to 600-1369 g of cumulative levodopa
Becker, 2010	0.7 (0.56, 0.88)	≥5 prescription of levodopa

For peer review only

For peer review only - http://bmjopen.bmj.com/site/about/guidelines.xhtml

Supplementary figures

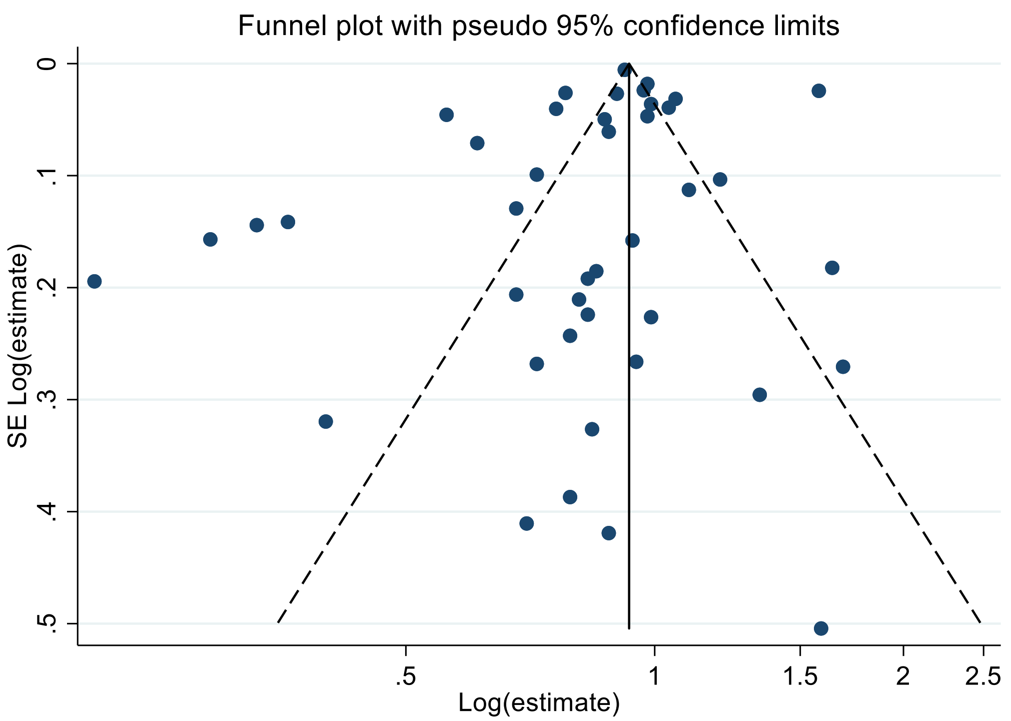

Figure 1. Funnel plot of studies of the association between Parkinson Disease and total cancer. The log-transformed risk estimates from each study is plotted on the horizontal axis, and its standard error is plotted on the vertical axis.

For peer review only - http://bmjopen.bmj.com/site/about/guidelines.xhtml

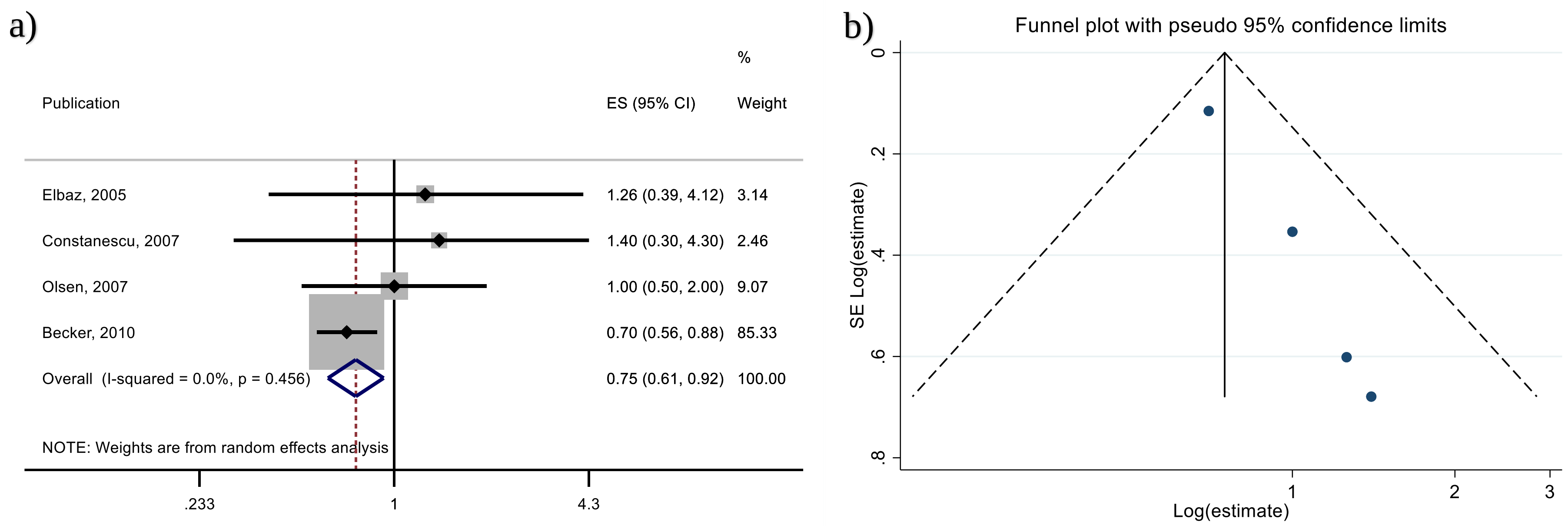

Figure 2. a) Individual and pooled estimates of the association between use of levodopa and risk of total cancer in 4 publications. Figure shows the estimates (ESs) and 95% confidence intervals (CIs) for each individual studies and the pooled result from random effects model. b) Funnel plots of these 4 publications.

For peer review only - http://bmjopen.bmj.com/site/about/guidelines.xhtml

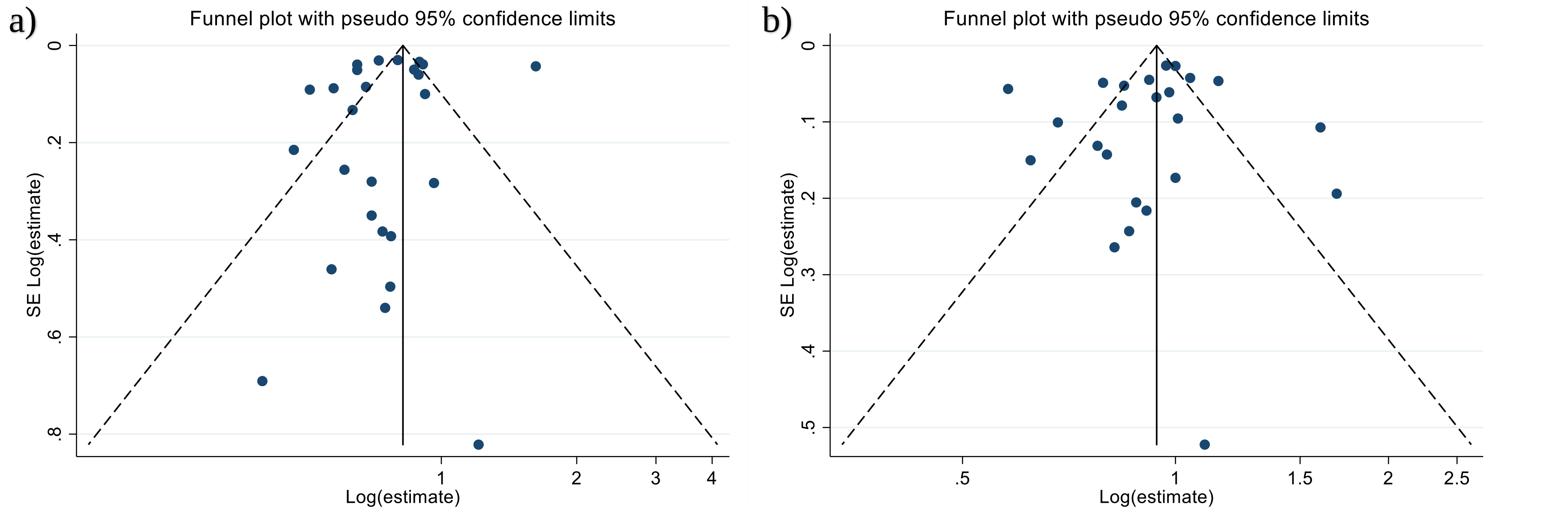

Figure 3. Funnel plot of studies of the association between Parkinson Disease and a) smoking-related cancers, b) non-smoking-related cancers. The log-transformed risk estimates from each study is plotted on the horizontal axis, and its standard error is plotted on the vertical axis.

For peer review only - http://bmjopen.bmj.com/site/about/guidelines.xhtml

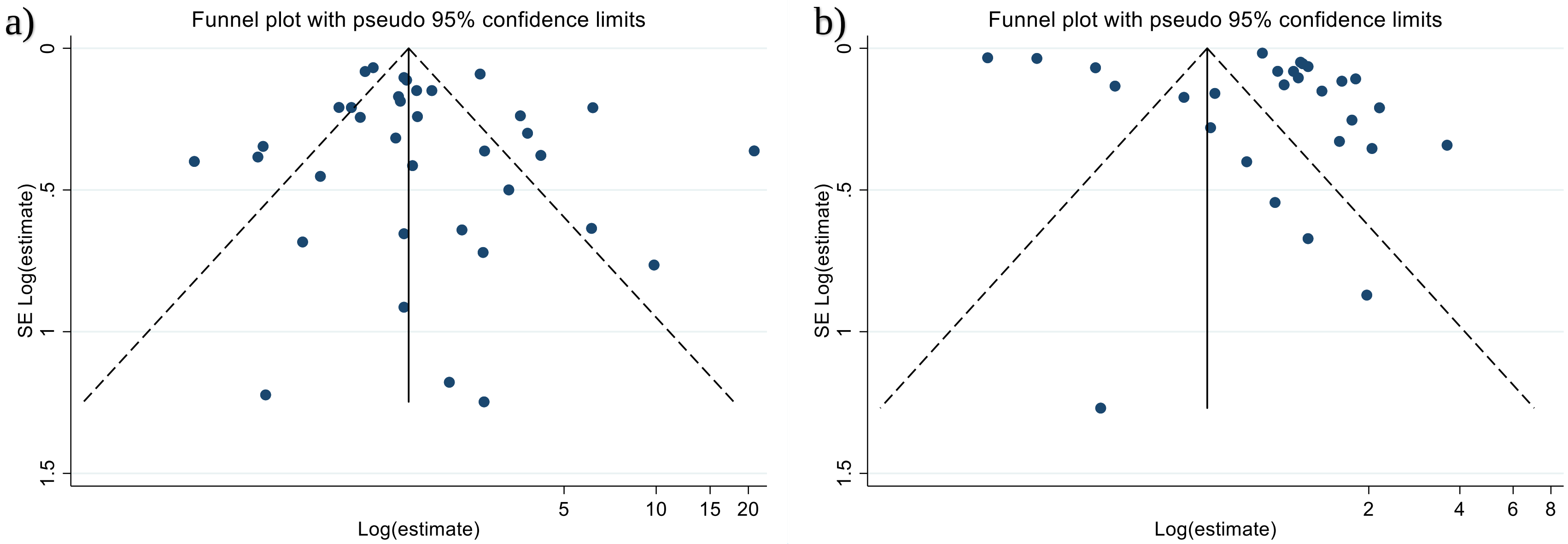

Figure 4. Funnel plot of studies of the association between Parkinson Disease and a) melanoma, b) non-melanoma skin cancers. The log-transformed risk estimates from each study is plotted on the horizontal axis, and its standard error is plotted on the vertical axis.

For peer review only - http://bmjopen.bmj.com/site/about/guidelines.xhtml

PRISMA 2009 Checklist

Section/topic	#	Checklist item	Reported on page #
**TITLE**			
Title	1	Identify the report as a systematic review, meta-analysis, or both.	1
**ABSTRACT**			
Structured summary	2	Provide a structured summary including, as applicable: background; objectives; data sources; study eligibility criteria, participants, and interventions; study appraisal and synthesis methods; results; limitations; conclusions and implications of key findings; systematic review registration number.	2 & 3
**INTRODUCTION**			
Rationale	3	Describe the rationale for the review in the context of what is already known.	5
Objectives	4	Provide an explicit statement of questions being addressed with reference to participants, interventions, comparisons, outcomes, and study design (PICOS).	5
**METHODS**			
Protocol and registration	5	Indicate if a review protocol exists, if and where it can be accessed (e.g., Web address), and, if available, provide registration information including registration number.	7 and supplementary material
Eligibility criteria	6	Specify study characteristics (e.g., PICOS, length of follow-up) and report characteristics (e.g., years considered, language, publication status) used as criteria for eligibility, giving rationale.	7
Information sources	7	Describe all information sources (e.g., databases with dates of coverage, contact with study authors to identify additional studies) in the search and date last searched.	7
Search	8	Present full electronic search strategy for at least one database, including any limits used, such that it could be repeated.	7
Study selection	9	State the process for selecting studies (i.e., screening, eligibility, included in systematic review, and, if applicable, included in the meta-analysis).	7
Data collection process	10	Describe method of data extraction from reports (e.g., piloted forms, independently, in duplicate) and any processes for obtaining and confirming data from investigators.	8
Data items	11	List and define all variables for which data were sought (e.g., PICOS, funding sources) and any assumptions and simplifications made.	8
Risk of bias in individual studies	12	Describe methods used for assessing risk of bias of individual studies (including specification of whether this was done at the study or outcome level), and how this information is to be used in any data synthesis.	8
Summary measures	13	State the principal summary measures (e.g., risk ratio, difference in means).	8

For peer review only

PRISMA 2009 Checklist

Synthesis of results	14	Describe the methods of handling data and combining results of studies, if done, including measures of consistency (e.g., I^2) for each meta-analysis.	9

Section/topic	#	Checklist item	Reported on page #
Risk of bias across studies	15	Specify any assessment of risk of bias that may affect the cumulative evidence (e.g., publication bias, selective reporting within studies).	8
Additional analyses	16	Describe methods of additional analyses (e.g., sensitivity or subgroup analyses, meta-regression), if done, indicating which were pre-specified.	9
**RESULTS**			
Study selection	17	Give numbers of studies screened, assessed for eligibility, and included in the review, with reasons for exclusions at each stage, ideally with a flow diagram.	10 and supplementary material
Study characteristics	18	For each study, present characteristics for which data were extracted (e.g., study size, PICOS, follow-up period) and provide the citations.	10, Table 1, and Supplementary material
Risk of bias within studies	19	Present data on risk of bias of each study and, if available, any outcome level assessment (see item 12).	10, 11, and supplementary material
Results of individual studies	20	For all outcomes considered (benefits or harms), present, for each study: (a) simple summary data for each intervention group (b) effect estimates and confidence intervals, ideally with a forest plot.	10, 11, Figure 1-4
Synthesis of results	21	Present results of each meta-analysis done, including confidence intervals and measures of consistency.	10, 11
Risk of bias across studies	22	Present results of any assessment of risk of bias across studies (see Item 15).	10, 11, and supplementary material
Additional analysis	23	Give results of additional analyses, if done (e.g., sensitivity or subgroup analyses, meta-regression [see Item 16]).	10
**DISCUSSION**			
Summary of evidence	24	Summarize the main findings including the strength of evidence for each main outcome; consider their relevance to key groups (e.g., healthcare providers, users, and policy makers).	12-15

For peer review only

PRISMA 2009 Checklist

Limitations	25	Discuss limitations at study and outcome level (e.g., risk of bias), and at review-level (e.g., incomplete retrieval of identified research, reporting bias).	12,14,15
Conclusions	26	Provide a general interpretation of the results in the context of other evidence, and implications for future research.	15
**FUNDING**			
Funding	27	Describe sources of funding for the systematic review and other support (e.g., supply of data); role of funders for the systematic review.	16

From: Moher D, Liberati A, Tetzlaff J, Altman DG, The PRISMA Group (2009). Preferred Reporting Items for Systematic Reviews and Meta-Analyses: The PRISMA Statement. PLoS Med 6(7): e1000097. doi:10.1371/journal.pmed1000097

For more information, visit: **www.prisma-statement.org**.

For peer review only

BMJ Open

Parkinson’s disease and cancer: a systematic review and meta-analysis of over 17 million participants

Journal:	*BMJ Open*
Manuscript ID	bmjopen-2020-046329.R2
Article Type:	Original research
Date Submitted by the Author:	24-May-2021
Complete List of Authors:	Zhang, Xinyaun; The Pennsylvania State University, Nutritional Science<br>Guarin, David; Massachusetts General Hospital, Neurology<br>Mohammadzadehhonarvar, Niyaz; Massachusetts General Hospital, Neurology<br>Chen, Xiqun; Massachusetts General Hospital, Neurology<br>Gao, Xiang; The Pennsylvania State University, Nutritional Science
**Primary Subject Heading**:	Epidemiology
Secondary Subject Heading:	Neurology
Keywords:	ONCOLOGY, EPIDEMIOLOGY, Parkinson-s disease < NEUROLOGY, Epidemiology < ONCOLOGY

SCHOLARONE™
Manuscripts

I, the Submitting Author has the right to grant and does grant on behalf of all authors of the Work (as defined in the below author licence), an exclusive licence and/or a non-exclusive licence for contributions from authors who are: i) UK Crown employees; ii) where BMJ has agreed a CC-BY licence shall apply, and/or iii) in accordance with the terms applicable for US Federal Government officers or employees acting as part of their official duties; on a worldwide, perpetual, irrevocable, royalty-free basis to BMJ Publishing Group Ltd ("BMJ") its licensees and where the relevant Journal is co-owned by BMJ to the co-owners of the Journal, to publish the Work in this journal and any other BMJ products and to exploit all rights, as set out in our licence.

The Submitting Author accepts and understands that any supply made under these terms is made by BMJ to the Submitting Author unless you are acting as an employee on behalf of your employer or a postgraduate student of an affiliated institution which is paying any applicable article publishing charge ("APC") for Open Access articles. Where the Submitting Author wishes to make the Work available on an Open Access basis (and intends to pay the relevant APC), the terms of reuse of such Open Access shall be governed by a Creative Commons licence – details of these licences and which Creative Commons licence will apply to this Work are set out in our licence referred to above.

Other than as permitted in any relevant BMJ Author's Self Archiving Policies, I confirm this Work has not been accepted for publication elsewhere, is not being considered for publication elsewhere and does not duplicate material already published. I confirm all authors consent to publication of this Work and authorise the granting of this licence.

For peer review only

Parkinson's disease and cancer: a systematic review and meta-analysis of over 17 million participants

Xinyuan Zhang, BS[1#], David Guarin, BA[2#], Niyaz Mohammadzadeh honarvar,PhD[2], Xiqun Chen, MD, PhD[1*], Xiang Gao, MD, PhD[2*]

[1]Department of Nutritional Sciences, The Pennsylvania State University, University Park, PA 16802, USA

[2]Department of Neurology, Massachusetts General Hospital, Harvard Medical School, Boston, MA 02114, USA

#Co-first author; *Co-senior author

Correspondence should be addressed to Xiqun Chen**;** xchen17@mgh.harvard.edu

Word Count: 2690

Keywords: Neoplasms; Epidemiology; Meta-analysis; Parkinson's disease; Odds ratio.

For peer review only

Abstract

Objective

To systematically review and qualitatively evaluate epidemiological evidence on associations between PD and cancer via meta-analysis.

Data Sources

MEDLINE via PubMed, Web of Science, and EMBASE, until March 2021.

Study Selection

Included were publications that 1) were original epidemiological studies on PD and cancer; 2) reported risk estimates; 3) were in English. Exclusion criteria included: 1) review/comments; 2) biological studies; 3) case report/autopsy studies; 4) irrelevant exposure/outcome; 5) treated cases; 6) no measure of risk estimates; 7) no confidence intervals/exact p values; and 8) duplicates.

Data extraction and Synthesis

PRISMA and MOOSE guidelines were followed in data extraction. Two-step screening was performed by two authors blinded to each other. A random-effects model was used to calculate pooled relative risk (RR).

Main Outcomes and Measures

We included publications that assessed the risk of PD in individuals with vs without cancer and the risk of cancer in individuals with vs without PD.

Results

For peer review only - http://bmjopen.bmj.com/site/about/guidelines.xhtml

A total of 63 studies and 17,994,584 participants were included. Meta-analysis generated a pooled relative risk of 0.82 (n = 33; 95% CI: 0.76, 0.88; p <0.001) for association between PD and total cancer, 0.76 (n = 21; 95% CI: 0.67, 0.85; p <0.001) for PD and smoking-related cancer, and 0.92 (n = 19; 95% CI: 0.84, 0.99; p = 0.03) for non-smoking-related cancer. PD was associated with an increased risk of melanoma (n = 29; pooled relative risk = 1.75; 95% CI: 1.43, 2.14; p <0.001) but not for other skin cancers (n=17; pooled relative risk = 0.90; 95%CI: 0.60, 1.34; p = 0.60).

Conclusions

PD and total cancer were inversely associated. This inverse association persisted for both smoking-related and non-smoking-related cancers. PD was positively associated with melanoma. These results provide evidence for further investigations for possible mechanistic associations between PD and cancer.

For peer review only

For peer review only - http://bmjopen.bmj.com/site/about/guidelines.xhtml

Article Summary

Strengths and limitations of this study

- Unlike recent meta-analyses, this study stratifies analysis for smoking vs non-smoking cancers.
- Heterogeneity between included studies was analyzed via meta-regression.
- Despite best efforts, high heterogeneity in methodology and cohorts of included studies cannot be fully dealt with by statistical methods.

For peer review only

For peer review only - http://bmjopen.bmj.com/site/about/guidelines.xhtml

Introduction

Parkinson's disease (PD) is the second most common neurodegenerative disease affecting more than 10 milion people worldwide. It is characterized by premature cell death of dopaminergic neurons in the substantia nigra pars compacta (SNpc) . Clinically, PD is manifested by tremor, rigidity, bradykinesia and postural instability. Non-motor symptoms are also common. Symptomatic treatments for PD are available and effective, however there is currently no therapy known to modify disease progression. Among enviromental factors that have been associated with the risk of developing PD, age is the main risk factor, whereas smoking has been inversely associated with PD[1 2]. Familial PD accounts for 5%–15% of total PD. The most common genetic cause of PD is mutations in *LRRK2*. Other PD-related genes include *PARK2*, *PARK7*, *PINK1*, and *SNCA*. PD is increasingly recognized as a systemic disorder. Oxidative stress, mitochondria dysfuntion, energy failure, immune dysregulation and chronic inflammation have been proposed to contribute to neurodegeneration in PD [3].

Cancer is characterized by uncontrolled cell proliferation and growth. It is among the leading causes of death worldwide [4]. Growing evidence suggests that PD and cancer may be associated [5]. Similar to PD, cancer incidence increases with age [6]. Smoking also modifies the risk of certain cancer, especially lung cancer, though in the opposite direction to the risk of PD [7]. In addition, PD related genes have been implicated in cancer. *PARK2* has been identified as a potent tumor suppressor gene, whereas mutations in *LRRK2* have been associated with an increased risk of cancer [8]. While a positive, bidirectional link between PD and melanoma, a malignant tumor that develops from melanocytes is well-documented [9], there appears to be an inverse association between PD and total cancer [10]. However, it remains unclear whether PD and cancer are associated mechanistically, or the findings were confounded by other factors, such as study

For peer review only - http://bmjopen.bmj.com/site/about/guidelines.xhtml

designs and smoking. Clearly documenting these associations is important for bridging the interdisciplinary knowledge gap and developing novel preventive and treatment strategies for both PD and cancer. An individual study may lack the power to detect an association. A meta-analysis can increase precision in estimating risk [11], especially in subsets of cancers with even fewer cases. We thus conducted a meta-analysis to systematically review the population-based evidence for the potential association between PD and cancer. To better elucidate PD-cancer relation, we first stratified studies according to the temporal association between the two diseases into three categories: PD preceding cancer, cancer preceding PD, and co-occurrence. Secondly, we performed sensitivity analyses in which variations in study design and qualities, and levodopa treatment, were evaluated. Thirdly, we separately analyzed smoking-related cancers and non-smoking-related cancers to address smoking as a potential confounding factor. Finally, we specifically analyzed the associations between PD and melanoma, non-melanoma skin cancers, and other major cancers (eg, prostate cancer, colon cancer, and breast cancer).

Methods

Literature search and data extraction

This meta-analysis followed the MOOSE guidelines for reporting meta-analysis on observational studies and was registered on PROSPERO (CRD42020162103). We searched all published literature that reported PD association with cancer in MEDLINE via PubMed, Web of Science, and EMBASE up to March 1, 2021. Search items related to "Parkinson's disease", "cancer", and "epidemiologic studies" were identified and modified for each database. We constrained our search in human studies and in the English language. Detailed search terms can be found in

For peer review only - http://bmjopen.bmj.com/site/about/guidelines.xhtml

Supplementary materials. Duplicates were matched based on author, year, and title in Endnote X9 and manually compared before removing.

The inclusion criteria were: 1) original studies that were conducted in an epidemiological setting; 2) studies reported either an odds ratio (OR), risk ratio (RR), hazard ratio (HR), standardized incidence/mortality ratio (SIR/SMR), or other reliable measures of estimated risk; 3) studies in which PD and cancer cases were ascertained by doctor's diagnosis, hospitalization record, disease identification codes, or self-report on the diagnosis. Exclusion criteria included: 1) reviews or comments; 2) non-epidemiological studies; 3) case reports/autopsy studies; 4) irrelevant exposure/outcome; 5) treated cases; 6) no measure of risk estimates; 7) no confidence intervals/exact p values; and 8) duplicates. Parkinsonism that does not meet the criteria for PD and benign neoplasm were not included. Previous meta-analyses were used as references for manual searching of related publications. Two first authors (X. Z., BS and D. G., BA) independently screened the publications in two steps: title/abstract screening and full-text screening. Any discrepancy was reviewed and reconciled by two senior authors (X. C. and X. G.). During full-text screening, we found 5 groups of publications using the same population or dataset. Details of inclusion and exclusion step are reported in Supplementary methods. After screening references of included publications, we found two other eligible publications that were not captured by search items [12 13].

Data extraction

From each of the included publications, we extracted information on the first author, year of the study, study type, country origin, population, mean age, dominant sex, dominant ethnicity, cases and controls population size, measure of risk, PD and cancer ascertainment methods, adjusted

For peer review only - http://bmjopen.bmj.com/site/about/guidelines.xhtml

covariates, levodopa use, and estimated risk with lower and upper confidence intervals (CIs) for each type of cancer. The temporal association was defined per each individual study definition, most of which was based on the diagnosis date of the two diseases. Dominant sex and ethnicity were defined as the major sex and race/ethnicity (>50%) of the studied population, respectively.

The type of study was categorized into prospective study, case-control study, case-only cohort study, and cross-sectional study.

Statistical analysis

All analyses were performed in STATA SE 15. Cochran's Q statistic and I-squared were calculated to examine heterogeneity among studies. Cochran's Q was computed as the sum of variance from the pooled estimates and compared to chi-squared distribution with *k*-1 (*k* = number of publications) degree of freedom. I-squared was calculated as the percentage of variation across studies due to heterogeneity rather than chance [14]. Due to the high heterogeneity of included publications (p-value for Q statistics <0.05, I-squared >50% for all), pooled effect sizes (including RR, OR, HR, SIR, and SMR) were calculated using random-effects models to account for unobserved heterogeneity. Egger test and funnel plots were performed to assess publication bias.

For total cancer, we performed three sensitivity analyses. First, 4 publications from meeting proceedings/abstracts were further included; second, 8 mortality publications were excluded; third, 2 publications using invalidated, self-report diagnosis of either cancer or PD were excluded. Further, we performed six subgroup analyses, looking at the variance of the included publications in population age, dominant sex, dominant race/ethnicity, study design, study quality, and year of study. Age was separated into two groups by the mean age of the included

For peer review only - http://bmjopen.bmj.com/site/about/guidelines.xhtml

studies (69.3 years). Dominant ethnicity was categorized into Caucasian-dominant and Asian-dominant. The study design was categorized into cohort studies and other types of studies. Study quality was assessed by the Newcastle-Ottawa Scale for cohort studies and for case-control studies [15], which is based on the definition of case/control, the definition of exposure/outcome, covariates, and other relevant factors. The score ranged from 0–9, and we separated the included studies into low quality group (< 7) and high quality group (≥7), based on the mean quality score of the included studies. Proceedings/abstracts were not included in the quality check. The difference between groups was tested by the meta-regression method.

We categorized cancers into smoking-related and non-smoking-related cancers according to National Cancer Institute and Centers for Disease Control and Prevention's definition [16]. Smoking-related cancers include cancer of the lung, larynx, mouth, esophagus, throat, bladder, kidney, liver, stomach, pancreas, colon and rectum, and cervix, as well as acute myeloid leukemia. Cancers of other sites, including melanoma, were regarded as not associated with smoking. If a publication reported grouped smoking- and non-smoking-related cancers, the risk estimates were extracted directly. If a publication reported individual cancers only, and the number of sites is more than 10, we first categorized individual cancers into smoking-related and non-smoking-related groups accordingly [16], calculated pooled RR and 95% CI in each group using a random-effects model, and then included the resulting pooled RR in the final meta-analysis.

We specifically evaluated the association between PD and melanoma, and other skin cancers. Cancers of other specific sites were included in this meta-analysis if there were more than 10 publications. Included were lung cancer, colorectal cancer, breast cancer, and prostate cancer.

For peer review only - http://bmjopen.bmj.com/site/about/guidelines.xhtml

Results

In total, we included 63 publications in this meta-analysis (Figure 1) [12 13 17-77]. Characteristics of all publications are listed in Supplementary table 1.

PD and total cancer

Combining 33 publications [12 13 18 27-32 35-39 41 50-52 54 57-62 64 65 69-71 73 75 76], pooled RR for association between PD and cancer was 0.82 (95% CI: 0.76, 0.88; p <0.001; Figure 2). We did not observe evidence for existence of publication bias (Egger test p = 0.27; supplementary figure 1). After stratified by temporal sequence, PD was significantly associated with a lower future risk of cancer (n = 21, pooled RR = 0.85; 95% CI: 0.76, 0.95; p = 0.004), and similar association was observed for cancer with a lower future risk of PD (n = 11, pooled RR = 0.74; 95% CI: 0.65, 0.85; p = <0.001). The significant inverse association persisted after further including meeting abstracts, excluding mortality studies, and excluding self-report outcomes that were not validated (table 1). Meta regression did not find significant difference between subgroups stratified by age (<69.3 years vs ≥69.3 years; mean value of the included studies), sex (men- vs women-dominant cohorts), ethnicity (Caucasian vs Asian), study design (cohort vs others), study quality (scored <7 vs ≥ 7), or year of study (before 2010, or 2010 and after, supplementary table 2).

We found 4 publications that examined the risk of cancers associated with the treatment of levodopa in PD patients (supplementary table 3) [18 24 31 56]. Although there was a significant lower risk of cancer after levodopa treatment or with higher cumulative levodopa treatment (pooled RR = 0.75; 95% CI: 0.61, 0.92; p = 0.007; supplementary figure 2a), Egger test (p =

For peer review only - http://bmjopen.bmj.com/site/about/guidelines.xhtml

0.005) and funnel plot (supplementary figure 2b) showed a significant publication bias and thus a potentially over-estimated result.

Smoking- and non-smoking-related cancers

Combining 21 publications [12 18 28 30-32 35 37 38 50 51 54 57-60 66 70 71 73 76], the pooled RR for association between PD and smoking-related cancers was 0.76 (95% CI: 0.67, 0.85; p <0.001; figure 3a). PD was also inversely associated with non-smoking-related cancers (n = 19; pooled RR = 0.92; 95% CI: 0.84, 0.99; p = 0.03; figure 3b) [12 18 25 28 30 31 35 37 38 50 51 57-59 66 70 71 73 76]. No publication bias was observed for both analyses (Egger test p = 0.45 and 0.50, respectively; supplementary figure 3).

Melanoma and non-melanoma skin cancer

Combining 29 publications [17 18 20 23 24 26 30 32 35 37 38 43 47 50 51 54 56 57 59 60 64 68-71 73 76-78], the pooled RR for association between PD and melanoma was 1.75 (95% CI: 1.43, 2.14; p <0.001; figure 4a). No publication bias was observed (Egger test p = 0.28; supplementary figure 4a). We did not find a statistically significant association between PD and non-melanoma skin cancer (n = 17; pooled RR = 0.90; 95% CI: 0.60, 1.34; p = 0.60; figure 4b) [31 32 34 35 37 41 47 50 54 56 57 67 69 71 73 76 77]. Egger test suggested no publication bias (p = 0.53), but funnel plot suggested potential over-estimation by small studies (supplementary figure 4b).

Other site-specific cancers

Lung cancer and colorectal cancer, two major cancers in the smoking-related category, both showed a significant inverse association with PD. There was no significant association between PD and breast cancer and prostate cancer (Table 1).

For peer review only - http://bmjopen.bmj.com/site/about/guidelines.xhtml

Discussion

In this meta-analysis of 63 publications and 17,994,584 participants, a significant inverse association between PD and total cancer was observed, with an 18% lower risk on both sides. Individuals with PD had a 15% lower risk of developing cancer, and vice versa, individuals with cancer had a 26% lower risk of developing PD. The inverse association was stronger for smoking-related cancers, compared to non-smoking-related cancers, though both achieved statistical significance. In contrast, PD was significantly associated with a 75% higher risk of melanoma. The overall inverse association is consistent with two published meta-analyses on this topic, which reported a 27% and 6% significantly lower risk for total cancer, respectively [10 79]. Relative to these two published meta-analyses, our study included a large number of studies and participants. The latest meta-analysis, for example, included 15 studies and 1,480,239 participants for examining the association between PD and total cancer [10 79] . In addition, this study did not stratify smoking-related and non-smoking-related cancers despite the analysis of associations between PD and specific cancers. Further, these two meta-analyses included both PD and parkinsonism [10 79].

One of the possible explanations for the inverse association between PD and total cancer is smoking. Smoking has been consistently associated with a low risk of PD and a high risk of many types of cancer [7]. Moreover, there is evidence that PD patients are less likely to be smokers [80]. Of note, only 10 out of the 32 publications included were adjusted for smoking behavior for total cancer risk in their original analysis [18 25 27-31 51 64 75], which may introduce residual confounding for the observed association between PD and total cancer. However, we also found that non-smoking-related cancer was inversely associated with PD, even when melanoma was included. Because only 4 publications separately reported risk estimates for total cancer or non-

For peer review only - http://bmjopen.bmj.com/site/about/guidelines.xhtml

smoking-related cancers after excluding melanoma[25 54 55 76], we did not perform a meta-analysis in these secondary categories. Our findings suggest that smoking is unlikely the only factor contributing to the observed inverse relation between PD and total cancer. Future studies with carefully adjusted smoking habits or environmental smoking exposure are warranted to better address this issue.

Our results, in line with the previous meta-analysis [10 79], suggest an inverse comorbidity between PD and cancer. The biological bases underlying the association is far from clear. Dysregulated cellular processes including those involved in the regulation of cell cycle, mitochondrial function, DNA repair, cell metabolism, and immune responses have been implicated in degeneration of neurons and tumorigenesis in dividing cells, often in the opposite directions. Cell proliferation and survival signals such as Wnt, P53, and PI3K/AKT may be upregulated in cancer and downregulated in neurodegeneration. The ubiquitin proteasome pathway of protein degradation on the other hand may be downregulated in neurodegeneration and upregulated in cancer [81-83]. Understanding the biological pathways would further facilitate investigations on potential strategies for better prevention, surveillance, and treatment of both PD and cancer.

Several common gene mutations have been implicated in PD and cancer [84]. *PARK2* was found to be a potent tumor suppressor gene [85 86] . Other PD-related genes *PINK1*, *PARK7*, and *LRRK2* have also been linked to cancer [60 87 88] . PD patients carrying *LRRK2* G2019S mutation have been associated with an overall increased risk of cancer, especially for hormone-related cancer and breast cancer [85], and most recently, lukemia, colon cancer, and skin cancer when compared with noncarrier PD [89]. Another PD-related *LRRK2* mutation R1441G was found to be associated with higher prevalence of hematological cancers [90]. Both G2019S and R1441G show increased LRRK2 kinase activity [91]. However, a recent study demonstrated that loss of *LRRK2* could

For peer review only - http://bmjopen.bmj.com/site/about/guidelines.xhtml

promote lung cancer development, adding to the complexicity of LRRK2-cancer link [92]. We found that only 14 of the included studies specifically identified idiopathic PD and excluded genetically determined PD. This limits our systematic review to distinguish genetic forms of PD from idiopathic PD and fully synthesize the potential genetic overlaps between PD and cancer.

Although similarly characterized pathologically by over proliferation, different cancers are highly heterogeneous. While it remains to be determined whether the general inverse association exists across cancers of different sites and evolutionary origins, we and others have consistently shown that it did not apply to melanoma [9 93]. In this meta-analysis, we replicated the well-documented positive link between PD and melanoma. It has long been proposed that levodopa as the mainstay therapy for PD and a common precursor for both dopamine and melanin may contribute to the higher risk of melanoma in PD [94 95]. In this meta-analysis, we found a 37% higher risk of newly-developed PD after diagnosis of melanoma, suggesting that the observed PD-melanoma association may not be fully explained by the role of levodopa, if any [96]. Previously, we reported that the risk of incident PD is higher in people with a family history of melanoma among their first-degree relatives [93]. One plausible biological explanation of the association is the regulation of pigmentation by the *MC1R* gene, which presents and functions in both melanocytes and dopaminergic neurons [97 98] . Other genetic mutations, such as *CYP2D6* polymorphism and *VDR* polymorphism, might also be involved in both conditions [99-102] .

Despite all our effort in synthesizing all epidemiological evidence, the intrinsic limitations of meta-analysis cannot be avoided. First, studies included in this analysis came from diverse populations, with diverse designs and treatment strategies. They varied across assessments, statistical methods, and adjusted covariates. Although meta-regression did not find differences in age, sex, ethnicity, study design, and study quality, the highly heterogeneous nature of this meta-

For peer review only - http://bmjopen.bmj.com/site/about/guidelines.xhtml

analysis limits its interpretation into robust conclusions. Second, due to lack of access to original data, we could not adjust uniformly for confounders. We addressed this shortcoming by stratifying cancers into smoking-related or non-smoking-related cancers. However, there may be residual confounding since only a few studies adjusted for family history of PD/cancer, use of medications, sun exposure, duration of PD/cancer, use of medical care, or diet (eg, caffeine consumption) [103-105]. Third, many large-scale studies included in this meta-analysis used local/national registry databases, with disease diagnosis mostly based on International Classification of Disease codes. Notification to registries might not be complete, therefore the cases might be under-reported. Moreover, diagnosis criteria may slightly vary in different countries, hospitals, etc. Thus it is challenging to confirm and validate the information from these datasets. Lastly, all publications included in this meta-analysis were based on populations from North America, Europe, Australia, and Central and East Asia; No study has examined the association of PD and cancer in less-developed regions such as Africa, Southeast Asia, or South America. This could be due to difficulties in disease diagnosis and registry in these regions. Recent findings suggested positive associations between PD and most cancers in an East Asian population, highlighting possible discrepancies among different populations with different ethnic backgrounds [50 88]. Future studies should address the potentially important role of race/ethnicity and social-economic status.

We reviewed the current epidemiological evidence for the association between cancer and PD, with a meta-analysis of over 17 million individuals. We found that PD was associated with low risk of total cancer, except for melanoma, with which a positive association was identified. Despite the limitations, our study provided an overall picture of the association between the two

For peer review only - http://bmjopen.bmj.com/site/about/guidelines.xhtml

major disease entities. Future studies should aim to better understand the links between these two major chronic disease entities using epidemiological, clinical, and biological approaches.

Funding: This work was supported by the National Institutes of Health [R01NS102735], the Parkinson's Disease Foundation [PF-APDA-SFW-1914], the Jane & Alan Batkin Foundation [N/A], the Farmer Family Foundation [N/A], and the Michael J. Fox Foundation and the Aligning Science Across Parkinson's Initiative (ASAP-000312). None of the listed funders had a role in the design and conduct of the study; collection, management, analysis, and interpretation of the data; preparation, review, or approval of the manuscript; and decision to submit the manuscript for publication.

Data Availability: The data used to support the findings of this article are included within the article and the supplementary material.

Patient and Public Involvement Statement: It was not appropriate to involve patients or the public in the design, or conduct, or reporting, or dissemination plans of our research.

Ethics Statement: N/A

Competing Interests: The authors declare no conflicts of interest.

Acknowledgements: We thank Christina Wissinger, PhD, from Health Sciences Liaison Librarian of Penn State, who helped with search strategy. No financial compensation was made.

Author Contributions

Name	Location	Contribution

For peer review only - http://bmjopen.bmj.com/site/about/guidelines.xhtml

Xinyuan Zhang, BSc	The Pennsylvania State University	Concept and design; Acquisition, analysis, or interpretation of data; Statistical analysis; Drafting of the manuscript; revised the manuscript for intellectual content
David Guarin	Massachusetts General Hospital	Concept and design; Acquisition, analysis, or interpretation of data; revised the manuscript for intellectual content
**Niyaz Mohammadzadehhonarvar**	Massachusetts General Hospital	Acquisition, analysis, or interpretation of data; revised the manuscript for intellectual content
Xiqun Chen, MD, PhD	Massachusetts General Hospital	Concept and design; Acquisition, analysis, or interpretation of data; Drafting of the manuscript; revised the manuscript for intellectual content
Xiang Gao, MD, PhD	The Pennsylvania State University	Concept and design; Acquisition, analysis, or interpretation of data; Drafting of the manuscript; revised the manuscript for intellectual content

References

1. Zafar S, Yaddanapudi SS. Parkinson Disease. StatPearls. Treasure Island (FL): StatPearls Publishing Copyright © 2021, StatPearls Publishing LLC. 2021.

For peer review only - http://bmjopen.bmj.com/site/about/guidelines.xhtml

2. Mappin-Kasirer B, Pan H, Lewington S, et al. Tobacco smoking and the risk of Parkinson disease: A 65-year follow-up of 30,000 male British doctors. *Neurology* 2020;94(20):e2132-e38.
3. Lesage S, Brice A. Parkinson's disease: from monogenic forms to genetic susceptibility factors. *Hum Mol Genet* 2009;18(R1):R48-59. doi: 10.1093/hmg/ddp012 [published Online First: 2009/03/20]
4. Ferlay J, Colombet M, Soerjomataram I, et al. Estimating the global cancer incidence and mortality in 2018: GLOBOCAN sources and methods. *International journal of cancer* 2019;144(8):1941-53.
5. Ejma M, Madetko N, Brzecka A, et al. The Links between Parkinson's Disease and Cancer. *Biomedicines* 2020;8(10) doi: 10.3390/biomedicines8100416 [published Online First: 2020/10/18]
6. Kendal WS. Dying with cancer: the influence of age, comorbidity, and cancer site. *Cancer* 2008;112(6):1354-62. doi: 10.1002/cncr.23315 [published Online First: 2008/02/21]
7. Mappin-Kasirer B, Pan H, Lewington S, et al. Tobacco smoking and the risk of Parkinson disease. *Neurology* 2020;94(20):e2132. doi: 10.1212/WNL.0000000000009437
8. Feng DD, Cai W, Chen X. The associations between Parkinson's disease and cancer: the plot thickens. *Translational neurodegeneration* 2015;4:20-20. doi: 10.1186/s40035-015-0043-z
9. Huang P, Yang XD, Chen SD, et al. The association between Parkinson's disease and melanoma: a systematic review and meta-analysis. *Transl Neurodegener* 2015;4:21. doi: 10.1186/s40035-015-0044-y [published Online First: 2015/11/05]
10. Bajaj A, Driver JA, Schernhammer ES. Parkinson's disease and cancer risk: a systematic review and meta-analysis. *Cancer Causes Control* 2010;21(5):697-707. doi: 10.1007/s10552-009-9497-6 [published Online First: 2010/01/08]
11. Walker E, Hernandez AV, Kattan MW. Meta-analysis: Its strengths and limitations. *Cleve Clin J Med* 2008;75(6):431-9. doi: 10.3949/ccjm.75.6.431 [published Online First: 2008/07/04]
12. Ben-Shlomo Y, Marmot M. Survival and cause of death in a cohort of patients with parkinsonism: possible clues to aetiology? *Journal of Neurology, Neurosurgery & Psychiatry* 1995;58(3):293-99.
13. Hely MA, Morris JG, Traficante R, et al. The sydney multicentre study of Parkinson's disease: progression and mortality at 10 years. *Journal of neurology, neurosurgery, and psychiatry* 1999;67(3):300-07. doi: 10.1136/jnnp.67.3.300
14. Higgins JP, Thompson SG, Deeks JJ, et al. Measuring inconsistency in meta-analyses. *Bmj* 2003;327(7414):557-60.
15. Wells GA, Shea B, O'Connell Da, et al. The Newcastle-Ottawa Scale (NOS) for assessing the quality of nonrandomised studies in meta-analyses: Oxford, 2000.
16. Lushniak BD, Samet JM, Pechacek TF, et al. The health consequences of smoking—50 years of progress: A report of the surgeon general. 2014
17. Baade PD, Fritschi L, Freedman DM. Mortality due to amyotrophic lateral sclerosis and Parkinson's disease among melanoma patients. *Neuroepidemiology* 2007;28(1):16-20. doi: 10.1159/000097851 [published Online First: 2006/12/14]
18. Becker C, Brobert GP, Johansson S, et al. Cancer risk in association with Parkinson disease: a population-based study. *Parkinsonism Relat Disord* 2010;16(3):186-90. doi: 10.1016/j.parkreldis.2009.11.005 [published Online First: 2009/12/01]
19. Bermejo-Pareja F, Benito-Leon J, Trincado R, et al. Neurodegenerative Diseases (Alzheimer and Parkinson), but Not Vascular Dementia, Are Negatively Associated to Cancer as Cause of Death in the NEDICES Cohort (P01.086). *Neurology* 2012;78(Meeting Abstracts 1):P01.086-P01.86. doi: 10.1212/WNL.78.1_MeetingAbstracts.P01.086
20. Bertoni JM, Arlette JP, Fernandez HH, et al. Increased melanoma risk in Parkinson disease: a prospective clinicopathological study. *Arch Neurol* 2010;67(3):347-52. doi: 10.1001/archneurol.2010.1 [published Online First: 2010/03/10]

For peer review only - http://bmjopen.bmj.com/site/about/guidelines.xhtml

21. Binaghi E, Mascia MM, Solla P, et al. Correlation between Parkinson's disease and malignant tumors. *Movement Disorders* 2016;31:S631-S32.
22. Boursi B, Mamtani R, Haynes K, et al. Parkinson's disease and colorectal cancer risk-A nested case control study. *Cancer Epidemiol* 2016;43:9-14. doi: 10.1016/j.canep.2016.05.007 [published Online First: 2016/05/28]
23. Constantinescu R, Elm J, Auinger P, et al. Malignant melanoma in early-treated Parkinson's disease: the NET-PD trial. *Mov Disord* 2014;29(2):263-5. doi: 10.1002/mds.25734 [published Online First: 2013/12/11]
24. Constantinescu R, Romer M, Kieburtz K, et al. Malignant melanoma in early Parkinson's disease: the DATATOP trial. *Mov Disord* 2007;22(5):720-2. doi: 10.1002/mds.21273 [published Online First: 2007/03/22]
25. Cui X, Liew Z, Hansen J, et al. Cancers Preceding Parkinson's Disease after Adjustment for Bias in a Danish Population-Based Case-Control Study. *Neuroepidemiology* 2019;52(3-4):136-43. doi: 10.1159/000494292 [published Online First: 2019/01/21]
26. Dalvin LA, Damento GM, Yawn BP, et al. Parkinson Disease and Melanoma: Confirming and Reexamining an Association. *Mayo Clin Proc* 2017;92(7):1070-79. doi: 10.1016/j.mayocp.2017.03.014 [published Online First: 2017/07/10]
27. D'Amelio M, Ragonese P, Morgante L, et al. Tumor diagnosis preceding Parkinson's disease: a case-control study. *Mov Disord* 2004;19(7):807-11. doi: 10.1002/mds.20123 [published Online First: 2004/07/16]
28. Driver JA, Kurth T, Buring JE, et al. Prospective case-control study of nonfatal cancer preceding the diagnosis of Parkinson's disease. *Cancer Causes Control* 2007;18(7):705-11. doi: 10.1007/s10552-007-9005-9 [published Online First: 2007/06/15]
29. Driver JA, Kurth T, Buring JE, et al. Parkinson disease and risk of mortality: a prospective comorbidity-matched cohort study. *Neurology* 2008;70(16 Pt 2):1423-30. doi: 10.1212/01.wnl.0000310414.85144.ee [published Online First: 2008/04/17]
30. Driver JA, Logroscino G, Buring JE, et al. A prospective cohort study of cancer incidence following the diagnosis of Parkinson's disease. *Cancer Epidemiol Biomarkers Prev* 2007;16(6):1260-5. doi: 10.1158/1055-9965.EPI-07-0038 [published Online First: 2007/06/06]
31. Elbaz A, Peterson BJ, Bower JH, et al. Risk of cancer after the diagnosis of Parkinson's disease: a historical cohort study. *Mov Disord* 2005;20(6):719-25. doi: 10.1002/mds.20401 [published Online First: 2005/02/11]
32. Elbaz A, Peterson BJ, Yang P, et al. Nonfatal cancer preceding Parkinson's disease: a case-control study. *Epidemiology* 2002;13(2):157-64. doi: 10.1097/00001648-200203000-00010 [published Online First: 2002/03/07]
33. Fall PA, Saleh A, Fredrickson M, et al. Survival time, mortality, and cause of death in elderly patients with Parkinson's disease: a 9-year follow-up. *Mov Disord* 2003;18(11):1312-6. doi: 10.1002/mds.10537 [published Online First: 2003/11/26]
34. Ferreira J, Silva JM, Freire R, et al. Skin cancers and precancerous lesions in Parkinson's disease patients. *Mov Disord* 2007;22(10):1471-5. doi: 10.1002/mds.21575 [published Online First: 2007/05/23]
35. Fois AF, Wotton CJ, Yeates D, et al. Cancer in patients with motor neuron disease, multiple sclerosis and Parkinson's disease: record linkage studies. *J Neurol Neurosurg Psychiatry* 2010;81(2):215-21. doi: 10.1136/jnnp.2009.175463 [published Online First: 2009/09/04]
36. Freedman DM, Pfeiffer RM. Associations Between Parkinson Disease and Cancer in US Asian Americans. *Jama Oncology* 2016;2(8):1093-94. doi: 10.1001/jamaoncol.2016.0729

For peer review only

For peer review only - http://bmjopen.bmj.com/site/about/guidelines.xhtml

37. Freedman DM, Travis LB, Gridley G, et al. Amyotrophic lateral sclerosis mortality in 1.9 million US cancer survivors. *Neuroepidemiology* 2005;25(4):176-80. doi: 10.1159/000087447 [published Online First: 2005/08/17]
38. Freedman DM, Wu J, Chen H, et al. Associations between cancer and Parkinson's disease in U.S. elderly adults. *Int J Epidemiol* 2016;45(3):741-51. doi: 10.1093/ije/dyw016 [published Online First: 2016/03/19]
39. Gorell JM, Johnson CC, Rybicki BA. Parkinson's disease and its comorbid disorders: an analysis of Michigan mortality data, 1970 to 1990. *Neurology* 1994;44(10):1865-8. doi: 10.1212/wnl.44.10.1865 [published Online First: 1994/10/01]
40. Jamrozik Z, Janik P, Friedman A, et al. Incidence of prostate cancer in patients with Parkinson's disease. *European Journal of Neurology* 2005;12:98-98.
41. Jansson B, Jankovic J. Low cancer rates among patients with Parkinson's disease. *Ann Neurol* 1985;17(5):505-9. doi: 10.1002/ana.410170514 [published Online First: 1985/05/01]
42. Jespersen CG, Norgaard M, Borre M. Parkinson's disease and risk of prostate cancer: A Danish population-based case-control study, 1995-2010. *Cancer Epidemiol* 2016;45:157-61. doi: 10.1016/j.canep.2016.11.002 [published Online First: 2016/11/14]
43. Kareus SA, Figueroa KP, Cannon-Albright LA, et al. Shared predispositions of parkinsonism and cancer: a population-based pedigree-linked study. *Arch Neurol* 2012;69(12):1572-7. doi: 10.1001/archneurol.2012.2261 [published Online First: 2012/09/05]
44. Kelm RC, K. P, J. LS, et al. Melanoma and Parkinson disease: A large, urban, single-center, midwestern U.S. population study. *Journal of the American Academy of Dermatology* 2018;79(3) doi: 10.1016/j.jaad.2018.05.765
45. Lai SW, Liao KF, Lin CH, et al. Parkinson's disease and lung cancer: a population-based case-control study in Taiwan. *Geriatr Gerontol Int* 2013;13(1):238-40. doi: 10.1111/j.1447-0594.2012.00919.x [published Online First: 2013/01/05]
46. Lai SW, Lin CL, Liao KF, et al. Parkinson's disease and hepatocellular carcinoma in older people: A population-based case-control study in Taiwan. *International Medical Journal* 2015;22(4):313-14.
47. Lerman S, Amichai B, Weinstein G, et al. Parkinson's Disease, Melanoma, and Keratinocyte Carcinoma: A Population-Based Study. *Neuroepidemiology* 2018;50(3-4):168-73. doi: 10.1159/000487855 [published Online First: 2018/03/23]
48. Liao KF, Lin CL, Lai SW. Parkinson's disease and risk of colorectal cancer: A population-based case-control study in Taiwan. *Neurology Asia* 2017;22(2):133-38.
49. Liao KF, Lin CL, Lai SW, et al. Parkinson's disease and risk of pancreatic cancer: a population-based case-control study in Taiwan. *Neurology Asia* 2015;20(3):251-55.
50. Lin PY, Chang SN, Hsiao TH, et al. Association Between Parkinson Disease and Risk of Cancer in Taiwan. *JAMA Oncol* 2015;1(5):633-40. doi: 10.1001/jamaoncol.2015.1752 [published Online First: 2015/07/17]
51. Lo RY, Tanner CM, Van Den Eeden SK, et al. Comorbid cancer in Parkinson's disease. *Mov Disord* 2010;25(12):1809-17. doi: 10.1002/mds.23246 [published Online First: 2010/07/30]
52. Minami Y, Yamamoto R, Nishikouri M, et al. Mortality and cancer incidence in patients with Parkinson's disease. *J Neurol* 2000;247(6):429-34. doi: 10.1007/s004150070171 [published Online First: 2000/08/10]
53. Naghavi-Behzad M, Piri R. Risk of cancer among patients with Parkinson disease. *Parkinsonism Relat Disord* 2016;22:e34.

For peer review only - http://bmjopen.bmj.com/site/about/guidelines.xhtml

54. Olsen JH, Friis S, Frederiksen K. Malignant melanoma and other types of cancer preceding Parkinson disease. *Epidemiology* 2006;17(5):582-7. doi: 10.1097/01.ede.0000229445.90471.5e [published Online First: 2006/07/14]
55. Olsen JH, Friis S, Frederiksen K, et al. Atypical cancer pattern in patients with Parkinson's disease. *Br J Cancer* 2005;92(1):201-5. doi: 10.1038/sj.bjc.6602279 [published Online First: 2004/12/08]
56. Olsen JH, Tangerud K, Wermuth L, et al. Treatment with levodopa and risk for malignant melanoma. *Mov Disord* 2007;22(9):1252-7. doi: 10.1002/mds.21397 [published Online First: 2007/05/31]
57. Ong EL, Goldacre R, Goldacre M. Differential risks of cancer types in people with Parkinson's disease: a national record-linkage study. *Eur J Cancer* 2014;50(14):2456-62. doi: 10.1016/j.ejca.2014.06.018 [published Online First: 2014/07/30]
58. Park JH, Kim DH, Park YG, et al. Cancer risk in patients with Parkinson's disease in South Korea: A nationwide, population-based cohort study. *Eur J Cancer* 2019;117:5-13. doi: 10.1016/j.ejca.2019.04.033 [published Online First: 2019/06/24]
59. Peretz C, Gurel R, Rozani V, et al. Cancer incidence among Parkinson's disease patients in a 10-yrs time-window around disease onset: A large-scale cohort study. *Parkinsonism Relat Disord* 2016;28:68-72. doi: 10.1016/j.parkreldis.2016.04.028 [published Online First: 2016/05/11]
60. Agalliu I, Ortega RA, Luciano MS, et al. Cancer outcomes among Parkinson's disease patients with leucine rich repeat kinase 2 mutations, idiopathic Parkinson's disease patients, and nonaffected controls. *Mov Disord* 2019;34(9):1392-98. doi: 10.1002/mds.27807 [published Online First: 2019/07/28]
61. Ording AG, Veres K, Horváth-Puhó E, et al. Alzheimer's and Parkinson's Diseases and the Risk of Cancer: A Cohort Study. *J Alzheimers Dis* 2019;72(4):1269-77. doi: 10.3233/jad-190867 [published Online First: 2019/11/11]
62. Pinter B, Diem-Zangerl A, Wenning GK, et al. Mortality in Parkinson's disease: a 38-year follow-up study. *Mov Disord* 2015;30(2):266-9. doi: 10.1002/mds.26060 [published Online First: 2014/12/03]
63. Piri R, Naghavi-Behzad M. Association between cancers and risk of Parkinson disease: A cohort study. *Parkinsonism Relat Disord* 2016;22:e33.
64. Powers KM, Smith-Weller T, Franklin GM, et al. Diabetes, smoking, and other medical conditions in relation to Parkinson's disease risk. *Parkinsonism Relat Disord* 2006;12(3):185-9. doi: 10.1016/j.parkreldis.2005.09.004 [published Online First: 2005/12/21]
65. Pressley JC, Louis ED, Tang MX, et al. The impact of comorbid disease and injuries on resource use and expenditures in parkinsonism. *Neurology* 2003;60(1):87-93. doi: 10.1212/wnl.60.1.87 [published Online First: 2003/01/15]
66. Rugbjerg K, Friis S, Lassen CF, et al. Malignant melanoma, breast cancer and other cancers in patients with Parkinson's disease. *Int J Cancer* 2012;131(8):1904-11. doi: 10.1002/ijc.27443 [published Online First: 2012/01/27]
67. Ryu HJ, Park JH, Choi M, et al. Parkinson's disease and skin cancer risk: a nationwide population-based cohort study in Korea. *Journal of the European Academy of Dermatology and Venereology* 2020;34(12):2775-80.
68. Schwid SR, Bausch J, Oakes D, et al. Cancer incidence in a trial of an antiapoptotic agent for Parkinson's disease. *Mov Disord* 2010;25(12):1801-8. doi: 10.1002/mds.23006 [published Online First: 2010/07/30]
69. Shalaby SY, Louis ED. Increased Odds of Melanoma: Parkinson's Disease, Essential Tremor, Dystonia versus Controls. *Neuroepidemiology* 2016;46(2):128-36. doi: 10.1159/000443794 [published Online First: 2016/01/29]

For peer review only - http://bmjopen.bmj.com/site/about/guidelines.xhtml

70. Sun LM, Liang JA, Chang SN, et al. Analysis of Parkinson's disease and subsequent cancer risk in Taiwan: a nationwide population-based cohort study. *Neuroepidemiology* 2011;37(2):114-9. doi: 10.1159/000331489 [published Online First: 2011/10/12]
71. Tacik P, Curry S, Fujioka S, et al. Cancer in Parkinson's disease. *Parkinsonism Relat Disord* 2016;31:28-33. doi: 10.1016/j.parkreldis.2016.06.014 [published Online First: 2016/07/04]
72. Tang CF, Lu MK, Muo CH, et al. Increased risk of brain tumor in patients with Parkinson's disease: a nationwide cohort study in Taiwan. *Acta Neurol Scand* 2016;134(2):148-53. doi: 10.1111/ane.12524 [published Online First: 2015/10/29]
73. Vanacore N, Spila-Alegiani S, Raschetti R, et al. Mortality cancer risk in parkinsonian patients: a population-based study. *Neurology* 1999;52(2):395-8. doi: 10.1212/wnl.52.2.395 [published Online First: 1999/02/05]
74. Wing K, Douglas I, Bhaskaran K, et al. A Cohort Analysis of the Association between Parkinson's Disease and Cancer Using the UK General Practice Research Database. *Pharmacoepidemiology and Drug Safety* 2012;21:442-43.
75. Winter AC, Rist PM, Buring JE, et al. Prospective comorbidity-matched study of Parkinson's disease and risk of mortality among women. *Bmj Open* 2016;6(9):5. doi: 10.1136/bmjopen-2016-011888
76. Wirdefeldt K, Weibull CE, Chen H, et al. Parkinson's disease and cancer: A register-based family study. *Am J Epidemiol* 2014;179(1):85-94. doi: 10.1093/aje/kwt232 [published Online First: 2013/10/22]
77. Dinesh D, Lee JS, Gao X, et al. Skin conditions in early Parkinson's disease. *Parkinsonism & Related Disorders* 2021;84:40-46. doi: https://doi.org/10.1016/j.parkreldis.2021.01.018
78. Ryu HJ, Park JH, Choi M, et al. Parkinson's disease and skin cancer risk: a nationwide population-based cohort study in Korea. *J Eur Acad Dermatol Venereol* 2020;34(12):2775-80. doi: 10.1111/jdv.16462 [published Online First: 2020/04/15]
79. Zhang P, Liu B. Association between Parkinson's Disease and Risk of Cancer: A PRISMA-compliant Meta-analysis. *ACS Chem Neurosci* 2019;10(10):4430-39. doi: 10.1021/acschemneuro.9b00498 [published Online First: 2019/10/05]
80. Larsson SC, Carter P, Kar S, et al. Smoking, alcohol consumption, and cancer: A mendelian randomisation study in UK Biobank and international genetic consortia participants. *PLoS Med* 2020;17(7):e1003178. doi: 10.1371/journal.pmed.1003178 [published Online First: 2020/07/24]
81. Driver JA. Inverse association between cancer and neurodegenerative disease: review of the epidemiologic and biological evidence. *Biogerontology* 2014;15(6):547-57. doi: 10.1007/s10522-014-9523-2 [published Online First: 2014/08/13]
82. Ibanez K, Boullosa C, Tabares-Seisdedos R, et al. Molecular evidence for the inverse comorbidity between central nervous system disorders and cancers detected by transcriptomic meta-analyses. *PLoS Genet* 2014;10(2):e1004173. doi: 10.1371/journal.pgen.1004173 [published Online First: 2014/03/04]
83. Klus P, Cirillo D, Botta Orfila T, et al. Neurodegeneration and Cancer: Where the Disorder Prevails. *Sci Rep* 2015;5:15390. doi: 10.1038/srep15390 [published Online First: 2015/10/27]
84. Dube U, Ibanez L, Budde JP, et al. Overlapping genetic architecture between Parkinson disease and melanoma. *Acta Neuropathol* 2020;139(2):347-64. doi: 10.1007/s00401-019-02110-z [published Online First: 2019/12/18]
85. Gong Y, Zack TI, Morris LG, et al. Pan-cancer genetic analysis identifies PARK2 as a master regulator of G1/S cyclins. *Nat Genet* 2014;46(6):588-94. doi: 10.1038/ng.2981 [published Online First: 2014/05/06]

For peer review only - http://bmjopen.bmj.com/site/about/guidelines.xhtml

86. Gupta A, Anjomani-Virmouni S, Koundouros N, et al. PARK2 Depletion Connects Energy and Oxidative Stress to PI3K/Akt Activation via PTEN S-Nitrosylation. *Mol Cell* 2017;65(6):999-1013.e7. doi: 10.1016/j.molcel.2017.02.019 [published Online First: 2017/03/18]
87. Agalliu I, San Luciano M, Mirelman A, et al. Higher frequency of certain cancers in LRRK2 G2019S mutation carriers with Parkinson disease: a pooled analysis. *JAMA Neurol* 2015;72(1):58-65. doi: 10.1001/jamaneurol.2014.1973 [published Online First: 2014/11/18]
88. Feng DD, Cai W, Chen X. The associations between Parkinson's disease and cancer: the plot thickens. *Transl Neurodegener* 2015;4:20. doi: 10.1186/s40035-015-0043-z [published Online First: 2015/10/28]
89. Shu L, Zhang Y, Pan H, et al. Clinical Heterogeneity Among LRRK2 Variants in Parkinson's Disease: A Meta-Analysis. *Frontiers in Aging Neuroscience* 2018;10(283) doi: 10.3389/fnagi.2018.00283
90. Ruiz-Martínez J, de la Riva P, Rodríguez-Oroz MC, et al. Prevalence of cancer in Parkinson's disease related to R1441G and G2019S mutations in LRRK2. *Mov Disord* 2014;29(6):750-5. doi: 10.1002/mds.25778 [published Online First: 2013/12/21]
91. Ysselstein D, Nguyen M, Young TJ, et al. LRRK2 kinase activity regulates lysosomal glucocerebrosidase in neurons derived from Parkinson's disease patients. *Nature communications* 2019;10(1):5570. doi: 10.1038/s41467-019-13413-w [published Online First: 2019/12/06]
92. Lebovitz C, Wretham N, Osooly M, et al. Loss of Parkinson's susceptibility gene LRRK2 promotes carcinogen-induced lung tumorigenesis. *Scientific Reports* 2021;11(1):2097. doi: 10.1038/s41598-021-81639-0
93. Gao X, Simon KC, Han J, et al. Family history of melanoma and Parkinson disease risk. *Neurology* 2009;73(16):1286-91. doi: 10.1212/WNL.0b013e3181bd13a1 [published Online First: 2009/10/21]
94. Weissenrieder JS, Neighbors JD, Mailman RB, et al. Cancer and the Dopamine D(2) Receptor: A Pharmacological Perspective. *J Pharmacol Exp Ther* 2019;370(1):111-26. doi: 10.1124/jpet.119.256818 [published Online First: 2019/04/20]
95. Siple JF, Schneider DC, Wanlass WA, et al. Levodopa therapy and the risk of malignant melanoma. *Ann Pharmacother* 2000;34(3):382-5. doi: 10.1345/aph.19150 [published Online First: 2000/08/05]
96. Bose A, Petsko GA, Eliezer D. Parkinson's Disease and Melanoma: Co-Occurrence and Mechanisms. *J Parkinsons Dis* 2018;8(3):385-98. doi: 10.3233/JPD-171263 [published Online First: 2018/07/12]
97. Chen X, Feng D, Schwarzschild MA, et al. Red hair, MC1R variants, and risk for Parkinson's disease - a meta-analysis. *Ann Clin Transl Neurol* 2017;4(3):212-16. doi: 10.1002/acn3.381 [published Online First: 2017/03/10]
98. Gao X, Simon KC, Han J, et al. Genetic determinants of hair color and Parkinson's disease risk. *Ann Neurol* 2009;65(1):76-82. doi: 10.1002/ana.21535 [published Online First: 2009/02/06]
99. Fullard ME, Duda JE. A Review of the Relationship Between Vitamin D and Parkinson Disease Symptoms. *Frontiers in neurology* 2020;11:454-54. doi: 10.3389/fneur.2020.00454
100. Campbell MJ, Trump DL. Vitamin D Receptor Signaling and Cancer. *Endocrinology and metabolism clinics of North America* 2017;46(4):1009-38. doi: 10.1016/j.ecl.2017.07.007 [published Online First: 2017/09/29]
101. Chan CWH, Law BMH, So WKW, et al. Pharmacogenomics of breast cancer: highlighting CYP2D6 and tamoxifen. *J Cancer Res Clin Oncol* 2020;146(6):1395-404. doi: 10.1007/s00432-020-03206-w [published Online First: 2020/04/10]

For peer review only - http://bmjopen.bmj.com/site/about/guidelines.xhtml

102. Ur Rasheed MS, Mishra AK, Singh MP. Cytochrome P450 2D6 and Parkinson's Disease: Polymorphism, Metabolic Role, Risk and Protection. *Neurochem Res* 2017;42(12):3353-61. doi: 10.1007/s11064-017-2384-8 [published Online First: 2017/09/06]
103. Schrempf M, Haluza D, Simic S, et al. Is Multidirectional UV Exposure Responsible for Increasing Melanoma Prevalence with Altitude? A Hypothesis Based on Calculations with a 3D-Human Exposure Model. *International journal of environmental research and public health* 2016;13(10):961. doi: 10.3390/ijerph13100961
104. Gross A, Racette BA, Camacho-Soto A, et al. Use of medical care biases associations between Parkinson disease and other medical conditions. *Neurology* 2018;90(24):e2155-e65.
105. Mahajan A, Chirra M, Dwivedi AK, et al. Skin cancer may delay onset but not progression of Parkinson's disease: a nested case-control study. *Frontiers in neurology* 2020;11:406.

Table 1. Association between Parkinson disease and cancer.

	No. of publications	Pooled RR (95% CI)	P for significance	P for heterogeneity
**Total cancer**				
All full-text publications	33	0.82 (0.76, 0.88)	<0.001	<0.001
Including abstracts	37	0.80 (0.74, 0.86)	<0.001	<0.001
Excluding mortality studies	25	0.85 (0.79, 0.92)	<0.001	<0.001
Excluding self-report diagnosis	31	0.81 (0.75, 0.87)	<0.001	<0.001
**Smoking-related cancer[1]**	21	0.76 (0.67, 0.85)	<0.001	<0.001
**Non-smoking-related cancer[2]**	19	0.92 (0.84, 0.99)	0.03	<0.001
**Site-specific cancer**				
Melanoma	29	1.75 (1.43, 2.14)	<0.001	<0.001
Non-melanoma skin cancer	17	0.90 (0.60, 1.34)	0.60	<0.001
Lung cancer	20	0.62 (0.51, 0.75)	<0.001	<0.001
Colorectal cancer	20	0.82 (0.75, 0.90)	<0.001	<0.001
Breast cancer	15	1.02 (0.93, 1.12)	0.66	0.001
Prostate cancer	17	0.93 (0.83, 1.03)	0.18	<0.001

For peer review only - http://bmjopen.bmj.com/site/about/guidelines.xhtml

[1]Smoking-related cancer includes cancer of the lung, larynx, mouth, esophagus, throat, bladder, kidney, liver, stomach, pancreas, colon and rectum, and cervix, as well as acute myeloid leukemia;

[2]Non-smoking-related cancer includes all other cancer except for those listed as smoking-related;

RR, relative risk; CI, confidence interval.

Figure 1. Flow chart.

Figure 2. Association between Parkinson's disease and total cancer in 33 publications. The figure shows the estimates (ESs) and 95% confidence intervals (CIs) for each study and the pooled result from random-effects model. Studies are stratified by temporal relationship of Parkinson's disease and cancer. M: men; W: women.

Figure 3. Association between Parkinson's disease and (A) smoking-related cancers in 21 publications, and (B) non-smoking-related cancers in 19 publications. Figure shows the estimates (ESs) and 95% confidence intervals (CIs) for each study and the pooled result from random effects model. Studies are stratified by temporal relationship of Parkinson's disease and cancer. M: men; W: women; *: pooled risk estimates calculated from individual ES in original publication.

Figure 4. Association between Parkinson's disease and (A) melanoma in 29 publications, and (B) non-melanoma skin cancers in 17 publications. Figure shows the estimates (ESs) and 95% confidence intervals (CIs) for each study and the pooled result from random effects model. Studies are stratified by temporal relationship of Parkinson's disease and cancer. M: men; W: women; BCC: basal cell carcinoma; SCC: squamous cell carcinoma; y: years of age.

For peer review only

For peer review only - http://bmjopen.bmj.com/site/about/guidelines.xhtml

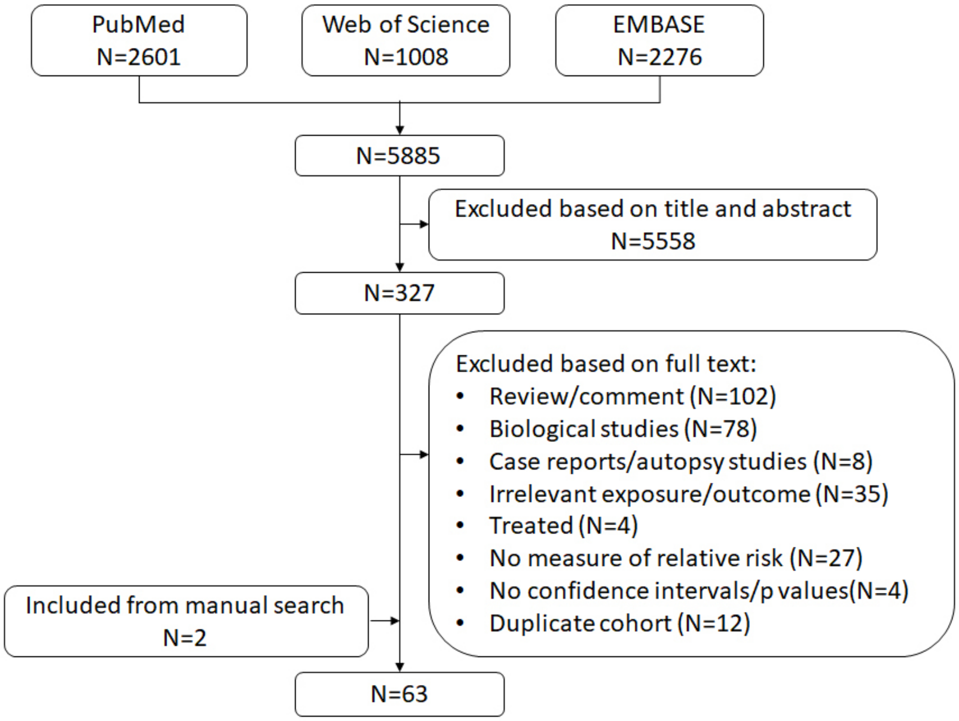

Figure 1. Flow chart.

314x242mm (72 x 72 DPI)

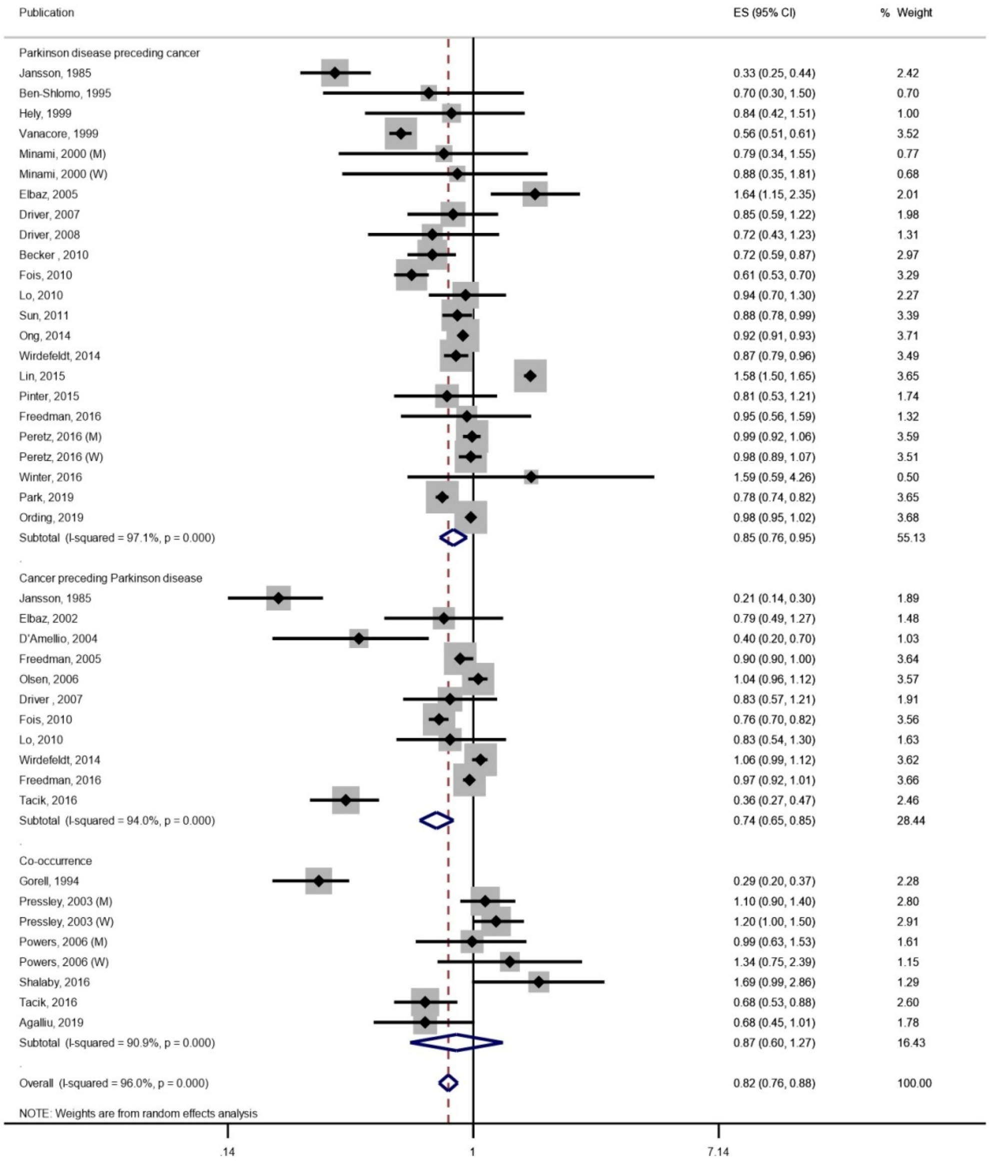

Figure 2. Association between Parkinson's disease and total cancer in 33 publications. The figure shows the estimates (ESs) and 95% confidence intervals (CIs) for each study and the pooled result from random-effects model. Studies are stratified by temporal relationship of Parkinson's disease and cancer. M: men; W: women.

239x283mm (150 x 150 DPI)

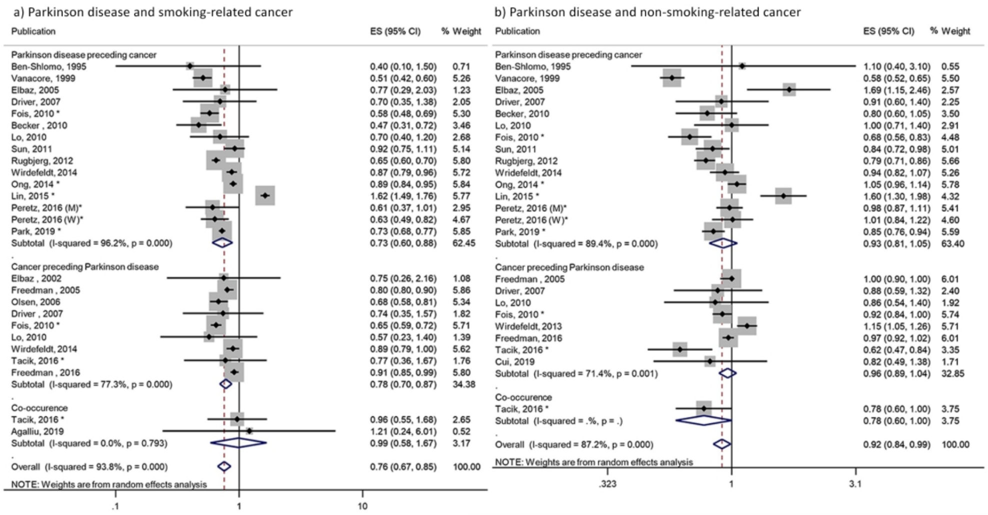

Figure 3. Association between Parkinson's disease and (A) smoking-related cancers in 21 publications, and (B) non-smoking-related cancers in 19 publications. Figure shows the estimates (ESs) and 95% confidence intervals (CIs) for each study and the pooled result from random effects model. Studies are stratified by temporal relationship of Parkinson's disease and cancer. M: men; W: women; *: pooled risk estimates calculated from individual ES in original publication.

763x406mm (72 x 72 DPI)

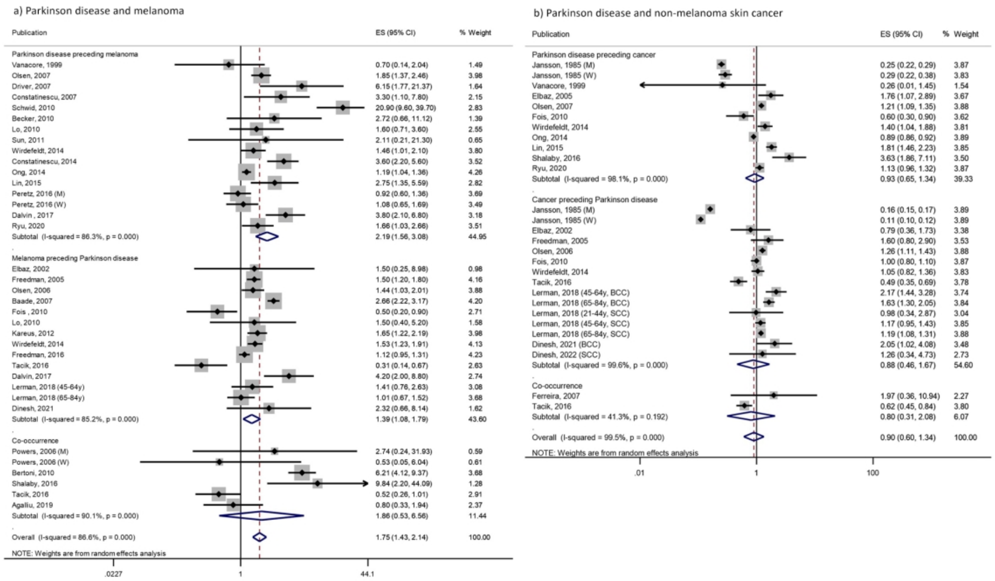

Figure 4. Association between Parkinson’s disease and (A) melanoma in 29 publications, and (B) non-melanoma skin cancers in 17 publications. Figure shows the estimates (ESs) and 95% confidence intervals (CIs) for each study and the pooled result from random effects model. Studies are stratified by temporal relationship of Parkinson’s disease and cancer. M: men; W: women; BCC: basal cell carcinoma; SCC: squamous cell carcinoma; y: years of age.

737x427mm (72 x 72 DPI)

Supplementary material for "Parkinson Disease and cancer: a systematic review and meta-analysis of 17,697,252 participants"

Content

Supplementary methods

Search strategy

Duplicate database inclusion/exclusion

Supplementary tables

Table 1. Characteristics of publications included in meta-analysis of Parkinson Disease and cancer.

Table 2. Meta regression and sub-group analysis on association between Parkinson disease and total cancer.

Table 3. Publications on risk of total cancer associated with levodopa treatment.

Supplementary figures

Figure 1. Funnel plot of studies of the association between Parkinson Disease and total cancer.

Figure 2. Association between use of levodopa and risk of total cancer in 4 publications.

Figure 3. Funnel plot of studies of the association between Parkinson Disease and a) smoking-related cancers, b) non-smoking-related cancers.

Figure 4. Funnel plot of studies of the association between Parkinson Disease and a) melanoma, b) non-melanoma skin cancers.

For peer review only - http://bmjopen.bmj.com/site/about/guidelines.xhtml

Supplementary methods

PubMed search strategy for Parkinson Disease and cancer

(("Parkinson Disease"[Mesh] OR "Parkinson Disease"[TW] OR "Parkinson's Disease"[TW] OR "Parkinsonism"[TW]) AND "cancer"[sb] AND ("Epidemiologic Studies"[Mesh] OR "Epidemiologic"[TW] OR "epidemiological"[TW] OR "Case-Control Studies"[Mesh] OR "case-control"[TW] OR "case control"[TW] OR "Case-Comparison"[TW] OR "Case Comparison"[TW] OR "Case-Compeer"[TW] OR "Case-Referent"[TW] OR "Case Referent"[TW] OR "Case-Base"[TW] OR "Case Base"[TW] OR "Cohort Studies"[Mesh] OR "cohort"[TW] OR "Concurrent"[TW] OR "Incidence"[TW] OR "Cross-Sectional Studies"[Mesh] OR "cross-sectional"[TW] OR "cross sectional"[TW] OR "Disease Frequency"[TW] OR "Prevalence"[TW] OR "Follow-Up Studies"[Mesh] OR "Follow-Up"[TW] OR "Follow Up"[TW] OR "Followup"[TW] OR "Longitudinal Studies"[Mesh] OR "longitudinal"[TW] OR "Retrospective Studies"[Mesh] OR "retrospective"[TW] OR "Prospective Studies"[Mesh] OR "prospective"[TW] OR "observational"[TW] OR "Observational Study" [Publication Type] OR "mortality studies"[TW] OR "ratio"[TW] OR "risk"[TW]) AND English[lang]) NOT ("animals"[MeSH Terms] NOT ("humans"[MeSH Terms] AND "animals"[MeSH Terms]))

Selection of publications that used same population

Leibson 2006, Elbaz 2002, Elbaz 2005, and Dalvin 2017 all used data from Mayo Clinic, Minnesota. Leibson 2006 was updated by Elbaz 2002 and 2005, therefore excluded from this meta-analysis. Elbaz 2002 studied PD risk after cancer, while Elbaz 2005 studied cancer risk after PD, therefore both publications were included. Dalvin 2017 was a cross-sectional extension of previous result, but it contained detailed analysis on melanoma, therefore it was not included in the analysis for total cancer, but was included for melanoma.

Olsen 2005, Olsen 2006, Olsen 2007, Rugbjerg 2012, Frandson 2014, Jespersen 2016, Cui 2019, and Ording 2019 all used National Hospital Register of Denmark. Frandson 2014 was a cross-sectional study that overlapped with Olsen 2006, Rugbjerg 2012, and Ording 2019, therefore was not included in this meta-analysis. Other publications varied in designs, time windows, temporal relationship, and cancer of interest.

For peer review only - http://bmjopen.bmj.com/site/about/guidelines.xhtml

There were also multiple publications from Physicians' Health Study, Women's Health Study, and Taiwan Health Registry. However, all of these groups of paper varied in designs, time windows, temporal relationship, and cancer of interest, therefore were not considered as duplicates. Other duplicates were meeting proceedings/abstracts of later-published articles, or duplicates that were not identified by matching in Endnote X9.

For peer review only

For peer review only - http://bmjopen.bmj.com/site/about/guidelines.xhtml

Supplementary tables

Table 1. Characteristics of publications included in meta-analysis of Parkinson Disease (PD) and cancer.

Author	Year	Publication type	Temporal direction	Study design	Location	Cohort	Case N	Control N	Follow time, y	Disease ascertainment	Smoking adjusted	Quality score
Agalliu	2019	Article	Co-occurrence	Cross-sectional	Europe, Israel, and the United States	Michael J. Fox Foundation	712	218	\	Diagnosed; idiopathic PD	No	5
Baade	2007	Article	Cancer preceding PD	Case-only cohort	Australia		127037		6.0	Coded	No	5
Becker	2010	Article	PD preceding cancer	1. Matched cohort 2. Case-control	UK	UK-based General Practice Research Database	466	1864	\	Validated; idiopathic PD	Yes	9
Ben-Shlomo	1995	Article	PD preceding cancer	Matched cohort	England and Wales	Second National Morbidity Study	220	421	\	Coded	No	7
Bermejo-Pareja	2012	Abstract	PD preceding cancer	Prospective cohort	Spain	Neurologic Disorders in Central Spain (NEDICES)	81	5197	\	\	No	\
Bertoni	2010	Article	Co-occurence	Case-only cohort	North America		2106		\	Diagnosed	No	7
Binagh	2016	Abstract	Co-occurence	Cross-sectional	Italy		529		\	Diagnosed	Yes	\
Boursi	2016	Article	PD preceding cancer	Case-control	UK	The Health Improvement Network	22093	85833	\	Diagnosed	Yes	9
Constatinescu	2007	Article	PD preceding cancer	Case-only cohort	North America	DATATOP	800		4.61	Diagnosed; idiopathic PD	No	4
Constatinescu	2014	Article	PD preceding cancer	Case-only cohort	US	NET-PD	1737		3.71	Diagnosed; idiopathic PD	No	4

For peer review only - http://bmjopen.bmj.com/site/about/guidelines.xhtml

Author	Year	Publication type	Temporal direction	Study design	Location	Cohort	Case N	Control N	Follow time, y	Disease ascertainment	Smoking adjusted	Quality score
Cui	2019	Article	Cancer preceding PD	Case-control	Denmark	National Hospital Register	1813	1887	\	Diagnosed; idiopathic PD	Yes	9
Dalvin	2017	Article	Both	1. Case-control 2. Matched cohort	Minnesota	Mayo clinic	974	2922	5	Coded	No	7
D'Amellio	2004	Article	Cancer preceding PD	Case-control	Italy		222	222	\	Diagnosed; idiopathic PD	Yes	8
Dinesh	2021	Article	Cancer preceding PD	Case-control	US	PPMI database	423	196	\	Diagnosed	No	8
Driver	2007	Article	PD preceding cancer	Matched cohort	US	Physicians' Health Study	487	487	5.2	Validated; idiopathic PD	Yes	8
Driver	2007	Article	Cancer preceding PD	Case-control	US	Physicians' Health Study	487	487	\	Validated; idiopathic PD	Yes	8
Driver	2008	Article	PD preceding cancer	Matched cohort	US	Physicians' Health Study	560	560	5.8	Validated; idiopathic PD	Yes	8
Elbaz	2002	Article	Cancer preceding PD	Case-control	Minnesota	Mayo clinic	196	196	5.5	Diagnosed	No	7
Fall	2003	Article	PD preceding cancer	Matched cohort	Sweden		170	510	4.8	Diagnosed	No	8
Elbaz	2005	Article	PD preceding cancer	Matched cohort	Minnesota	Mayo clinic	196	185	8	Diagnosed	Yes	6
Ferreira	2007	Article	Co-occurence	Cross-sectional	Portugal	The Lisbon University Hospital	150	146	\	Diagnosed; idiopathic PD	No	5
Fois	2010	Article	Both	Case-only cohort	UK	Oxford Record Linkage Study	4355		3.2	Coded	No	7

For peer review only - http://bmjopen.bmj.com/site/about/guidelines.xhtml

Author	Year	Publication type	Temporal direction	Study design	Location	Cohort	Case N	Control N	Follow time, y	Disease ascertainment	Smoking adjusted	Quality score
Freedman	2005	Article	Cancer preceding PD	Case-only cohort	US	SEER-Medicare	1900000		8.5	Coded	No	6
Freedman	2016	Letter	PD preceding cancer	Case-control	US (Asian Americans)	SEER-Medicare	20627	5558	\	Coded	No	7
Freedman	2016	Article	Cancer preceding PD	1. Case-control 2. cohort	US	SEER-Medicare	743779	419432	2.8	Coded	No	7
Gorell	1994	Article	Co-occurence	Cross-sectional	Michigan		8629	208933	\	Coded	No	7
Hely	1999	Article	PD preceding cancer	Case-only cohort	Australia	Sydney Multicenter Study of PD	130		9.1	Diagnosed	No	5
Jansson	1985	Article	Both	Prospective cohort	US		406		8.6	Diagnosed	Yes	\
Jamrozik	2005	Abstract	Cancer preceding PD	Case-control	Poland		100	100	\	\	No	7
Jespersen	2016	Article	Co-occurence	Case-control	Denmark	National Registry	45429	227145	\	Coded	No	7
Kareus	2012	Article	Cancer preceding PD	Case-control	US	Utah Cancer Registry	2300000			Coded	No	6
Kelm	2018	Abstract	Co-occurence	Case-control	US	Northwestern Medicine Enterprise Data Warehouse medical record	4751	9494	5.75	Coded	No	\
Lai	2013	Letter	Co-occurence	Case-control	Taiwan	National Health	2822	11288	\	Coded	Yes	8
Lai	2015	Article	Co-occurence	Case-control	Taiwan	National Health	1815	7260	\	Coded	No	8

For peer review only - http://bmjopen.bmj.com/site/about/guidelines.xhtml

Author	Year	Publication type	Temporal direction	Study design	Location	Cohort	Case N	Control N	Follow time, y	Disease ascertainment	Smoking adjusted	Quality score
Lerman	2018	Article	PD preceding cancer	Prospective cohort	Isreal	Maccabi Health Services	7727	1243968	\	Coded	Yes	9
Liao	2015	Article	PD preceding cancer	Case-control	Taiwan	National Health	13861	55444	\	Coded	No	8
Liao	2017	Article	PD preceding cancer	Case-control	Taiwan	National Health	64619	64619	\	Coded	No	8
Lin	2015	Article	PD preceding cancer	Matched cohort	Taiwan	National Health	62023	124046	\	Coded	No	6
Lo	2010	Article	Both	Matched cohort	US	PEAK	692	761	5.0; 4.3	Diagnosed	Yes	7
Minami	2000	Article	PD preceding cancer	Case-only cohort	Japan		228		6.97	Validated	No	6
Naghavi-Behzad	2016	Abstract	PD preceding cancer	Case-control	Iran		\		\	\	No	\
Olsen	2005	Denmark	PD preceding cancer	National Hospital Register			14088		5.0	Coded; idiopathic PD	No	8
Olsen	2006	Article	Cancer preceding PD	Case-control	Denmark	National Hospital Register	8090	32320	\	Coded; idiopathic PD	No	6
Olsen	2007	Article	PD preceding cancer	Case-only cohort	Denmark	National Hospital Register	14088		\	Coded; idiopathic PD	No	6
Ong	2014	Article	PD preceding cancer	Prospective cohort	UK	NHS hospital	219194	9015614	\	Coded	No	8
Ording	2019	Article	PD preceding cancer	Case-only cohort	Denmark	National Hospital Register	28835		4.0	Coded	No	7
Park	2019	Article	PD preceding cancer	Matched cohort	South Korea	NHI	52009	260045	\	Coded	No	8

For peer review only

Author	Year	Publication type	Temporal direction	Study design	Location	Cohort	Case N	Control N	Follow time, y	Disease ascertainment	Smoking adjusted	Quality score
Peretz	2016	Article	PD preceding cancer	Case-only cohort	Israel	Maccabi Health Services	7125		10.5	Validated	No	6
Pinter	2015	Article	PD preceding cancer	Case-only cohort	Austria		237		14.8	Coded	No	6
Piri	2016	Abstract	PD preceding cancer	Prospective cohort		Cancer Registry Database	2584		\	Diagnosed	No	\
Powers	2006	Article	Co-occurence	Case-control	Seattle		352	484	\	Diagnosed; idiopathic PD	Yes	8
Pressley	2003	Article	Co-occurence	Cross-sectional	US	National Long-Term Care Survey	791	24040	\	Coded	No	6
Rugbjerg	2012	Article	PD preceding cancer	Case-only cohort	Denmark	National Hospital Register	20343		5.7	Coded	No	6
Ryu	2020	Article	PD preceding cancer	Matched cohort	Korea	South Korea National Health Insurance System	70780	353900	8	Diagnosed	No	7
Schwid	2010	Article	PD preceding cancer	Case-only cohort	US	PRECEPT	806		1.8	Diagnosed/verified	No	4
Shalaby	2016	Article	Co-occurence	Case-control	US	Columbia University Medical Cente	108	124	\	Self-report	No	6
Sun	2011	Article	PD preceding cancer	Matched cohort	Taiwan	NHI	4957	19828	\	Coded	No	8
Tacik	2016	Article	1. Co-occurrence 2. cancer preceding PD	Prospective cohort	Florida	Mayo clinic	971	478	4.6	Diagnosed	No	6

For peer review only

For peer review only - http://bmjopen.bmj.com/site/about/guidelines.xhtml

Author	Year	Publication type	Temporal direction	Study design	Location	Cohort	Case N	Control N	Follow time, y	Disease ascertainment	Smoking adjusted	Quality score
Tang	2016	Article	PD preceding cancer	Matched cohort	Taiwan	NHI	2998	11992	\	Coded	No	7
Vanacore	1999	Communication	PD preceding cancer	Case-only cohort	Italy		10322		5.7	Drug	No	4
Wing	2012	Abstract	Both	Prospective cohort	UK		8549	42160	\	\	Yes	\
Winter	2016	Article	PD preceding cancer	Matched cohort	US	Women's Health Study	396	396	6.2	Self-report	Yes	7
Wirdefeldt	2014	Article	Both	Matched cohort	Sweden		11786	58930	\	Coded	No	6

Study design and temporal direction was defined per each individual study definition, most of which was based on the diagnosis date of two diseases. Disease ascertainment was defined per the description of whether any physicians, neurologists or movement specialists made the diagnosis. Quality score was assessed by the Newcastle-Ottawa Scale for cohort studies and for case-control studies (range 0–9).

For peer review only - http://bmjopen.bmj.com/site/about/guidelines.xhtml

Table 2. Subgroup-analysis of the association between Parkinson disease and cancer.

	No. of publications	Pooled RR (95% CI)	P for significance	P for heterogeneity	P difference
**Age**					**0.10**
< 69.3 years	13	0.70 (0.42, 1.19)	0.21	<0.001	
≥ 69.3 years	14	0.90 (0.81, 1.00)	0.05	<0.001	
**Sex**					**0.31**
Men-dominant	23	0.76 (0.57, 1.02)	0.07	<0.001	
Women-dominant	12	0.91 (0.70, 1.17)	0.45	<0.001	
**Ethnicity**					**0.19**
Caucasian-dominant	27	0.75 (0.59, 0.96)	0.02	<0.001	
Asian-dominant	6	0.98 (0.75, 1.28)	0.88	<0.001	
**Study design**					**0.92**
Prospective cohort	24	0.79 (0.65, 0.96)	0.05	<0.001	
Other	9	0.79 (0.65, 0.96)	0.02	<0.001	
**Newcastle-Ottawa quality score**					**0.31**
≤ 6	12	0.87 (0.71, 1.08)	0.21	<0.001	
≥ 7	21	0.75 (0.57, 0.98)	0.04	<0.001	
**Period of study**					**0.19**
< 2010	16	0.73 (0.61, 0.88)	0.001	<0.001	
≥ 2010	17	0.88 (0.80, 0.96)	0.003	<0.001	

6 publications did not report mean/median age or age range.

2 publications did not report sex ratio. 4 publications separately report risk estimates for men and women, therefore counted in both sex groups.

For peer review only - http://bmjopen.bmj.com/site/about/guidelines.xhtml

Table 3. Publications on risk of total cancer associated with levodopa treatment.

**Publication**	**Estimation (95% confidence interval)**	**Note**
Elbaz, 2005	1.26 (0.39, 4.12)	4th (>1,313 g) compared to 1st quartile of cumulative levodopa
Constanescu, 2007	1.4 (0.3, 4.3)	After levodopa use
Olsen, 2007	1.0 (0.5, 2.0)	≥1370 g compared to 600-1369 g of cumulative levodopa
Becker, 2010	0.7 (0.56, 0.88)	≥5 prescription of levodopa

For peer review only

For peer review only - http://bmjopen.bmj.com/site/about/guidelines.xhtml

Supplementary figures

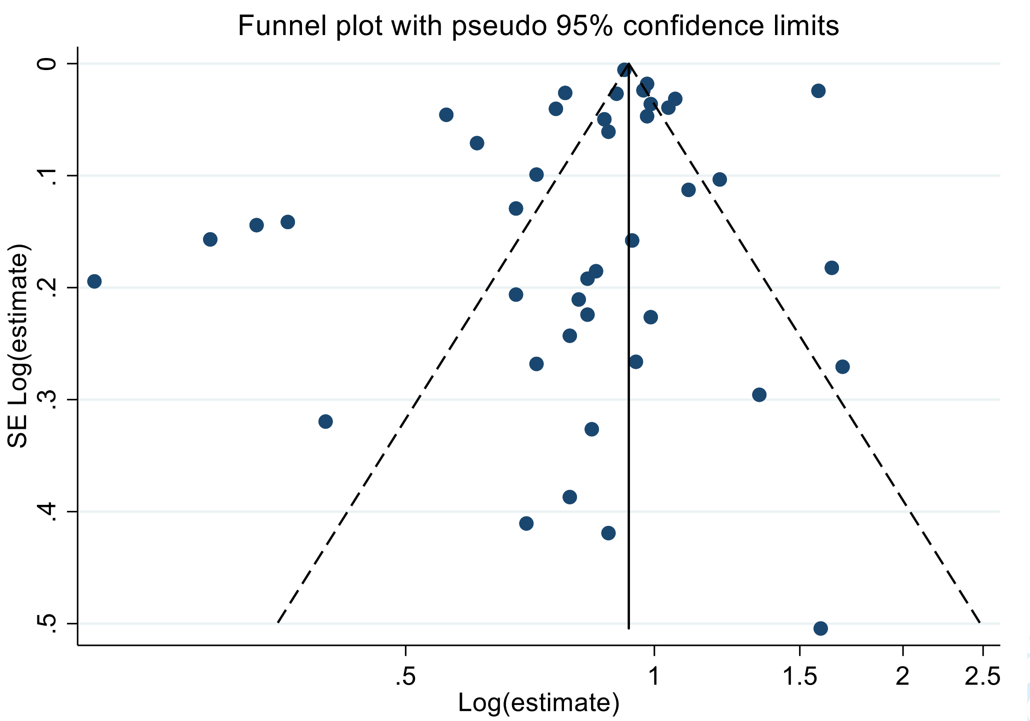

Figure 1. Funnel plot of studies of the association between Parkinson Disease and total cancer. The log-transformed risk estimates from each study is plotted on the horizontal axis, and its standard error is plotted on the vertical axis.

For peer review only - http://bmjopen.bmj.com/site/about/guidelines.xhtml

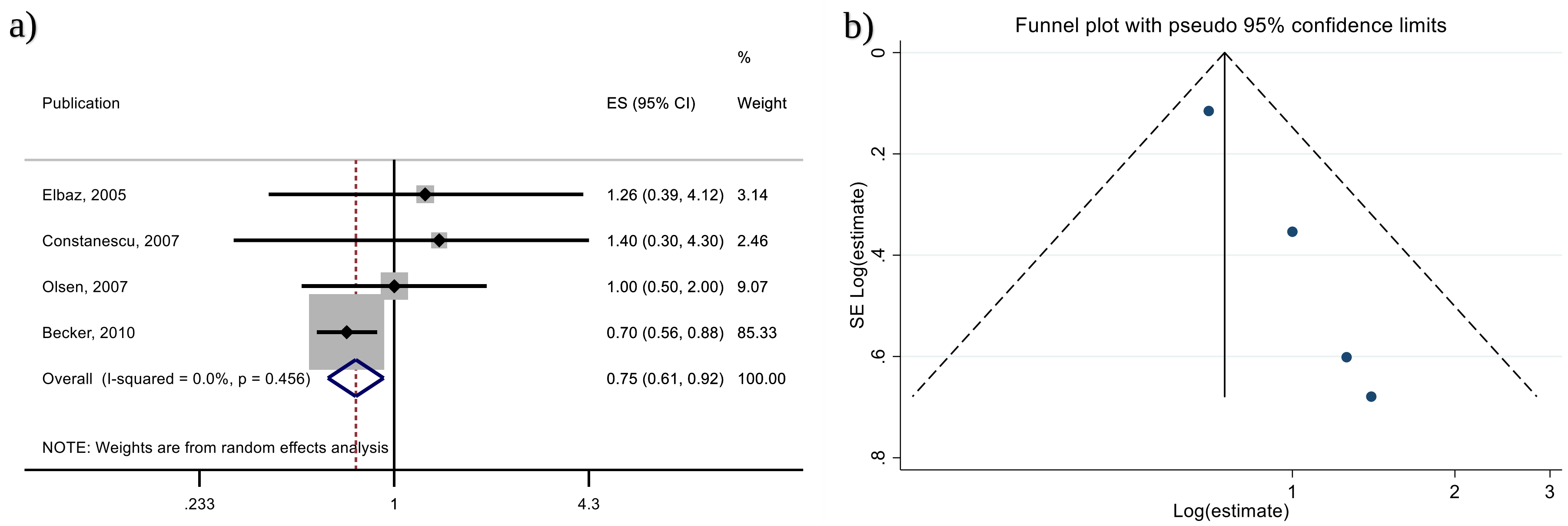

Figure 2. a) Individual and pooled estimates of the association between use of levodopa and risk of total cancer in 4 publications. Figure shows the estimates (ESs) and 95% confidence intervals (CIs) for each individual studies and the pooled result from random effects model. b) Funnel plots of these 4 publications.

For peer review only - http://bmjopen.bmj.com/site/about/guidelines.xhtml

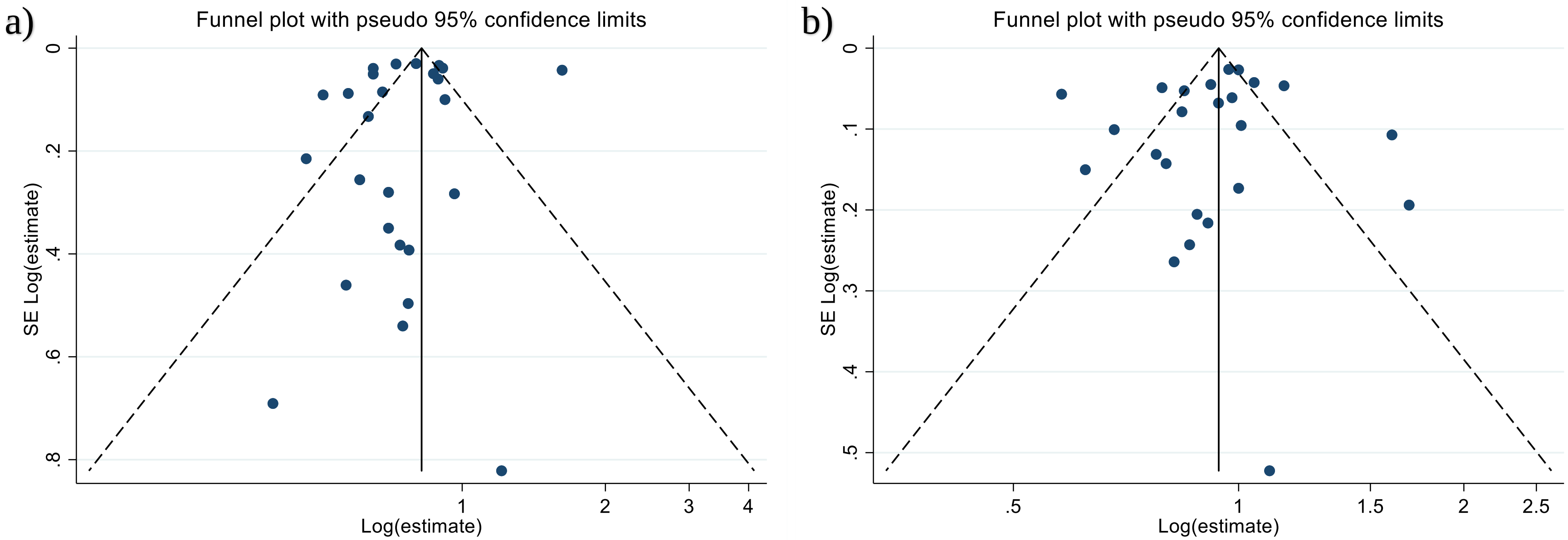

Figure 3. Funnel plot of studies of the association between Parkinson Disease and a) smoking-related cancers, b) non-smoking-related cancers. The log-transformed risk estimates from each study is plotted on the horizontal axis, and its standard error is plotted on the vertical axis.

review only

For peer review only - http://bmjopen.bmj.com/site/about/guidelines.xhtml

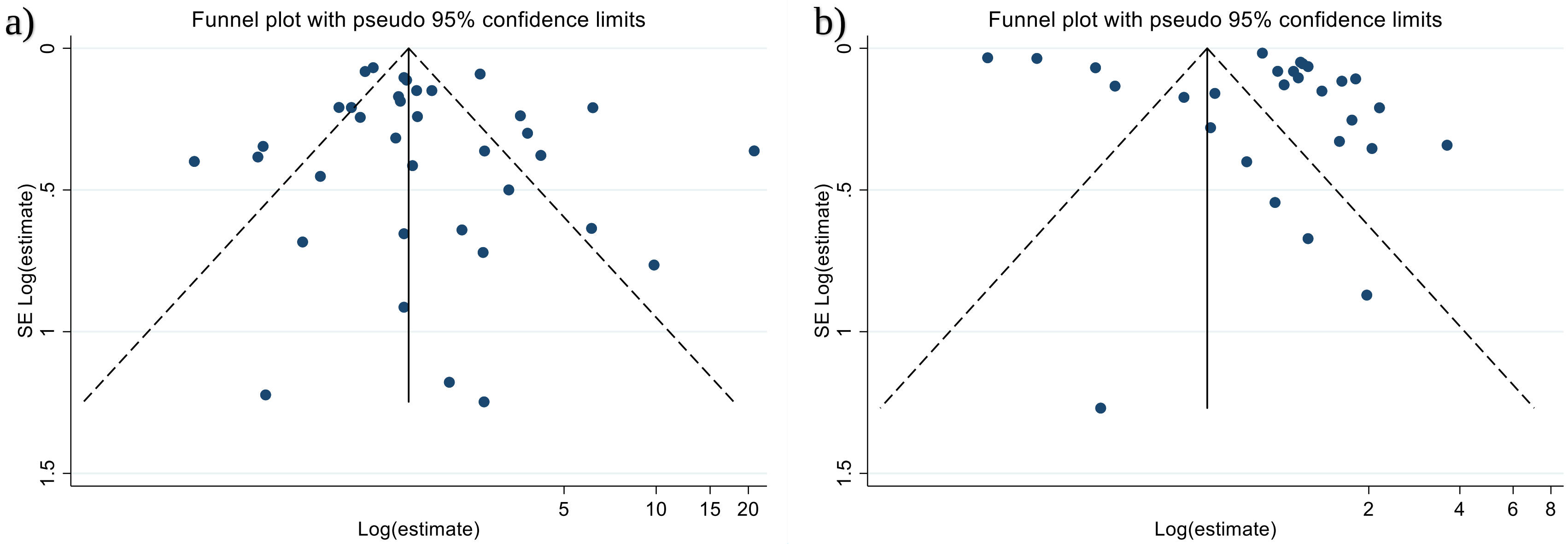

Figure 4. Funnel plot of studies of the association between Parkinson Disease and a) melanoma, b) non-melanoma skin cancers. The log-transformed risk estimates from each study is plotted on the horizontal axis, and its standard error is plotted on the vertical axis.

For peer review only - http://bmjopen.bmj.com/site/about/guidelines.xhtml

PRISMA 2009 Checklist

Section/topic	#	Checklist item	Reported on page #
**TITLE**			
Title	1	Identify the report as a systematic review, meta-analysis, or both.	1
**ABSTRACT**			
Structured summary	2	Provide a structured summary including, as applicable: background; objectives; data sources; study eligibility criteria, participants, and interventions; study appraisal and synthesis methods; results; limitations; conclusions and implications of key findings; systematic review registration number.	2 & 3
**INTRODUCTION**			
Rationale	3	Describe the rationale for the review in the context of what is already known.	5
Objectives	4	Provide an explicit statement of questions being addressed with reference to participants, interventions, comparisons, outcomes, and study design (PICOS).	5
**METHODS**			
Protocol and registration	5	Indicate if a review protocol exists, if and where it can be accessed (e.g., Web address), and, if available, provide registration information including registration number.	7 and supplementary material
Eligibility criteria	6	Specify study characteristics (e.g., PICOS, length of follow-up) and report characteristics (e.g., years considered, language, publication status) used as criteria for eligibility, giving rationale.	7
Information sources	7	Describe all information sources (e.g., databases with dates of coverage, contact with study authors to identify additional studies) in the search and date last searched.	7
Search	8	Present full electronic search strategy for at least one database, including any limits used, such that it could be repeated.	7
Study selection	9	State the process for selecting studies (i.e., screening, eligibility, included in systematic review, and, if applicable, included in the meta-analysis).	7
Data collection process	10	Describe method of data extraction from reports (e.g., piloted forms, independently, in duplicate) and any processes for obtaining and confirming data from investigators.	8
Data items	11	List and define all variables for which data were sought (e.g., PICOS, funding sources) and any assumptions and simplifications made.	8
Risk of bias in individual studies	12	Describe methods used for assessing risk of bias of individual studies (including specification of whether this was done at the study or outcome level), and how this information is to be used in any data synthesis.	8
Summary measures	13	State the principal summary measures (e.g., risk ratio, difference in means).	8

For peer review only

PRISMA 2009 Checklist

Synthesis of results	14	Describe the methods of handling data and combining results of studies, if done, including measures of consistency (e.g., I^2) for each meta-analysis.	9

Section/topic	#	Checklist item	Reported on page #
Risk of bias across studies	15	Specify any assessment of risk of bias that may affect the cumulative evidence (e.g., publication bias, selective reporting within studies).	8
Additional analyses	16	Describe methods of additional analyses (e.g., sensitivity or subgroup analyses, meta-regression), if done, indicating which were pre-specified.	9
**RESULTS**			
Study selection	17	Give numbers of studies screened, assessed for eligibility, and included in the review, with reasons for exclusions at each stage, ideally with a flow diagram.	10 and supplementary material
Study characteristics	18	For each study, present characteristics for which data were extracted (e.g., study size, PICOS, follow-up period) and provide the citations.	10, Table 1, and Supplementary material
Risk of bias within studies	19	Present data on risk of bias of each study and, if available, any outcome level assessment (see item 12).	10, 11, and supplementary material
Results of individual studies	20	For all outcomes considered (benefits or harms), present, for each study: (a) simple summary data for each intervention group (b) effect estimates and confidence intervals, ideally with a forest plot.	10, 11, Figure 1-4
Synthesis of results	21	Present results of each meta-analysis done, including confidence intervals and measures of consistency.	10, 11
Risk of bias across studies	22	Present results of any assessment of risk of bias across studies (see Item 15).	10, 11, and supplementary material
Additional analysis	23	Give results of additional analyses, if done (e.g., sensitivity or subgroup analyses, meta-regression [see Item 16]).	10
**DISCUSSION**			
Summary of evidence	24	Summarize the main findings including the strength of evidence for each main outcome; consider their relevance to key groups (e.g., healthcare providers, users, and policy makers).	12-15

For peer review only

PRISMA 2009 Checklist

Limitations	25	Discuss limitations at study and outcome level (e.g., risk of bias), and at review-level (e.g., incomplete retrieval of identified research, reporting bias).	12,14,15
Conclusions	26	Provide a general interpretation of the results in the context of other evidence, and implications for future research.	15
**FUNDING**			
Funding	27	Describe sources of funding for the systematic review and other support (e.g., supply of data); role of funders for the systematic review.	16

From: Moher D, Liberati A, Tetzlaff J, Altman DG, The PRISMA Group (2009). Preferred Reporting Items for Systematic Reviews and Meta-Analyses: The PRISMA Statement. PLoS Med 6(7): e1000097. doi:10.1371/journal.pmed1000097

For more information, visit: **www.prisma-statement.org**.

For peer review only

For peer review only - http://bmjopen.bmj.com/site/about/guidelines.xhtml
